# Intracellular Delivery of Native Proteins by BioReversible Arginine Modification (BioRAM) on Amino Groups

**DOI:** 10.1002/anie.202506802

**Published:** 2025-07-09

**Authors:** Jonathan Franke, Jan Vincent V. Arafiles, Christian Leis, Christian P. R. Hackenberger

**Affiliations:** ^1^ Department of Chemical Biology Leibniz‐Forschungsinstitut für Molekulare Pharmakologie (FMP) Robert‐Rössle‐Strasse 10 13125 Berlin Germany; ^2^ Department of Chemistry Humboldt Universität zu Berlin Brook‐Taylor‐Straße 2 12489 Berlin Germany

**Keywords:** Bioconjugation, Bioreversible modification, Cell‐penetrating peptides, Intracellular protein delivery, RNase A

## Abstract

Protein‐based tools are emerging as innovative solutions to interfere with biological pathways in molecular biology and medicine. They offer advantages over traditional small molecules due to their adaptable structural diversity and their ability to engage previously inaccessible cellular targets. However, most proteins do not penetrate the lipid bilayer of mammalian cells and are therefore restricted to extracellular targets. Despite recent advances, a general method for the delivery of functional proteins into human cells remains a significant challenge. In this study, we present a bioreversible protein modification strategy of amines using short arginine‐containing peptides (termed BioRAM) that enables cytosolic delivery starting from genetically non‐engineered proteins. We optimized the bioconjugation strategy to achieve fast intracellular cleavage and complete recovery of the native protein. In combination with our previously established cell‐penetrating peptide (CPP)‐additive protocol, we show superior delivery of fluorescent protein and functional RNase A into the cytosol, achieving physiological response. Moreover, we are able to demonstrate the excellent performance of BioRAM in the presence of serum, thereby broadening the scope for intracellular applications of functional proteins.

## Graphical abstract

**Bior**eversible **A**rginine **M**odification of amine‐containing protein residues (termed BioRAM) allows the serum‐compatible, readily accessible, and efficient delivery of native proteins into live cells. Combined with cell‐penetrating peptide (CPP) additives, BioRAM results in intracellular RNase A activity at nanomolar concentrations, thus enabling the cellular delivery of native protein cargoes without additional genetic engineering.

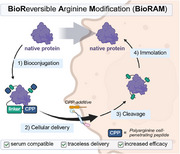

## Introduction

The intracellular delivery of proteins and antibodies is pivotal for advancing basic biochemical research and for developing biopharmaceuticals to target intracellular pathways. Achieving efficient delivery of these biomacromolecules requires overcoming the challenge of cellular uptake, particularly for large, hydrophilic proteins.^[^ ^1^, ^2^, ^3^ ^]^ One promising strategy to enhance cell permeability involves the incorporation of arginine residues, as often found in cell‐penetrating peptides (CPPs).^[^ ^4^ ^]^ Alongside induction of endosomal uptake and escape, direct translocation of proteins has been demonstrated to be an effective approach to deliver functional proteins.^[^ ^5^, ^6^, ^7^, ^8^, ^9^ ^]^

Several strategies have been developed for incorporating CPPs into proteins.^[^ ^10^ ^]^ These approaches include genetic fusion of CPPs with the protein of interest, semi‐synthetic routes (e.g., intein‐mediated ligation, sortase, etc.), or conjugation using selective chemical handles, such as unnatural amino acids or cysteine residues.^[^ ^11^, ^12^, ^13^, ^14^ ^]^ Notably, semi‐synthetic and conjugation‐based strategies offer significant advantages for the incorporation of diverse CPP modalities, such as cyclic peptides and unnatural amino acid building blocks. Moreover, they allow the installation of cleavable linkages, commonly via reducible disulfides, to ensure the removal of the CPP within the cytoplasm following cellular entry to avoid unwanted intracellular accumulation.^[^ ^15^ ^]^ Despite their utility, many of the aforementioned concepts have significant limitations. In particular, the genetic or chemical engineering of CPP‐protein conjugates is laborious and may alter the native structure and function of the protein cargo. In parallel, the attachment of several linear or cyclic CPPs has been explored to lower the required concentration to allow cellular uptake.^[^ ^14^, ^16^, ^17^, ^18^ ^]^ While attaching multiple CPPs may enhance cellular uptake, complex syntheses or conjugation schemes as well as the often‐overlooked increased toxicity due to excessive positive charges prevent general application.^[^ ^19^ ^]^

Recently, our group has introduced cell‐surface‐anchored CPP‐additives, which significantly lower the critical concentration required to achieve direct translocation of homogeneous CPP‐cargo‐conjugates, thereby allowing the delivery of functional proteins and antibodies even for endogenous targeting.^[^ ^20^, ^21^ ^]^ Nevertheless, a broadly applicable and accessible delivery platform for native proteins that avoids toxicity and the need for genetic modification remains elusive.

**Figure 1:**
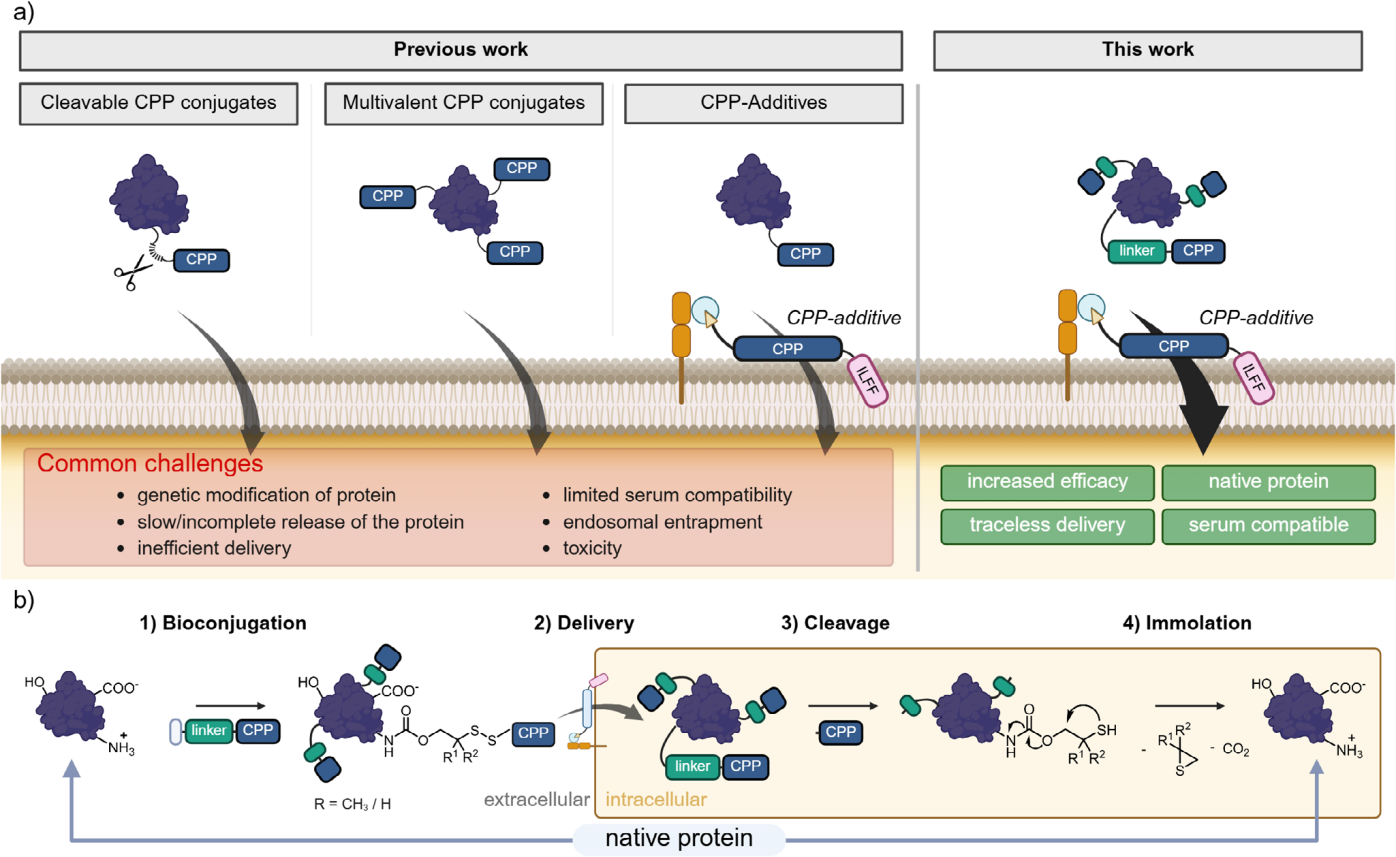
a) Selection of previous approaches using CPP‐conjugates for protein delivery in comparison to this work. Advantages and challenges are listed in green and red, respectively. CPP = oligoarginine peptide, mostly R_8–10_ (cyclic and linear); for the structure of the CPP‐additive, see Figure S1 and Arafiles et al, *JACS* 2023.^[^ ^21^ ^]^ b) Concept of Bioreversible Arginine Modification (BioRAM) for protein delivery following the steps: selective bioconjugation on aliphatic amines (lysine side chains and *N*‐terminus) (1), cellular delivery (2), cleavage of the CPP from protein by reduction of the disulfide bond (3), and traceless immolation of the residual linker back to the native protein (4).

Here, we aim to address this gap by introducing a novel protein delivery strategy that combines the strengths of several of the previously mentioned concepts, namely multiple CPP‐attachments to a protein cargo, traceless intracellular CPP‐cleavage yielding the native protein, and cell‐surface anchored CPP‐additives with straightforward CPP‐conjugation to a genetically non‐engineered protein cargo (Figure 1). Our strategy, called **BioRAM** (**BioR**eversible **A**rginine **M**odification), enables the attachment of CPPs to the protein surface via intracellularly cleavable linkers using amine‐selective conjugation (*N*‐terminus, lysines). BioRAM generates heterogeneously CPP‐modified proteins. Upon cytosolic delivery, the CPPs are cleaved‐off, recovering the native protein and preventing unwanted intracellular localization. We meticulously probed and compared the efficacy and the toxicity of attaching several shorter CPPs vs. fewer longer CPPs, thus providing insights into the optimal condition for delivery. Finally, key to our linker design was to balance extracellular stability and rapid intracellular cleavage, which ensured direct translocation with immediate cytosolic availability, thus offering a serum‐compatible and non‐toxic, versatile, native protein delivery platform for intracellular targeting.

## Results and Discussion

### Design of Self‐Immolative Amine Reactive Linker

**Figure 2:**
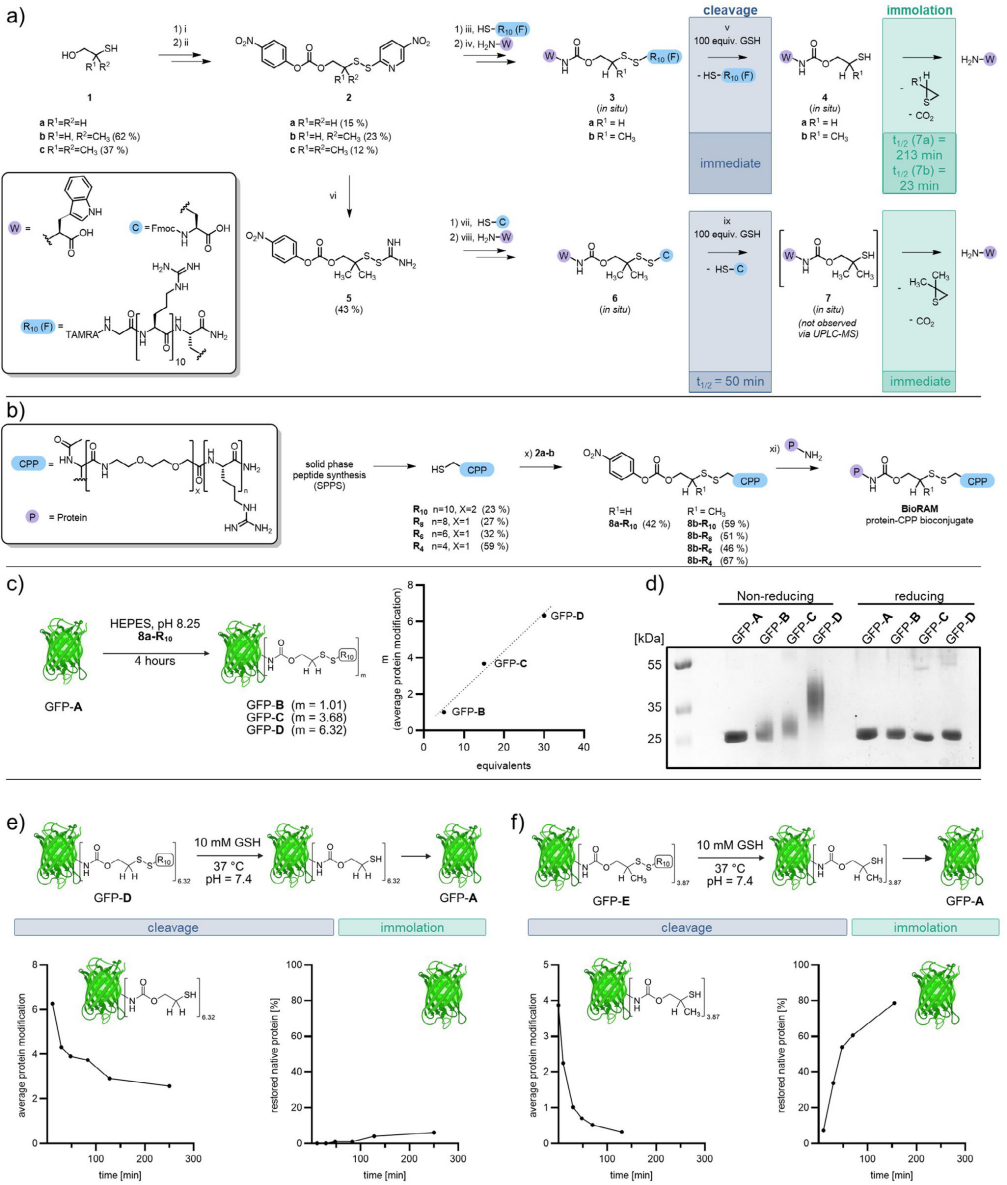
a) Synthesis of disulfide linker with α‐substitution. Compound 1a is commercially available; compounds 2b‐c were synthesized following published procedures (Figure S2). i) 2,2′‐dithiobis(5‐nitropyridine), DCM, MeOH, 20 °C, 24 h; ii) 4‐nitrophenyl chloroformate, Et_3_N, MeCN, 0→20 °C, 24 h; iii) H_2_O, pH 4.35, 20 °C, 16 h; iv) H_2_O, pH 8.25, 20 °C, 16 h; v) H_2_O, pH 7.4, 37 °C. Reduction and immolation of 3a‐b (0.1 mmol) is monitored using analytical HPLC (Figure S5). Half‐lives (t_1/2_) based on integrated peak intensity at 220 or 280 nm. Dimethylated disulfides 2c were unreactive towards thiols. The respective mixed disulfide 6 could only be accessed via 5, using thiourea as the activating group. vi) thiourea, formamidine, DMF, 20 °C, 1 h. vii) MeCN/H_2_O (1:1), pH 8.25, 20 °C, 30 min; viii) MeCN/H_2_O (1:1), pH 8.25, 20 °C, 16 h; ix) H_2_O, pH 7.4, 20 °C. Half‐lives based on integrated peak intensity of starting material measured by UPLC‐MS (Figure S4). b) Synthesis of polyarginine peptide R_4_‐R_10_ followed by selective disulfide exchange at pH 4.35 yielding 8a/b‐R_n_, which can be further reacted with native protein to produce BioRAM bioconjugates. x) MeCN/H₂O (1:1), pH 4.35, 20 °C, 2 h; xi) H₂O (1:1), pH 8.25, 20 °C, 4 h. c) Bioconjugation of superfolded 50 µM GFP (a) with 5–30 equivalents of 8a‐R_10_ in 200 mM HEPES yielding GFPs B‐D. QTof HR‐MS analysis of GFPs (b‐d) indicated that an increase in equivalents of 8a‐R_10_ resulted in a higher average modification of the GFPs, showing a linear correlation at the concentrations tested. d) Reducing and non‐reducing SDS‐PAGE gel analysis of GFPs A‐D reacted with different equivalents of 8a‐R_10_. e) Reduction and immolation of 1 µM sfGFP D (reacted with 8a‐R_10_) upon treatment with 10 mM GSH. f) Reduction and immolation of 1 µM sfGFP E ‐8b‐R_10_ upon treatment with 10 mM GSH.

To develop a bioconjugation strategy that could be easily applied to native proteins, we employed a carbonate‐based linker to target abundant and solvent‐exposed lysine side chains and *N*‐terminal primary amines.^[^ ^22^ ^]^ By probing different α‐substituted disulfides,^[^ ^23^ ^]^ we aimed to identify an optimal linker with a fast immolation cascade and high (extracellular) stability. Previously, He et al. used unsubstituted disulfides in linking PEG, DNA, or CPPs to proteins,^[^ ^24^ ^]^ however, the stability of the protein‐CPP conjugate was not investigated. Researchers at Genentech previously demonstrated the use of α‐substituted disulfides for conjugating drugs to engineered antibodies with free thiols, creating antibody‐drug conjugates (ADCs).^[^ ^25^ ^]^ Their studies revealed that dimethyl‐substituted disulfides exhibit superior serum stability and enable rapid immolation to release the free drug. Nevertheless, they also reported that the increased steric hindrance also diminished intracellular disulfide cleavage. Consequently, we started our investigations by synthesizing amine‐reactive α‐substituted disulfide linkers for protein‐CPP conjugations, employing non‐, mono‐, and dimethylated disulfides in linkers **2a–c** (Figures 2a and S2).^[^ ^26^, ^27^ ^]^

To evaluate the reactivity of the 5‐nitropyridine‐2‐thiol group for **2a–c**, we performed test reactions with 1 equiv. cysteine. (Figure S3). We observed selective conversion to the desired disulfide at pH 4.35 for **2a** and **2b**, which ensures stability of the carbonate throughout the entire reaction.^[^ ^28^ ^]^ Dimethylated compound **2c** did not convert to the desired mixed disulfide even at pH 7.4 and 140 equiv. of cysteine. Instead, changing the activating group to thiourea in **5** enabled disulfide formation with Fmoc‐protected cysteine,^[^ ^25^ ^]^ whereas incubation with tryptophan as a model cargo with a free primary amino group and high absorption at 280 nm yielded mixed disulfide **6**. Stability studies under reducing conditions analogous to the intracellular environment (0.1 mmol compound **6**, 10 mM reduced glutathione (GSH) at pH 7.4, 20 °C)^[^ ^29^ ^]^ revealed slow cleavage of the disulfide (half‐life (t_1/2_) = 50 min, Figure S4). Because of accumulation of cationic CPPs in nucleolar compartments, our later applications require rapid liberation of the protein.^[^ ^30^ ^]^ Therefore, we consider α‐dimethylated‐disulfide linkers unsuitable for our purpose as we would expect significant unwanted intracellular localization.

Next, we reacted unmethylated **2a** and α‐monomethylated **2b** with a fluorescent cysteine containing CPP (R_10_) under acidic conditions at pH 4.35 to generate the corresponding carbonates (Figure 2a). Further incubation with unprotected tryptophan resulted in quantitative formation of **3a** and **3b**. These mixed disulfides (0.1 mmol) were incubated with GSH (10 and 100 equiv., pH 7.4, 37 °C), and the disulfide cleavage and immolation via sulfide **4a** or **4b** were monitored over time by HPLC (Figures 2a and S5). Although the non‐methylated disulfide **3a** was rapidly cleaved (complete cleavage <6 min), the intermediate **4a** immolated incompletely (*t*_1/2_ > 200 min). The observed by‐product was the mixed GSH‐disulfide that prevented the release of the free amine. The fluorescent derivative **3b** was more stable at a low excess of GSH (10 equiv.) and immediately cleaved at higher GSH concentration (100 equiv., complete cleavage <8 min). Furthermore, intermediate **4b** showed complete immolation under physiological conditions with *t*_1/2_ = 23 min (Figures 2a and S5). We rationalize that the α‐methylation stabilizes the disulfide against disulfide exchange at low thiol concentrations and increases cyclization of the cleaved intermediate through the Thorpe–Ingold effect (Figure S6).^[^ ^31^, ^32^ ^]^

### CPP‐bioconjugation to Proteins

Next, we used the non‐ and mono‐methylated disulfide carbonates **8a‐R_10_** and **8b‐R_10_** in bioconjugation reactions with solvent exposed aliphatic amines on superfolded green fluorescent protein (sfGFP) as a model protein (Figure 2c). We observed the best performance at pH 8.25 (20 °C for 4 h). To determine and characterize the exact number of peptide modifications on the protein, we performed electrospray ionization (ESI) on a quadrupole time‐of‐flight high‐resolution mass spectrometer (QTof HR‐MS) (Figure S7). We observed a good correlation between the equivalents of applied peptide and the number of modifications on the protein after the reaction (Figure 2c). The degree of modification is referred to as average protein modification (apm), which was calculated based on the peak intensity of the native MS spectra (Figure S8). Additionally, protein‐CPP conjugation was easily visualized by non‐reducing sodium dodecyl sulfate‐polyacrylamide gel electrophoresis (SDS‐PAGE) analysis (Figure 2d). The attachment of peptides led to an increase in molecular weight, resulting in a band shift toward higher molecular weights. The observed band smearing is attributed to the heterogeneity of the protein mixture and the absence of a reducing agent, which typically produces sharper, more defined bands. Adding a reducing agent (β‐mercaptoethanol) cleaved the peptides from the protein, resulting in a protein band on the gel that corresponds to the unmodified protein.

To test the immolation and recovery of the native protein under conditions resembling the intracellular environment, 1 µM GFP‐**D** and GFP‐**E** were incubated with 10 mM GSH and monitored by QTof HR‐MS (Figure 2e,f). For both GFP‐**D** and GFP‐**E**, disulfides were completely reduced 3 min after adding GSH. The first reaction step—cleavage of the CPP from the protein—proceeded at a similar rate, independent from the methylation. By monitoring the residual modification on the protein, we infer the progress of the second step of the reaction, the rate of immolation. GFP**‐D** with a non‐methylated disulfide remained only partially immolated even after 250 min (Figures 2e and S9A–F). In contrast, for GFP‐**E** with mono‐methylated disulfide, half of the immolation took place within the first 20 min (Figures 2f and S9G–J). These results confirm the observations on the fluorescent model peptides **3a‐b**. Fast and complete immolation towards the native protein is only achieved by the methylated disulfide derivative. Hence, we proceeded with the mono‐methylated disulfide linker for succeeding conjugations that will from hereon be referred to as BioRAM‐proteins or ‐conjugates.

For delivery experiments in living cells, we utilized the red fluorescent protein mCherry fused with a nuclear localization signal (NLS) as the model protein cargo (NLS‐mCh‐**A**).^[^ ^15^, ^20^, ^21^ ^]^ The NLS sequence directs the protein to the cell nucleus, enabling us to use nuclear localization as a basis for subsequent quantification (for code and detailed explanation, refer to the Supporting Information “*Intranuclear fluorescence intensity measurement”* section). BioRAM allows for multiple modifications on a protein. To understand how the number of arginines per modification affects protein properties, we tested different polyarginine lengths. In addition to **8b‐R_10_**, we conjugated shortened polyarginines CPPs **8b‐R_8_**, **8b‐R_6_**, and **8b‐R_4_** to NLS‐mCh‐**A**, yielding NLS‐mCherry‐BioRAM conjugates NLS‐mCh‐**B**, ‐**C**, ‐**D**, and ‐**E** (Figure 2b). To allow direct comparison, we aimed to maintain a consistent overall charge of each NLS‐mCherry‐BioRAM conjugate. In our calculations, we considered that each modification is attached to a lysine, which reduces the protein's surface charge by one, while each added arginine increases the charge by one (Figure S8). As a result, NLS‐mCherry‐BioRAM conjugates with shorter polyarginines require a higher degree of modification to produce a protein bioconjugate with a similar positive charge.

**Figure 3:**
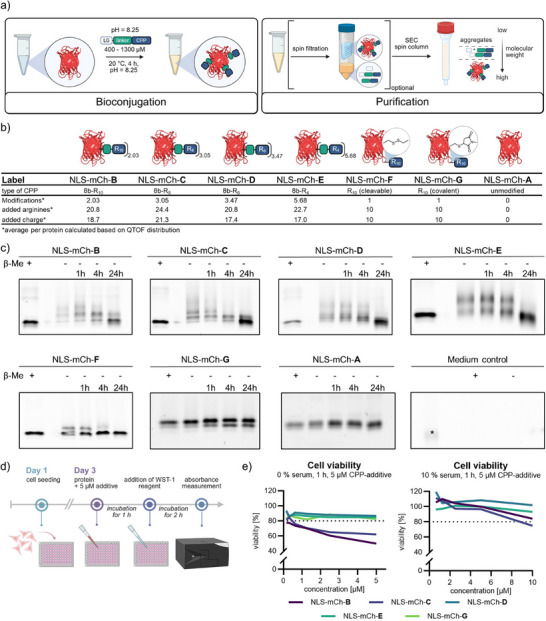
a) General workflow for the modification and purification using BioRAM. b) apm calculated from QTof‐HR‐MS analysis (refer to Figure S8). Added arginine residues are calculated based on the peptide length multiplied by the average modification. The calculated added charge considers the modification of one free lysine (positively charged) for the attachment of one polyarginine. c) Fluorescent SDS‐PAGE gel analysis of NLS‐mCherry‐BioRAM in DMEM‐high glucose medium supplemented with 10% FCS over 1, 4, and 24 h. The fluorescent 25 kDa band of the ladder is marked (*) d) Schematic treatment protocol for cell viability assessment. e) Cell viability assay using WST‐1 for protein treatment at 0% serum (left) and 10% serum (right) in the presence of 5 µM CPP‐additive. Data is represented as the mean of three biological replicates (*N* = 3).

The modification with the lysine‐reactive polyarginines was done using 2.5–8.3 equivalents of the respective peptide‐carbonates **8b** at pH 8.25 for 4 h. The proteins were purified using a combination of Amicon concentration (molecular weight cut‐off [MWCO] of 10 kDa) followed by Zeba spin purification (MWCO 7 kDa) (Figure 3a) to concentrate the sample and remove excess peptide as well as potential protein aggregates. Native MS analysis showed an average modification ranging from 2.0–5.8 modifications per protein, which was in good accordance with the SDS‐PAGE gel profile (Figure S10). In addition, the genetically fused *C*‐terminal GGC amino acid sequence was used for the site‐selective attachment of a single cleavable (i.e., disulfide‐linked, NLS‐mCh‐**F**) and non‐cleavable (i.e., maleimide‐linked NLS‐mCh‐**G**) R_10_ ‐modification as controls following previously published procedures (Figure 3b).^[^ ^15^ ^]^

### Cellular Application Using Fluorescent Protein

First, we assessed the serum stability of BioRAM‐proteins by fluorescent SDS‐PAGE gel analysis. We incubated proteins NLS‐mCh‐**A** to ‐**G** for 1, 4, and 24 h in cell culture medium (Dulbecco's Modified Eagle's Medium‐high glucose, DMEM‐HG) supplemented with 10% fetal calf serum (FCS). The samples were prepared by dilution in Laemmli buffer without a reducing agent and heating. As anticipated, the non‐cleavable maleimide control NLS‐mCh‐**G** remains stable over the course of the experiment. While other protein‐CPP conjugates are mostly cleaved after 24 h, all BioRAM conjugates retained their modifications for at least 4 h of incubation (Figures 3c and S11). This result suggests sufficient stability for cellular assays in the presence of serum.

In further experiments, the cellular delivery properties of BioRAM conjugates were tested in combination with the CPP‐additive (**TNB‐R_10_‐ILFF**), which is prepared and added separately using a 1 mM DMSO stock. The CPP‐additive consists of a decaarginine, an *N*‐terminal thiol reactive 5‐thio‐2‐nitrobenzoic acid disulfide (TNB) that is able to react with cell surface thiols, and a *C*‐terminal hydrophobic peptide tag (ILFF), which prolongs membrane retention by hydrophobic interactions (Figure S1).^[^ ^21^ ^]^

Next, we focused on evaluating the cytotoxicity of BioRAM‐conjugates. High concentrations of polyarginines, especially multivalent constructs, are known to cause cytotoxicity by unspecific membrane rupture.^[^ ^19^ ^]^ Therefore, we carefully monitored cell viability and checked acute toxicity after 1 h of incubation of the proteins NLS‐mCh‐**A** to ‐**G** in the presence of 5 µM CPP‐additive (**TNB‐R_10_‐ILFF**) (Figure 3d). After 1 h, tetrazolium salt (WST‐1) was added to all wells to evaluate metabolically active cells. Formation of the formazan dye through the mitochondrial succinate‐tetrazolium‐reductase system can be monitored by absorption at 460 nm and directly correlated to cell viability. Concentrations greater than 1 µM of NLS‐mCh‐**B** (modified with **8b‐R_10_**, 50% viability at 5 µM, no serum) as well as NLS‐mCh‐**C** (modified with **8b‐R_8_**, 62% at 5 µM, no serum) in the absence of serum showed significant toxicity, while the homogenous conjugate with site‐selective attachment of one R_10_ (NLS‐mCh‐**G**) was non‐toxic under the tested concentrations. Multivalent conjugates with shorter polyarginines, NLS‐mCh‐**D** (modified with **8b‐R_6_**) and NLS‐mCh‐**E** (modified with **8b‐R_4_**), showed no toxicity for all conditions tested (up to 5 µM, no serum) (Figure 3e). The addition of 10% serum during the incubation ensured viability (>80 %) of cells treated with up to 5 µM of modified protein. Previous works report that the presence of serum decreases the toxicity of polycationic CPPs due to unspecific adsorption with serum proteins.^[^ ^33^, ^34^ ^]^

**Figure 4:**
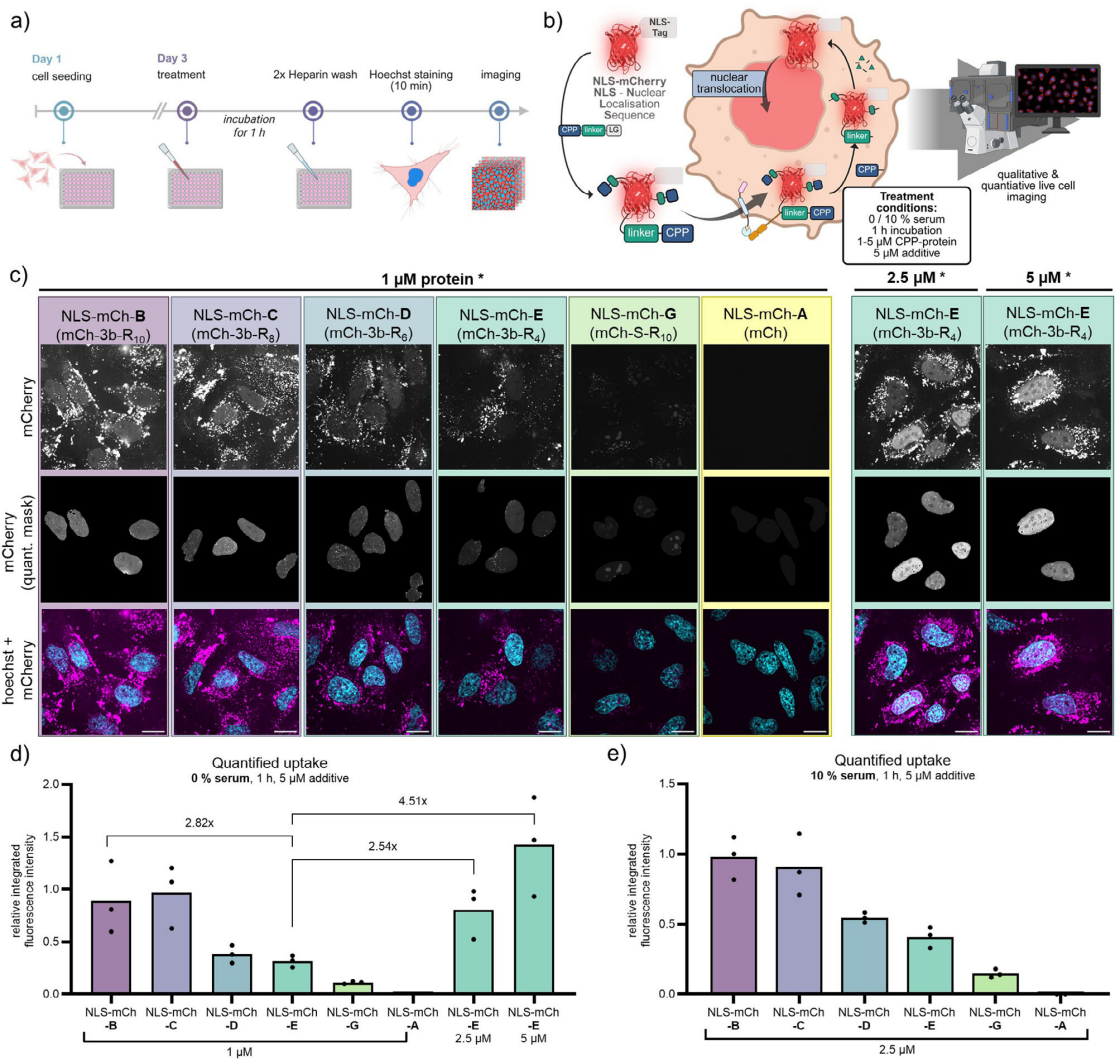
a) Schematic workflow assessing the cellular uptake of fluorescent BioRAM‐proteins into cells. b) Visualization of uptake, nuclear translocation, and colocalization to the nucleus. c) Representative confocal microscopy images from the automated acquisition during quantification measurements in a 96‐well plate using HeLa cells (Nikon 40× Air). Microscope settings and image contrast are kept constant for all images. RFP‐mask was generated using nuclear segmentation and endosome exclusion script (Figure S12B). Scale bar = 20 µm. (*incubation for 1 h, 5 µM CPP‐additive, 0% serum) d), e) Quantification results displaying integrated relative fluorescence intensity. Data are reported as the mean of three biological replicates (*N* = 3, black line), with each biological replicate (dot) representing the mean of 150–200 individual cells. Fold change of relative integrated fluorescence intensity is displayed for selected conditions.

For protein delivery, HeLa cells were seeded into 96 well plates until 70%–90% confluency. After washing the cells once with phosphate buffered saline (PBS), **TNB‐R_10_‐ILFF** additive (5 µM) was added together with proteins NLS‐mCh‐**B**, ‐**C**, ‐**D**, ‐**E,** and ‐**G** (1 µM) in serum‐free DMEM‐HG medium and incubated for 1 h (Figure 4a). After washing with heparin to remove excess extracellular protein, treated cells were imaged by live‐cell confocal microscopy. The images showed a significant increase in uptake for all BioRAM‐proteins compared to the control protein NLS‐mCh‐**G**, which contained one site‐selectively attached R_10_ (Figure 4c). Whereas endosomes are observed as bright dots across all microscopy images, delivered fluorescent proteins in the cytosol are immediately translocated to the nucleus due to the NLS‐sequence. As a result, protein appears as a dispersed signal within the nuclear compartment. The covalently connected polyarginine in the case of NLS‐mCh‐**G** led to a distinct signal in the nucleoli, an RNA‐rich, membraneless compartment within the nucleus (Figure 4c, NLS‐mCh‐**G**) as reported previously.^[^ ^15^ ^]^ The absence of this distinct nucleolar fluorescence in the case of the cleavable variants NLS‐mCh‐**B** to ‐**E** compared to maleimide conjugated NLS‐mCh‐**G** supports the successful cleavage of the CPPs from the protein (mCherry quantification mask in Figure 4c). For quantification we utilized the colocalization of the NLS‐mCherry signal with a DNA stain (i.e., Hoechst channel) (Figure 4b,c).^[^ ^21^ ^]^ A previously published script was further improved to enable simple cell segmentation that allows us to run an automated quantification image analysis, which excludes most of the dominant signal from endosomes (for code and detailed explanation, refer to the Supporting Information “*Intranuclear fluorescence intensity measurement”* section). Quantification of dispersed nuclear fluorescence was performed by measuring the median fluorescence intensity inside the nucleus as exemplarily shown in Figure S12B. Automated analysis was conducted on 200 cells per well per biological replicate (total of three biological replicates).

Our analysis determined NLS‐mCh‐**B** (modified with **8b‐R_10_**) and NLS‐mCh‐**C** (modified with **8b‐R_8_**) as conjugates with the highest cytosolic uptake, followed by NLS‐mCh‐**D** (modified with **8b‐R_6_**) and NLS‐mCh‐**E** (modified with **8b‐R_4_**) (Figure 4d). Although we see decreasing signal intensity in the nucleus for shorter polyarginines, all heterogeneously modified constructs showed significantly improved direct translocation compared to the site selectively, single conjugated NLS‐mCh‐**G**. The decreased delivery efficacy of shorter CPPs was fully compensated by using higher concentrations of the protein (Figure 4d). These results indicate that multiple CPPs with shorter arginine sequences are capable of efficiently delivering protein into the cells when conjugated to the protein cargo without compromising cell viability.

Intrigued by the previously observed stability in serum and the high delivery efficacy of the bioconjugates NLS‐mCh‐**B** to ‐**E,** we subsequently assessed cell uptake in the presence of serum. The addition of serum leads to unspecific interactions of cationic CPPs with serum proteins. CPP‐serum protein binding reduces the amount of free CPPs that engage with cells, leading to reduced cytotoxicity but also decreasing the fraction of cytosolically delivered peptide.^[^ ^33^, ^34^ ^]^ Hence, most cationic CPPs relying on direct translocation are rendered serum incompatible. HeLa cells were incubated with 2.5 µM protein and 5 µM CPP‐additives for 1 h in growth medium containing 10% FCS. Cellular uptake was assessed qualitatively using the same assay as stated before (Figure S12). Surprisingly, we observe dispersed nuclear fluorescence for all NLS‐mCherry‐BioRAM conjugates, having an increased performance compared to the control NLS‐mCh‐**G** with only one site selective, covalent R_10_. Upon quantification, the trend of NLS‐mCh‐**B** being the most efficient conjugate, followed by NLS‐mCh‐**C**, ‐**D**, and ‐**E,** respectively, remained similar for both serum conditions (Figure 4e).

**Figure 5:**
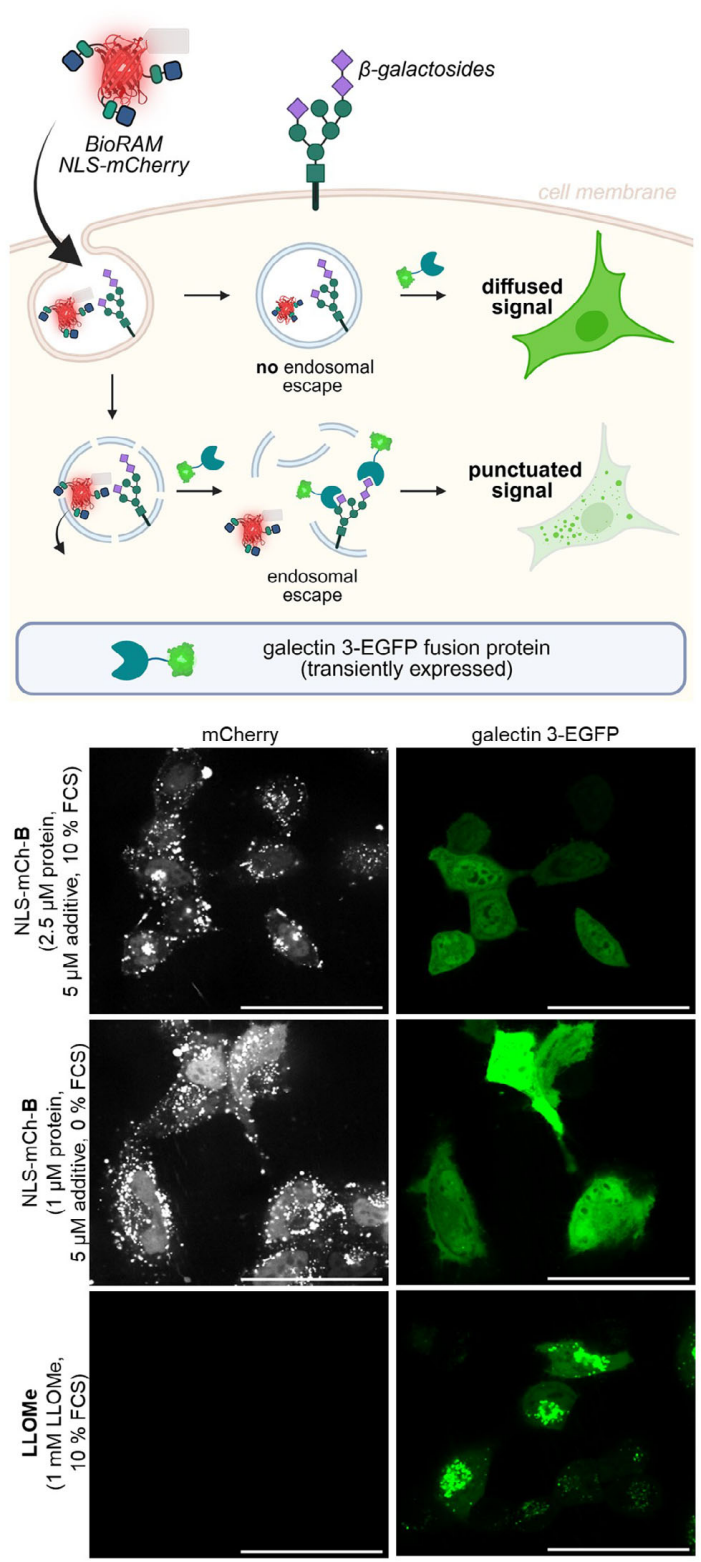
Transiently transfected HeLa cells expressing cytosolic galectin 3‐EGFP fusion protein for detection of endosomal escape. Nuclear mCherry signal indicates successful cytosolic delivery. Punctuated EGFP‐signal is indicative of endosomal rupture as induced by the positive control LLOMe. Microscopy images (Nikon 60× oil), scale bar = 50 µM.

Although the CPP additives were specifically designed to promote direct translocation as the primary mode of protein uptake, we considered the possible contribution of endosomal escape to the observed cytosolic signal. To investigate this, we transiently transfected HeLa cells with a plasmid that allows the expression of galectin‐3 fused to enhanced green fluorescent protein (gal3‐EGFP) in the cytosol.^[^ ^35^ ^]^ Endosomal membrane rupture would expose the endosomal lumen, allowing galectin‐3 to bind β‐galactosides, which are typically located on the luminal side of endosomal membranes, resulting in distinct fluorescent puncta from the binding and accumulation of gal3‐EGFP fusion protein to ruptured endosomes (Figure 5).^[^ ^36^ ^]^ We observed such fluorescent puncta when cells are treated with L‐leucyl‐L‐leucyl methyl ester (LLOMe), a dipeptide known to destabilize endosomes.^[^ ^37^ ^]^ In contrast, NLS‐mCh‐**B**‐treated cells showed no punctate signals in fluorescence microscopy images, while a clear nuclear mCherry signal confirmed successful cytosolic delivery (Figure 5). These findings strongly suggest that protein uptake using the combined BioRAM‐CPP additive technologies occurs predominantly through direct translocation, with no immediately observable contribution from endosomal escape.

### Functional RNase Delivery at Nanomolar Concentrations

**Figure 6:**
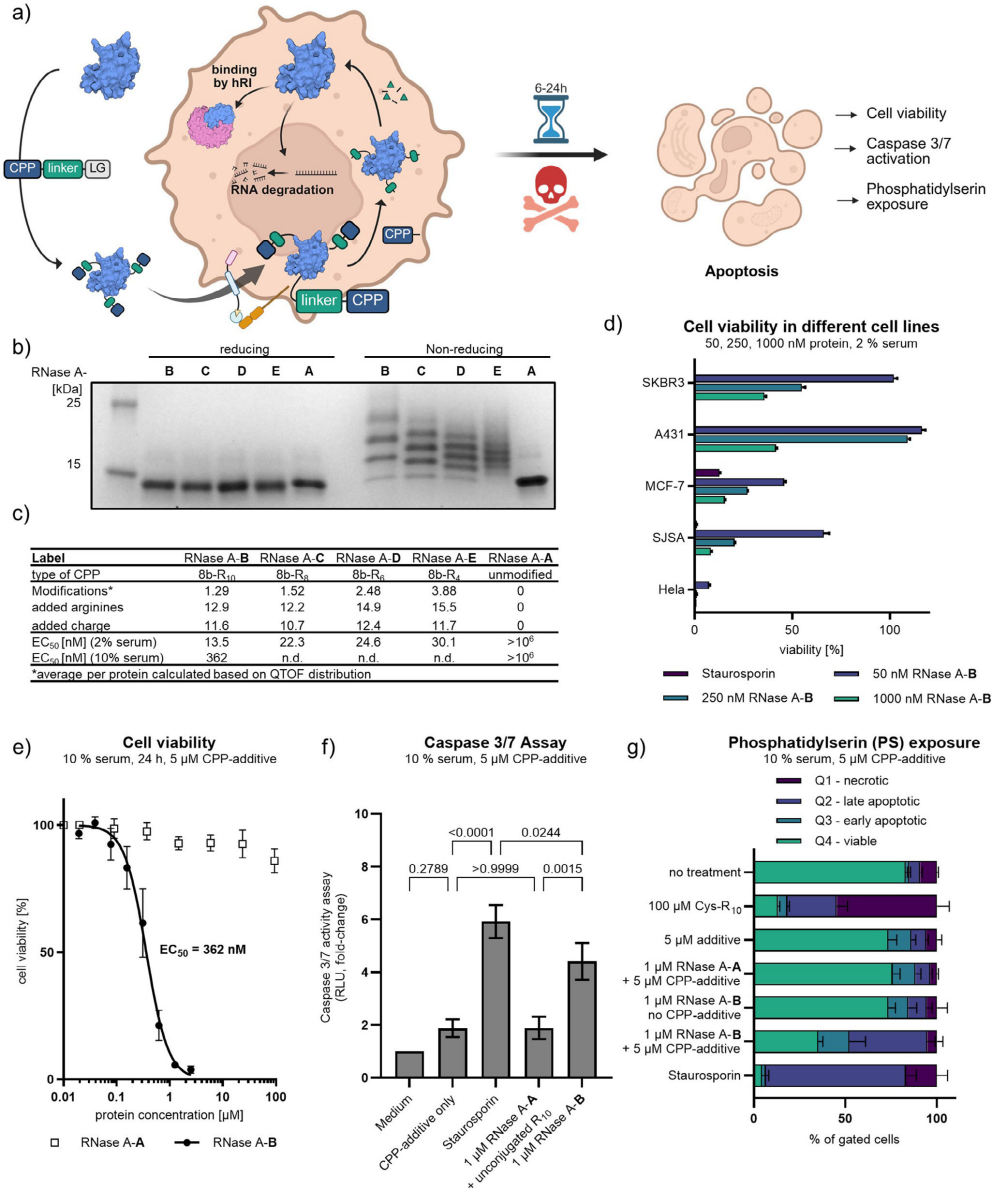
a) Schematic representation of RNase A‐BioRAM delivery and intracellular function. b) SDS‐PAGE gel of generated RNase A‐BioRAM conjugates. c) Characterization of generated RNase A‐BioRAM conjugates and results of cell viability assays after treatment of protein in presence of 5 µM CPP‐additive. (The data is presented as the mean of three replicates (*N* = 3)). d) Cell viability in different cell lines after treatment with the indicated concentrations of RNase A‐B in the presence of 5 µM CPP‐additive. (The data is presented as the mean ± SD of three replicates (*N* = 3)). e) Cell viability of HeLa cells after 24 h of treatment with RNase A‐B compared to RNase A‐A in the presence of 5 µM CPP‐additive (The data is presented as the mean ± SD of three biological replicates (*N* = 3)). f) Activation of caspase‐3/7 was measured using a luminogenic caspase‐3/7 substrate producing a luminescent signal by luciferase after caspase activation. Supernatant was analyzed with a plate reader. (The data is presented as the mean ± SD of three biological replicates (*N* = 3). The indicated P‐values are calculated using one‐way ANOVA followed by Tukey's posthoc analysis). g) PS exposure was assessed using fluorochrome‐conjugated Annexin V, which binds specifically to extracellularly exposed PS on the cell membrane. Stained cells were analyzed by flow cytometry to quantify apoptotic populations based on fluorescence intensity (the data is presented as mean ± SD of three biological replicates (*N* = 3)). Representative pictures of cell populations can be found in Figure S17.

With the possibility of modifying and efficiently delivering native proteins in the presence of serum, we utilized the BioRAM method for the delivery of a functional protein. Ribonuclease A (RNase A) is a 13.7 kDa enzyme isolated from bovine pancreas with high homology to the human homolog.^[^ ^38^ ^]^ This enzyme catalyzes the degradation of free RNAs through the hydrolysis of the P─O^5´^ bond.^[^ ^39^, ^40^ ^]^ Delivery of exogenous RNase A into cells leads to large‐scale catalytic degradation of free RNA, thereby inhibiting protein synthesis and leading to the induction of apoptotic cell death (Figure 6a).^[^ ^41^, ^42^ ^]^ Extensive investigation of the RNase A superfamily for medical treatment has been translated to clinical candidates against cancer.^[^ ^43^ ^]^

We modified bovine RNase A (RNase A‐**A**) with compounds **8b‐R_10_**, **8b‐R_8_**, **8b‐R_6_**, and **8b‐R_4_** to yield BioRAM‐RNase A (RNase A‐**B** to ‐**E**, respectively) with different conjugated polyarginine peptide lengths (Figures 6b,c, and S13). As discussed above, we attempted to keep the overall charge of the BioRAM‐RNase A conjugates similar across the derivatives by increasing the degree of modification for shorter polyarginines. BioRAM–RNase A conjugates showed just slightly reduced activity in in vitro RNase activity assays relative to the unmodified enzyme, independent of polyarginine length or modification extent (Figure S14). This indicates that protein structure remains intact. The decrease likely stems from electrostatic interference between RNA and the CPPs, which are cleaved intracellularly—therefore, intracellular activity is not expected to be affected. The conjugates were first tested under reduced serum containing conditions (2% FCS in minimal essential medium (Opti‐MEM)) in the presence of 5 µM CPP‐additive (**TNB‐R_10_‐ILFF** ^[^ ^21^ ^]^). Successful cellular delivery was confirmed by assessing cell viability using the WST‐1 assay after 24 h of treatment. We determined a half effective concentration (EC_50_) of 13.5 nM for RNase A‐**B **(modified with **8b‐R_10_**) (Figures 6c and S15). The BioRAM‐RNase A‐**C** to ‐**E** with reduced polyarginine length were only slightly less efficient (EC_50 _= 13.5–30.1 nM) (Figures 6C and S15), while a significantly lower cell death was observed in the absence of the CPP‐additive (**TNB‐R_10_‐ILFF**) (Figure S16). The fold‐difference of activity between RNase A‐**B **(modified with **8b‐R_10_**) and RNase A‐**E **(modified with **8b‐R_4_**) is calculated to be 2.59× (Figure S15), which is similar to the corresponding 2.82× fold‐difference of NLS‐mCherry uptake using the respective BioRAM conjugate in Figure 4e. Furthermore, the effect of the most potent derivative, RNase A‐**B** was investigated with several human cell lines. Concentrations higher than 1 µM of RNase A‐**B** resulted in cell death in all cell lines (Figure 6d). Differences in EC_50_ among cell lines may stem from variations in cellular response to RNase A or from different delivery efficiencies.

To challenge the delivery method under serum conditions, the cytotoxicity of RNase A‐**B** was assessed with HeLa cells using the standard growth medium containing 10% FCS over 24 h in the presence of 5 µM CPP‐additive (**TNB‐R_10_‐ILFF**). The EC_50_ value was determined to be 362 nM after 24 h of incubation (Figure 6e). Although this experiment showed an increase in EC_50_ value, our BioRAM‐modified RNase A remains a potent method to induce cell death in comparison with previously reported RNase A delivery methods. Liu et al. showed effective delivery into HeLa cells (EC_50 _= 1–4 µM) using cationic oligomers.^[^ ^44^ ^]^ Others have shown delivery in MCF‐7 cells by permanently cationized RNase^[^ ^45^ ^]^ (EC_50_ = 85 nM), lipid nanoparticles^[^ ^46^ ^]^ (EC_50 _= 2–4 µg mL^−1^ ∼0.150–0.300 µM) or DNA‐origami^[^ ^47^ ^]^ (EC_50 _= 2−4 µg mL^−1^ ∼0.150–0.300 µM). To verify that cell death was due to apoptosis rather than unselective membrane rupture (necrosis) by multivalent polyarginines, we assayed for the activation of executioner caspases‐3/7 (Figure 6f) and extracellular exposure of phosphatidylserine (PS) (Figures 6g and S17). Both assays confirmed the induction of apoptosis by RNase A‐**B** in the presence of CPP‐additive (**TNB‐R_10_‐ILFF**).^[^ ^48^ ^]^

Human‐derived cell lines endogenously express human ribonuclease inhibitor protein (hRI), which tightly binds to members of RNase A (*K*_i_ = 4.4 × 10^−14^).^[^ ^49^ ^]^ Whereas hRI's full biological function and potential applications in protein engineering remain areas of active investigation, it is known that hRI protects mammalian cells from extracellular ribonucleases.^[^ ^50^ ^]^ Due to its high binding affinity, any exogenous RNase A that reaches the cytosol is rapidly bound by hRI, effectively neutralizing its enzymatic activity. Therefore, to induce apoptosis via RNase activity, the cellular amount of endogenous hRI must first be surpassed. The cytosolic concentration of hRI is consistent across different cell lines and has been estimated at approximately 4 µM.^[^ ^51^ ^]^ The most potent derivative, RNase A‐**B**, achieves an EC_50_ of 13.5 nM (2% serum)—approximately 300 times lower than the estimated intracellular concentration of hRI. This discrepancy suggests that RNase A molecules accumulate within cells over time, surpassing the hRI threshold. Additionally, previous studies have demonstrated the intracellular delivery of various engineered RNase analogs exhibiting potent cytotoxicity, particularly mutated variants designed to evade hRI binding, exhibiting potent cytotoxicity.^[^ ^52^ ^]^ Consequently, RNases known or designed to evade hRI binding could exhibit even greater cytotoxicity and warrant further testing using this delivery method.

Our results support that BioRAM enables multiple conjugations of CPPs to the protein in water, resulting in significantly enhanced cellular uptake for all NLS‐mCherry‐BioRAM (NLS‐mCh‐**B** to ‐**E**) and RNaseA‐BioRAM conjugates (RNase A‐**B** to ‐**E**). Other bioreversible CPP‐bioconjugates utilize carbonates and esters,^[^ ^14^ ^]^ which require the addition of organic co‐solvents. In addition, the disulfide‐BioRAM‐linker is cleaved independent of enzymes (e.g., esterases^[^ ^14^ ^]^) and their associated kinetics, offering more predictable release and transferability across different cell types. We generated BioRAM conjugates with comparable surface charges and found that delivery efficacy decreased with shorter polyarginine length. While longer polyarginine derivatives (BioRAM‐R_10_ and BioRAM‐R_8_) showed higher delivery efficacy, they also showed slight cytotoxicity similar to previous reports.^[^ ^19^ ^]^ The addition of serum reduced toxicity across all conjugates while still allowing protein delivery. Notably, even BioRAM‐R_4_ enabled cytosolic delivery when used in multivalent conjugates. Shorter polyarginines (<R_8_) have been tested as single conjugated CPPs but showed very poor membrane penetration compared to longer polyarginine chains.^[^ ^53^, ^54^ ^]^ Given their lack of toxicity even at higher concentrations, shorter, multiple conjugated polyarginines may offer a safer and more versatile approach for protein delivery. We hypothesize that the increased uptake of heterogeneously modified proteins is due to enhanced membrane accumulation by increasing positive charge on the protein surface,^[^ ^16^ ^]^ a concept explored for genetically supercharged proteins.^[^ ^11^, ^55^ ^]^ While protein supercharging requires extensive, meticulous, and laborious genetic manipulations to ensure correct function of the protein, BioRAM offers a straightforward method to transiently supercharge a native protein by simple chemical labeling.

## Conclusion

We report a powerful strategy that enables the serum compatible delivery of native functional proteins into cells. We designed a reducible self‐immolating linker consisting of an activated disulfide to conjugate arginine‐containing cell penetrating peptides (CPPs) to solvent‐accessible primary amines on proteins via an activated carbonate. This bioconjugation, termed BioRAM, modifies native proteins transiently under mild conditions, eliminating the need for genetically engineered proteins. BioRAM was optimized for fast intracellular reduction and complete immolation to recover the native protein upon intracellular delivery, thereby preventing the accumulation of delivered proteins in subcellular compartments. In combination with our previously introduced CPP‐additive technology, the excellent cellular uptake of BioRAM‐protein was demonstrated using the fluorescent protein NLS‐mCherry (26 kDa) as a model cargo. The substantially increased delivery performance over previous methods, in combination with good stability of the bioconjugates, allowed for efficient delivery in the presence of serum. Using an established assay to visualize endosomal escape, our results suggest that protein uptake occurs predominantly through direct translocation with no observable contribution from endosomal escape. Furthermore, we report the delivery of the pharmacologically relevant cytotoxic RNase A (13.7 kDa), for which we obtained low nanomolar effective concentrations, highlighting the efficient combination of BioRAM and the CPP‐additive strategies for cellular delivery of a functional protein.

In light of the highly accessible and applicable nature of our protocol, we envision that BioRAM will be a versatile, useful, and straightforwardly applicable strategy for delivering native proteins into human cells at low concentrations and in the presence of serum, thus paving the way for the delivery of pharmaceutically relevant proteins in more complex systems.

## Conflict of Interests

The authors declare no conflict of interest.

## Supporting information



Supporting Information

## Data Availability

The data that support the findings of this study are available from the corresponding author upon reasonable request.
