# Supplementary material for: Intracellular Delivery of Native Proteins by BioReversible Arginine Modification (BioRAM) on Amino Groups
Source: Angew Chem Int Ed Engl. 2025 Jul 9;64(34):e202506802. doi: 10.1002/anie.202506802 (PMC12363615; doi:10.1002/anie.202506802)

Supporting Information

Intracellular Delivery of Native Proteins by BioReversible Arginine Modification (BioRAM) on Amino Groups

Jonathan Franke^[a][b]^, Jan Vincent V. Arafiles^[a]^, Christian Leis^[a][b]^, Christian P. R. Hackenberger*^[a][b]^

[a] Jonathan Franke, Jan Vincent V. Arafiles, Christian Leis, Christian P. R. Hackenberger
Leibniz-Forschungsinstitut für Molekulare Pharmakologie (FMP)
Robert-Rössle-Strasse 10, 13125 Berlin (Germany)
E-mail: hackenbe@fmp-berlin.de

[b] Jonathan Franke, Christian Leis, Christian P. R. Hackenberger
Department of Chemistry, Humboldt Universität zu Berlin
Brook-Taylor-Straße 2, 12489 Berlin (Germany)

Table of contents

[Supplementary figures 3](#_Toc185340044)

[General Information 18](#_Toc185340045)

[Chemicals and Solvents 18](#_Toc185340046)

[Flash- and thin layer chromatography 18](#_Toc185340047)

[Semi-preparative HPLC 18](#_Toc185340048)

[NMR Spectroscopy 18](#_Toc185340049)

[UPLC-UV/MS UPLC-UV/MS 18](#_Toc185340050)

[HR-MS 18](#_Toc185340051)

[Intact protein-MS 18](#_Toc185340052)

[Native protein-MS 19](#_Toc185340053)

[Protein concentration determination 19](#_Toc185340054)

[Materials 19](#_Toc185340055)

[Experimental procedures 19](#_Toc185340056)

[Protein expression and purification 19](#_Toc185340057)

[Protein-CPP conjugation. 19](#_Toc185340058)

[Maleimide conjugation 19](#_Toc185340059)

[Cysteine disulfide conjugation 19](#_Toc185340060)

[Lysine conjugation 19](#_Toc185340061)

[Cell Culture 20](#_Toc185340062)

[WST-1 assay 20](#_Toc185340063)

[Acute cytotoxicity (mCherry) 20](#_Toc185340064)

[24h Cytotoxicity (RNAse) 20](#_Toc185340065)

[Caspase Assay 20](#_Toc185340066)

[Annexin V binding assay 21](#_Toc185340067)

[Cellular uptake 21](#_Toc185340068)

[Microscopy 21](#_Toc185340069)

[SDS page gel analysis 21](#_Toc185340070)

[Intranuclear fluorescence intensity measurement. 21](#_Toc185340071)

[Software 21](#_Toc185340072)

[Quantification script 22](#_Toc185340073)

[Statistics and Reproducibility 24](#_Toc185340074)

[Peptide Synthesis 25](#_Toc185340075)

[Peptide Synthesis and modifications 25](#_Toc185340076)

[Peptide final deprotection and cleavage, purification, and characterization 25](#_Toc185340077)

[General Synthesis of Peptide-Linker 26](#_Toc185340078)

[Organic Synthesis 32](#_Toc185340079)

[Supplementary References 38](#_Toc185340080)

[NMR Spectra 39](#_Toc185340081)

# Supplementary figures

TNB-

thiol reactive headgroup

R_10_-

decaarginine (CPP)

ILFF-

hydrophobic cell anchor

**Figure S1**. Structure of cell-penetrating-peptide additive consisting of an disulfide-activated cysteine through 5’-thio-2-nitrobenzoic acid, a flexible polyethylenglycol linker, followed by decaarginine and glycin linker, terminated by the hydrophobic anchor I-L-F-F. Please refer to Anselm et al., *Nature Chemistry* 2021^[1]^ for the discovery of the CPP-additive strategy and Arafiles et al., *JACS* 2023 ^[2]^ for further structural improvements resulting in the potent CPP-additive used for this study.

**Figure S2**. Synthesis of β-mercaptoethanol derivatives 1a-b followed by disulfide exchange yielding SI 3a-c and carbonate formation to give compounds 2a-c.

|  | **pH 4.35, 20 °C** | | **pH 7.4, 20 °C** | |
| --- | --- | --- | --- | --- |
| **cysteine (equiv.)** | **1** | **10** | **1** | **140** |
| **2a** | conversion | - | - | - |
| **2b** | conversion* | - | conversion+  degradation | - |
| **2c** | stable | stable | stable | 10% degradation |

**Figure S3**. Reactivity of compounds 2a-c towards cysteine under different conditions over 3 hours (* = 24 hours) in pH-buffered solution containing 50% acetonitrile.


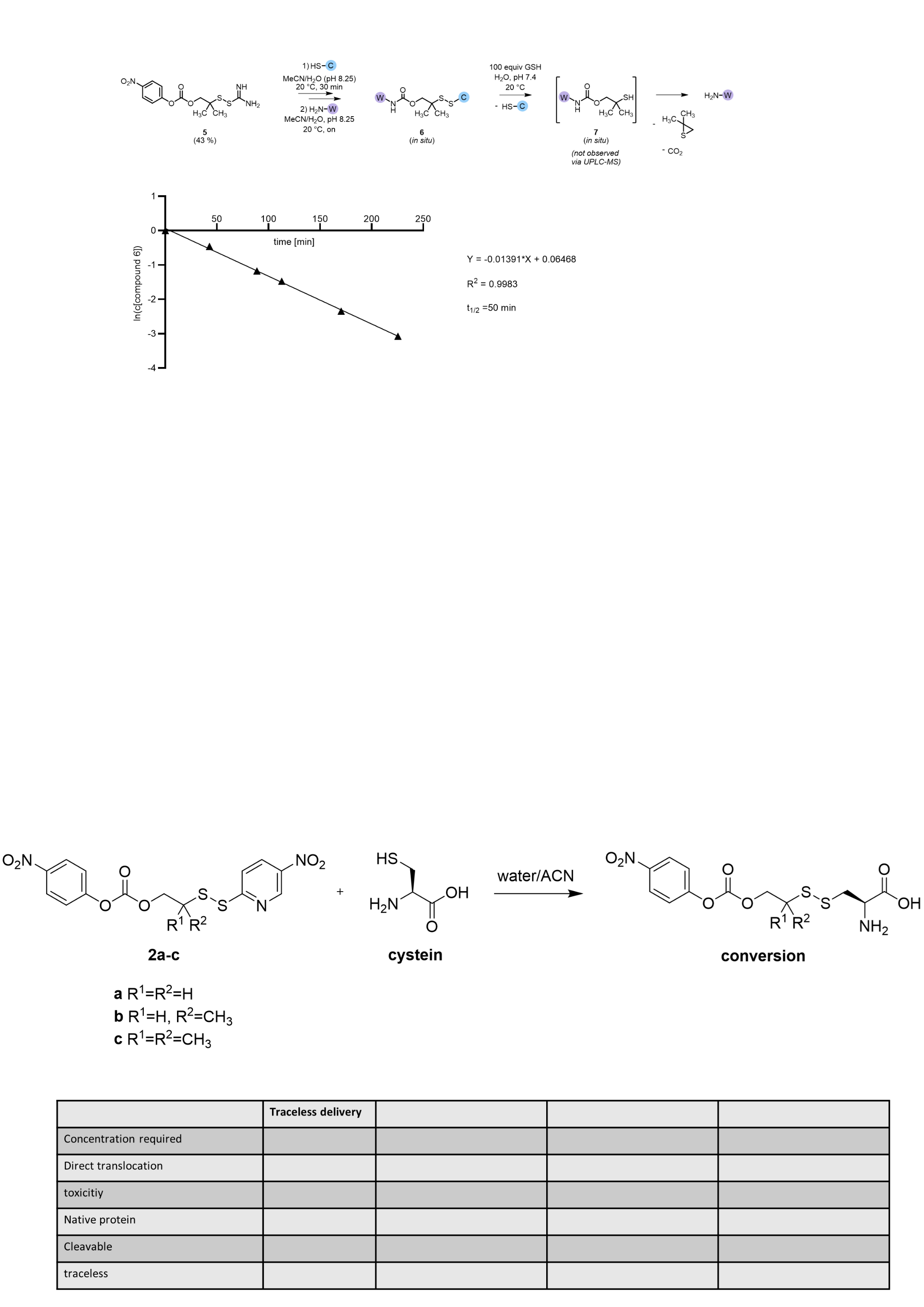


**Figure S4.** Stability of dimethylated disulfide 6 under reducing conditions. The reaction product resulting from disulfide exchange using fmoc-cysteine was purified and reacted with tryptophan at pH 8.2 overnight. The resulting reaction mixture was diluted into 400 µL PBS yielding a 0.1 mM solution of 6 (assuming full conversion in the overnight reaction) and spiked with Ph_3_O (0.4 mM) as an internal standard as well as reduced glutathione (GSH) (10 mM, 100 equiv). The reaction was monitored by UPLC-MS at room temperature. The consumption of starting material was monitored and plotted assuming a first-order reaction kinetic.


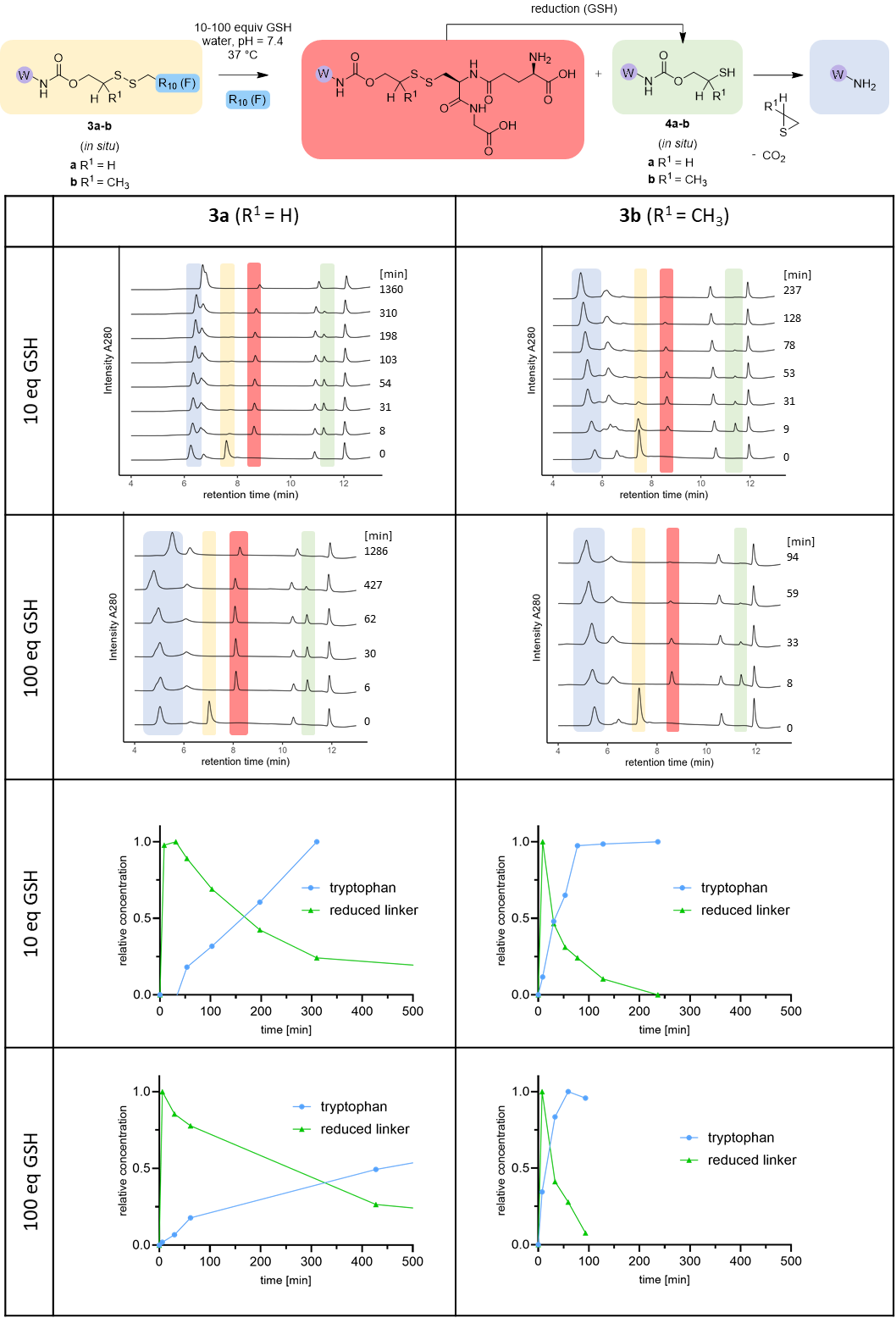


**Figure S5.** *In situ* reduction of formed compounds 3a-b (0.1 mmol) upon incubation with 10 or 100 equiv. of reduced GSH (1 and 10 mM, respectively). The reduction and immolation rates of the reduced linker were calculated using analytical HPLC. While the fluorescently labeled peptide was initially intended for detection via fluorescence HPLC, monitoring the different species at 280 nm (tryptophan absorption) proved to be more conclusive.

**Figure S6**. As proposed in literature, the increased steric hinderance decreases the probability of nucleophilic attack by a thiol and succeeding reduction of the disulfide bond.^[3]^ In case of a proximal thiol, the nucleophilic attack towards the sulfur with unsubstituted alpha carbon is favored, releasing the free thiol, in place for the intramolecular reaction. The cyclization has been reported to proceed primarily via the thiirane instead of the five membered heterocycles.^[4]^ The speed of the reaction is significantly accelerated by α-substitution on the carbon C1 due to the Thorpe-Ingold effect.^[5]^


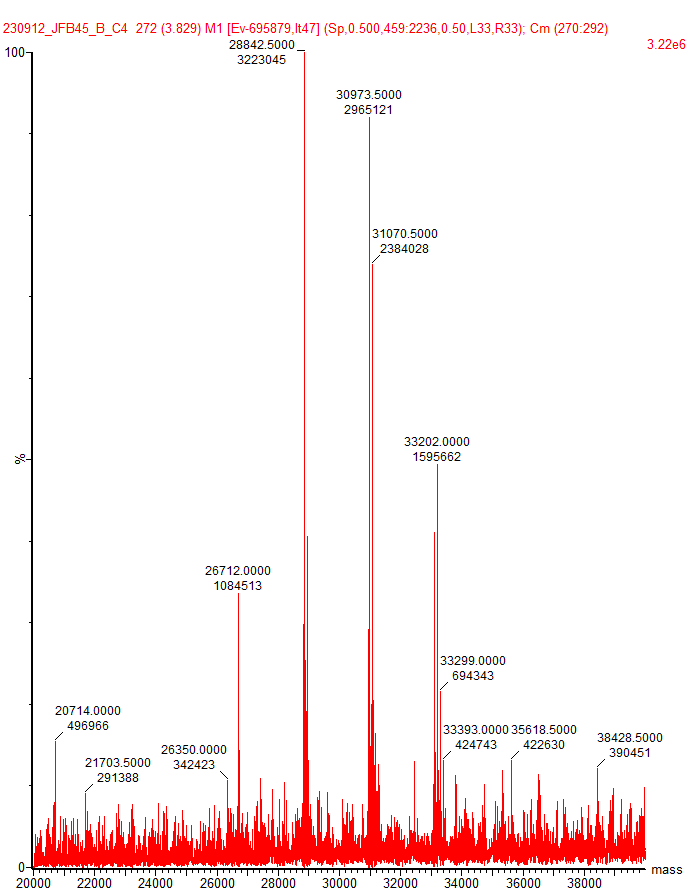


| **modification** | **0** | **1** | **2** | **3** | **4** | **5** |
| --- | --- | --- | --- | --- | --- | --- |
| Mass of modification (**8b**) |  | 2130 | 4260 | 6390 | 8520 | 10650 |
| resulting protein masses | 26712 | 28842 | 30972 | 33102 | 35232 | 37362 |

**Figure S7**. Deconvoluted protein spectra of sfGFP modified with compound 8b, analyzed using different methods. Protein-CPP conjugates were initially analyzed using standard protein analysis by QTof HR-MS that involves separation by a C4 column at 80 °C (left); however, this resulted in partial fragmentation of the protein conjugate. Switching to native mass spectrometry by direct injection of the protein into the ESI-MS showed considerably less fragmentation and improved the resulting mass spectrum (right).^[6]^. The number of modifications is summarized in the accompanying table.


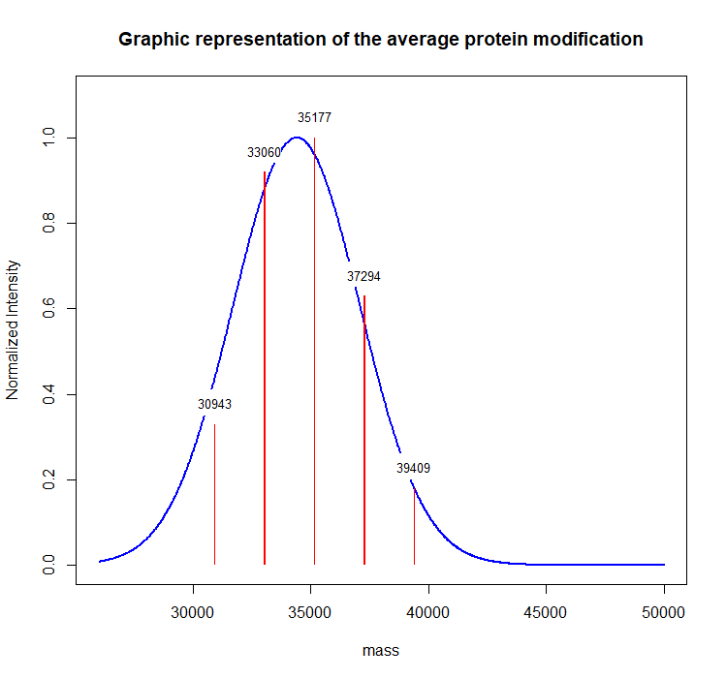

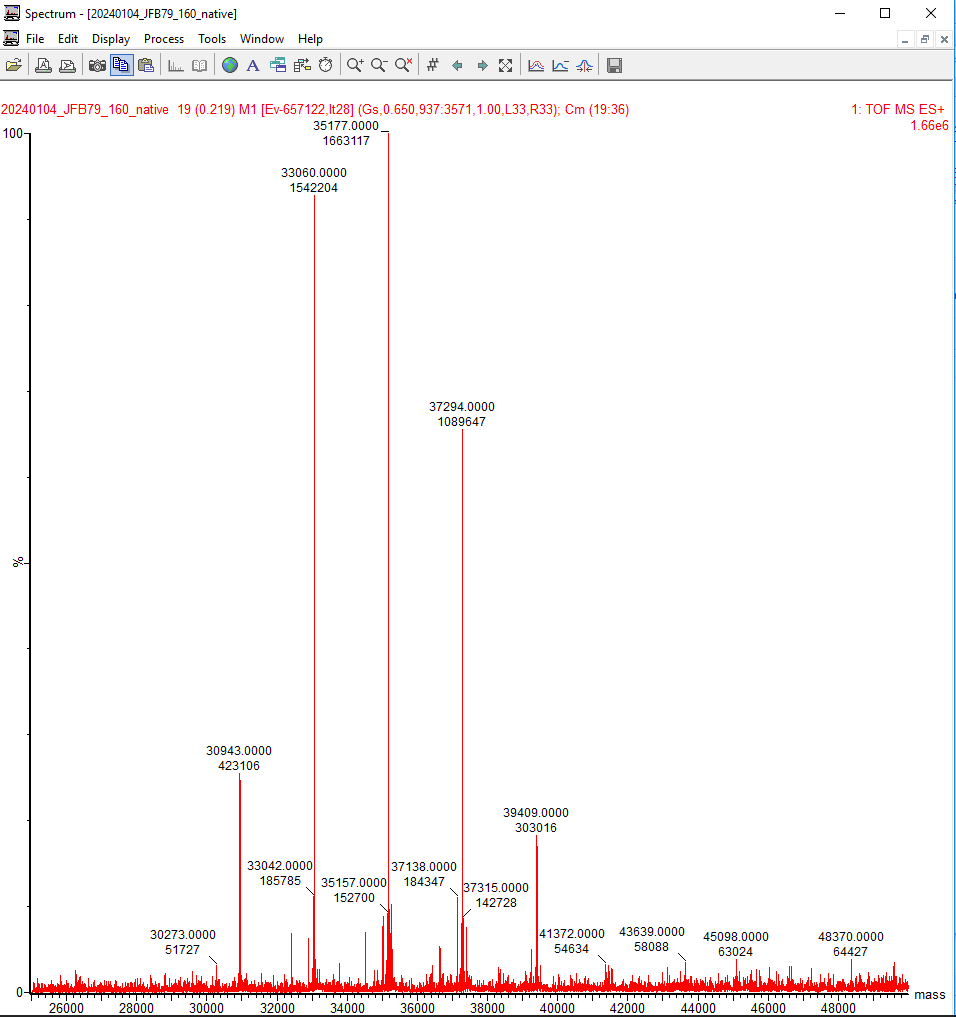

$$weighted average= \frac{\sum_{i=1}^{n} \left( x_{i}*w_{i} \right)}{\sum_{i=1}^{n} x_{i}}$$

$$w_{i}=number of modifications corresponding to this protein peak$$

$$x_{i}=relative intensity$$

**Added arginines per protein:**

$$charge= \frac{\sum_{i=1}^{n} \left( x_{i}*w_{i} \right)}{\sum_{i=1}^{n} x_{i}}*n_{R}$$

$$n_{R}=number of arginines on peptide-linker used [n_{R}=10/8/6/4]$$

**Added charge per protein (considering, that one lysine is occupied for conjugation):**

$$charge= \frac{\sum_{i=1}^{n} \left( x_{i}*w_{i} \right)}{\sum_{i=1}^{n} x_{i}}*(n_{R}-1)$$

$$n_{R}=number of arginines on peptide-linker used [n_{R}=10/8/6/4]$$

**Figure S8.** Calculation of the average protein modification (apm) based on the deconvoluted native protein spectrum assuming gaussian distribution of the modified species. Formula for the calculation of added arginine and charge per protein using BioRAM.

A

C

E

B

D

F

2

3

4

5

6

7

9

8

10

11

12

2

3

4

5

1

0

**Figure S9**. QTof HR-MS spectra from immolation of BioRAM GFP-**D** (A-11 min, B-29 min, C-48 min, D-83 min, E-128 min, F-250 min, mass list [Da] (number of modifications): 26714 (0), 26817 (1), 26921 (2), 27024 (3), 27127 (4), 27230 (5), 27332 (6), 27435 (7), 27537 (8), 27639 (9), 27740 (10), 27844 (11), 27947 (12) and BioRAM GFP-**E** (G-10 min, H-30 min, I-48 min, J-70min, mass list [Da]: 26714 (0), 26831 (1), 26949 (2), 27067 (3), 27254 (4), 27372 (5), 27490(6)).

G

I

H

J

1

2

3

4

5

6

7

0

1

2

**
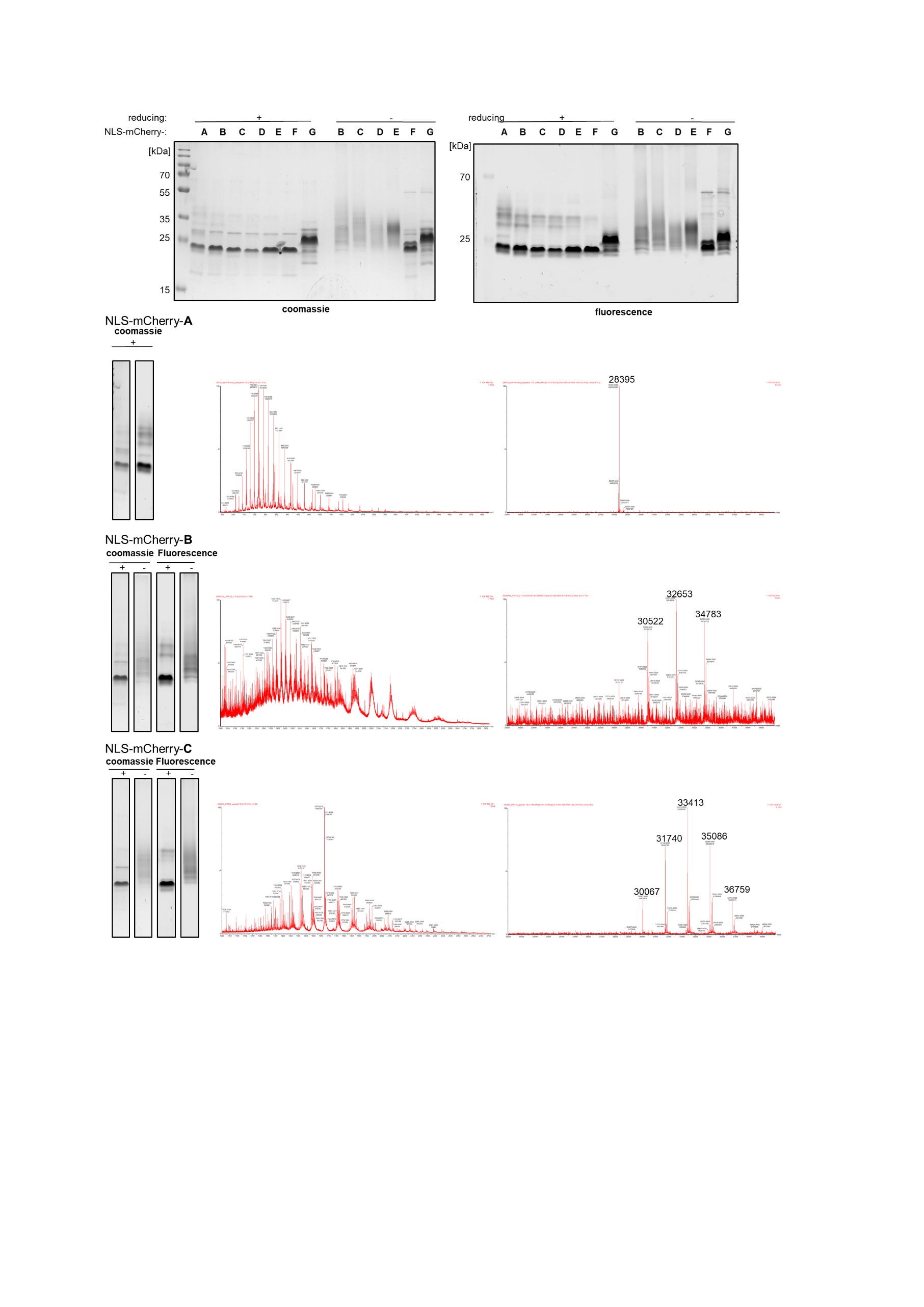
**

**
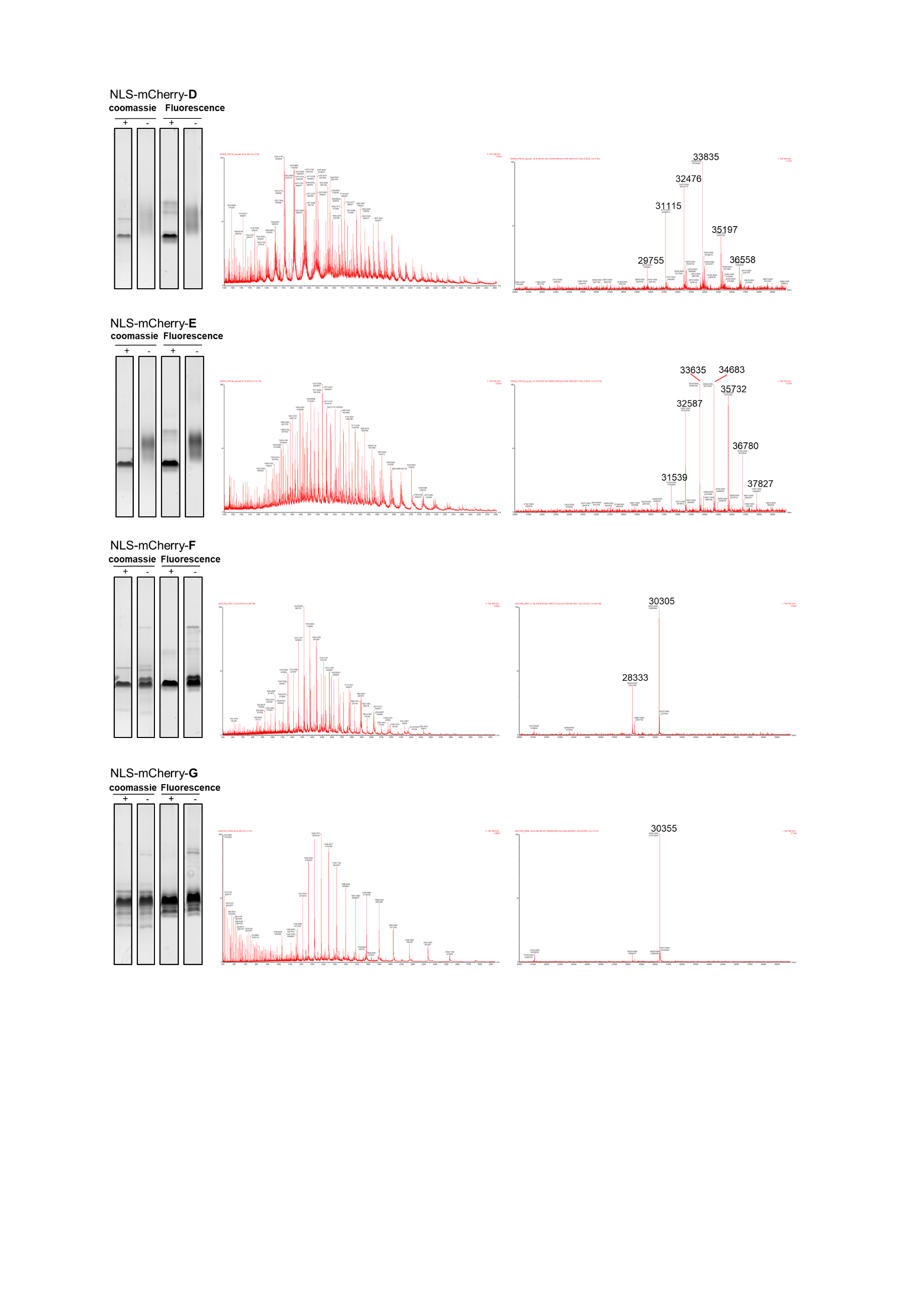
Figure S10**. Characterization of NLS-mCherry-BioRAM bioconjugates using (fluorescent) SDS-PAGE gel analysis and native QToF HR-MS analysis. Fluorescence was recorded using the Alexa 547 channel. Deconvoluted spectra (right), mass in Dalton: (NLS-mCh-A) calcd.: 28394; found: 28395; (NLS-mCh-B) calcd.: 30525, 32655, 34786; found: 30522, 32653, 34783; (NLS-mCh-C) calcd.: 30067, 31740, 33413, 34086, 36759; found: 30067, 31740, 33413, 34086, 36759; (NLS-mCh-D) calcd.: 29756, 31116, 32477, 33838, 35198, 36559; found: 29755, 31115, 32476, 33835, 35197, 36558; (NLS-mCh-E) calcd.: 31539, 32587, 33635, 34684, 35732, 36780, 37828; found: 31539, 32587, 33635, 34683, 35732, 36780, 37827.


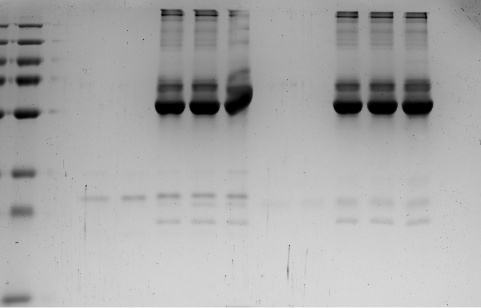


35

55

[kDa]

NLS-mCh-**G**

25

-

+

reducing

-

-

-

NLS-mCh-**A**

NLS-mCh-**G**

NLS-mCh-**A**

1h

4h

24h

1h

4h

24h

1h

4h

24h

1h

4h

24h

-

+

-

-

-

-

+

-

-

-

-

+

-

-

-


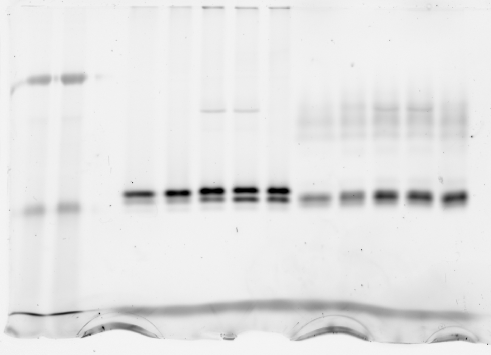

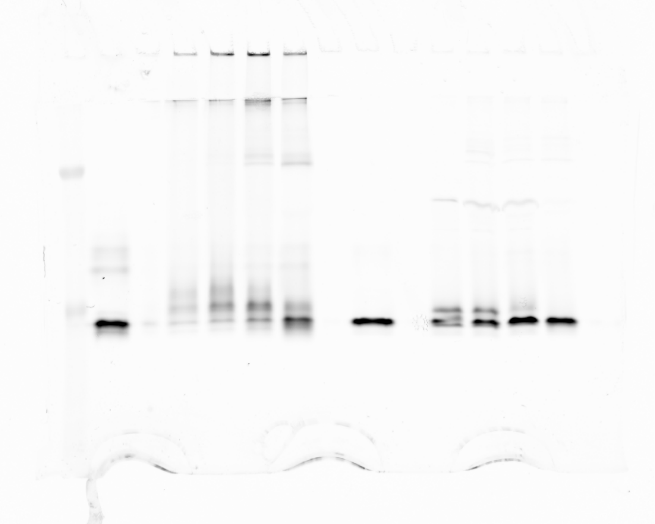

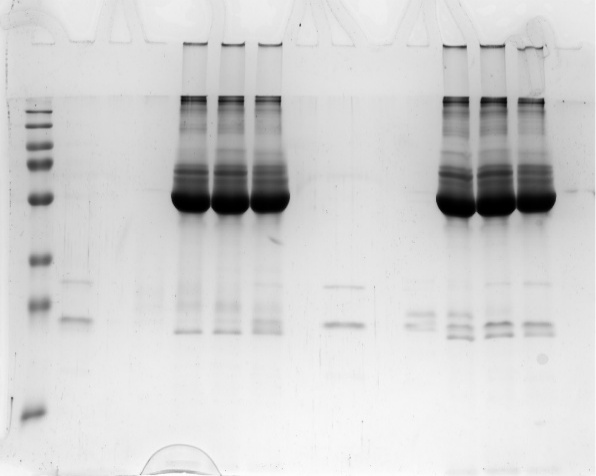


70

55

[kDa]

25

35

reducing

NLS-mCh-**B**

NLS-mCh-**F**

1h

4h

24h

-

+

-

-

-

70

25

1h

4h

24h

-

+

-

-

-

1h

4h

24h

-

+

-

-

-

1h

4h

24h

-

+

-

-

-

NLS-mCh-**B**

NLS-mCh-**F**

**coomassie**

**Alexa546**

**Alexa546**

**coomassie**

70

25

[kDa]

70

[kDa]

25


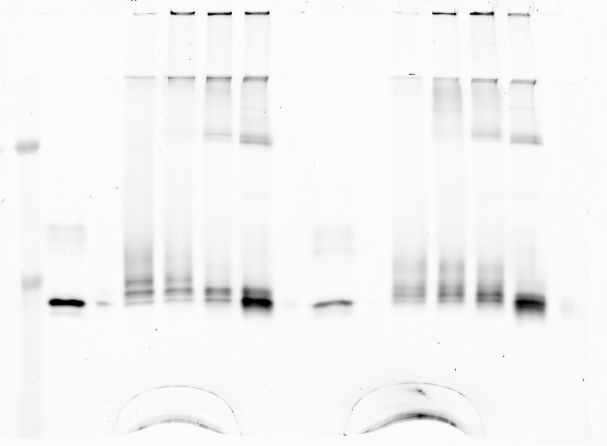


NLS-mCh-**C**

NLS-mCh-**F**

1h

4h

24h

-

+

-

-

-

1h

4h

24h

-

+

-

-

-


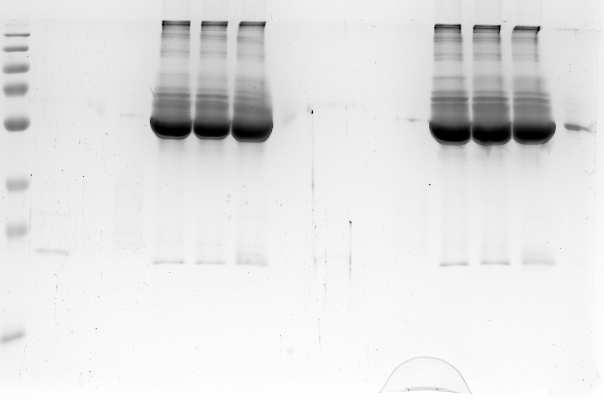


[kDa]

NLS-mCh-**C**

NLS-mCh-**F**

1h

4h

24h

-

+

-

-

-

1h

4h

24h

-

+

-

-

-

70

55

25

35

**Alexa546**

**coomassie**

reducing


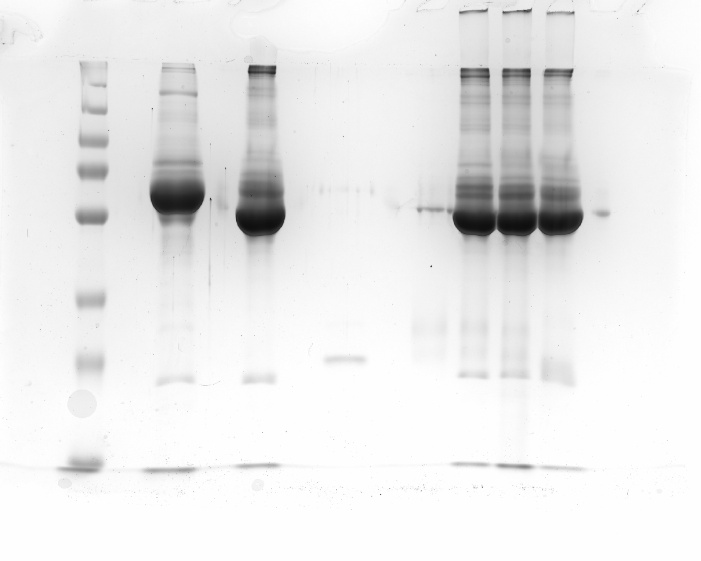

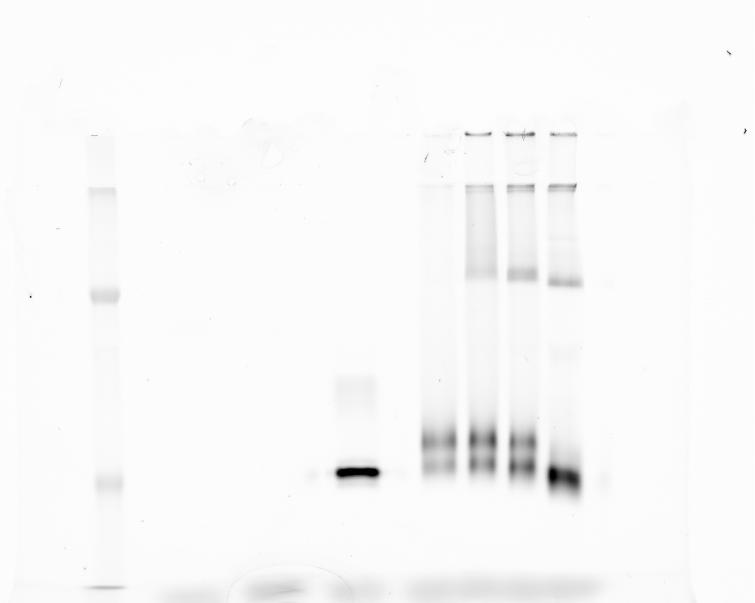


NLS-mCh-**E**

1h

4h

24h

-

-

-

-

-

+

Medium, 10 % FCS

**Alexa546**

**coomassie**

NLS-mCh-**E**

1h

4h

24h

-

-

-

-

-

+

Medium, 10 % FCS

[kDa]

70

55

25

35

reducing

+

**Figure S11.** SDS-PAGE gel analysis of NLS-mCherry-BioRAM bioconjugates stability in cell culture medium (DMEM-HG, 10 % FCS) over 1, 4 and 24 h. Samples were not heated during the sample preparation to maintain fluorescent properties of mCherry and only reduced if indicated (+).

**Figure S12**. A) Representative confocal microscopy images from the automated acquisition during quantification measurements in 96 well plate (Nikon 40x air) after treatment with fluorescent protein. Incubation of HeLa cells using 2.5 µM protein in the presence of 5 µM additive in growth medium (DMEM-HG) containing 10 % FCS for 1 hour, followed by two washing steps with heparin in PBS and addition of imaging medium (Fluorobrite DMEM) supplemented with 10 % FCS. Contrast and microscopy settings are equal for all images. Scale bar = 20 µm. B) Schematic representation of quantification workflow performed post acquisition. Representative confocal microscopy images from incubation of cells with 2.5 µM NLS-mCh-E in the presence of 5 µM additive (Nikon 60x oil).

Merge

(mCherry+hoechst)

mCherry

mCherry (mask)

**2.5 uM protein, 5 uM additive, 10 % FCS, 1 hour**

NLS-mCh-**B**

(3b-R_10_)

NLS-mCh-**C**

(3b-R_8_)

NLS-mCh-**D**

(3b-R_6_)

NLS-mCh-**E**

(3b-R_4_)

NLS-mCh-**G**

(maleimid-R_10_)

NLS-mCh**-A**

(mCh)


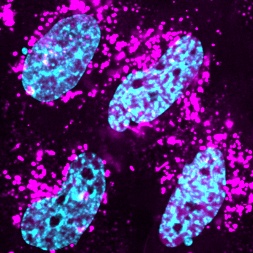

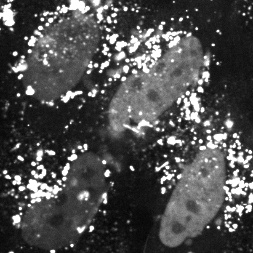

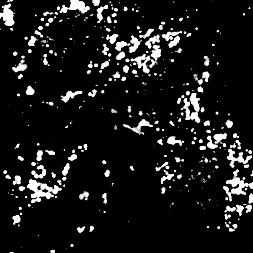

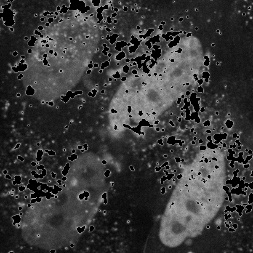

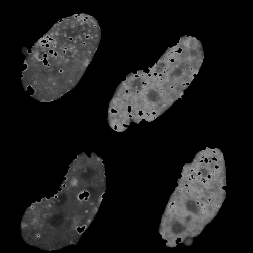

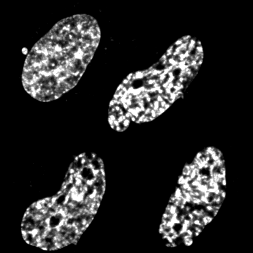


**=**

**+**

Endosomes and aggregates

Diffused signal

Diffused nuclear signal

Nucleus

(hoechst)

mCherry

Composite (mCherry+hoechst)

Quantification

using median fluorescence per area

**B**

**A**


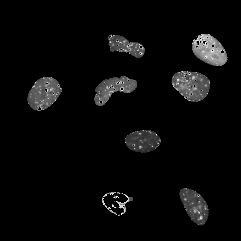

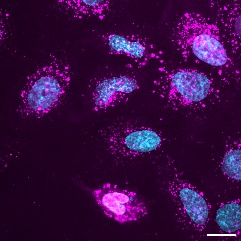

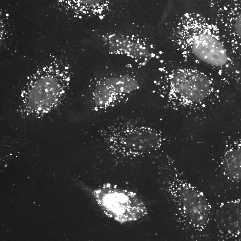

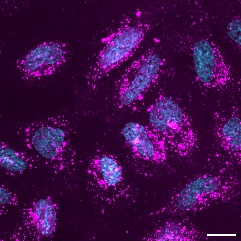

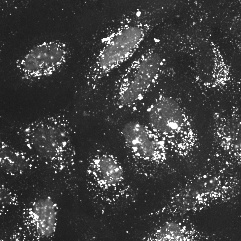

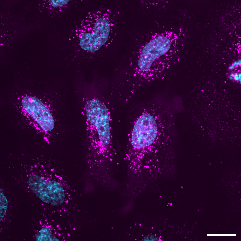

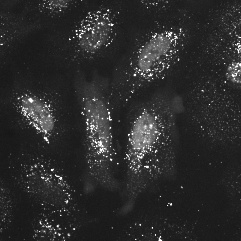

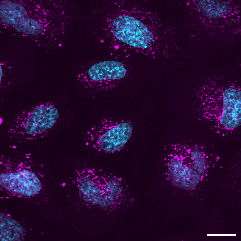

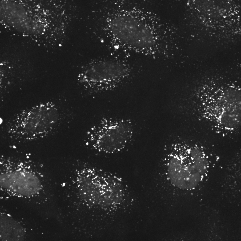

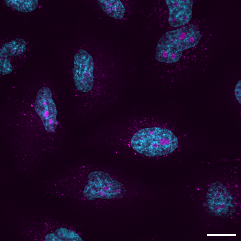

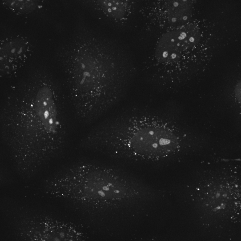

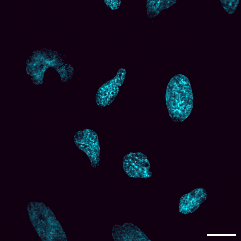

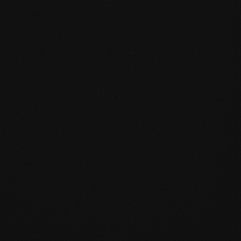

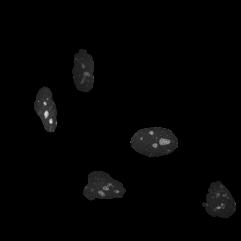

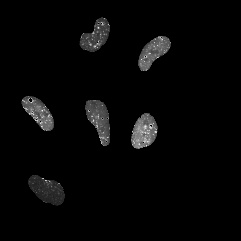

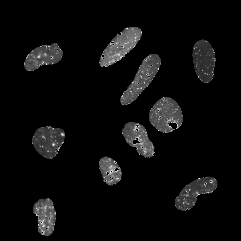

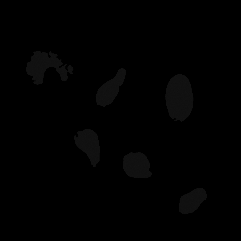

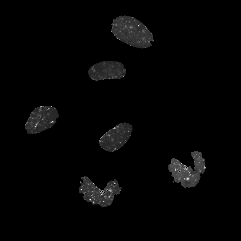


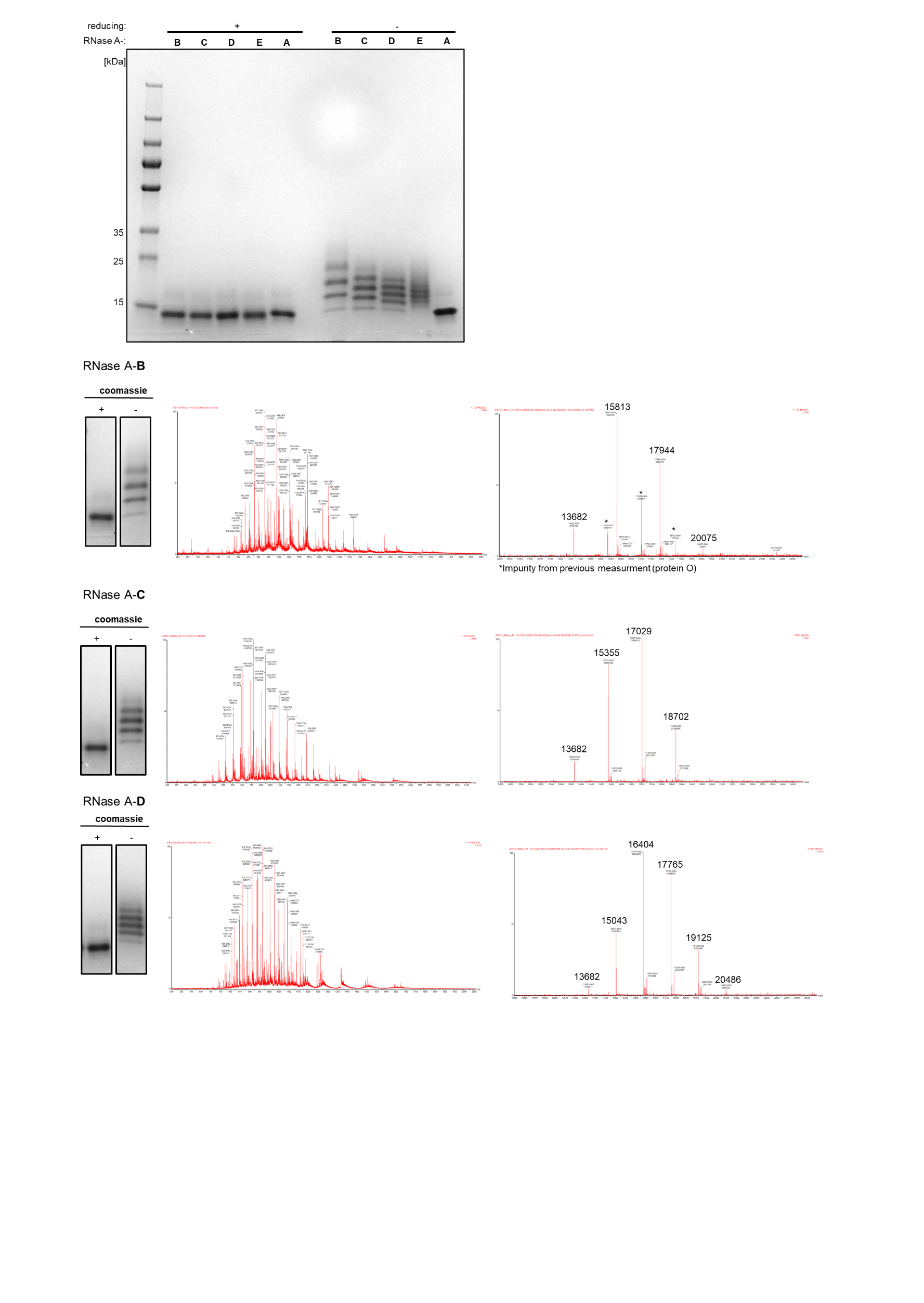


**
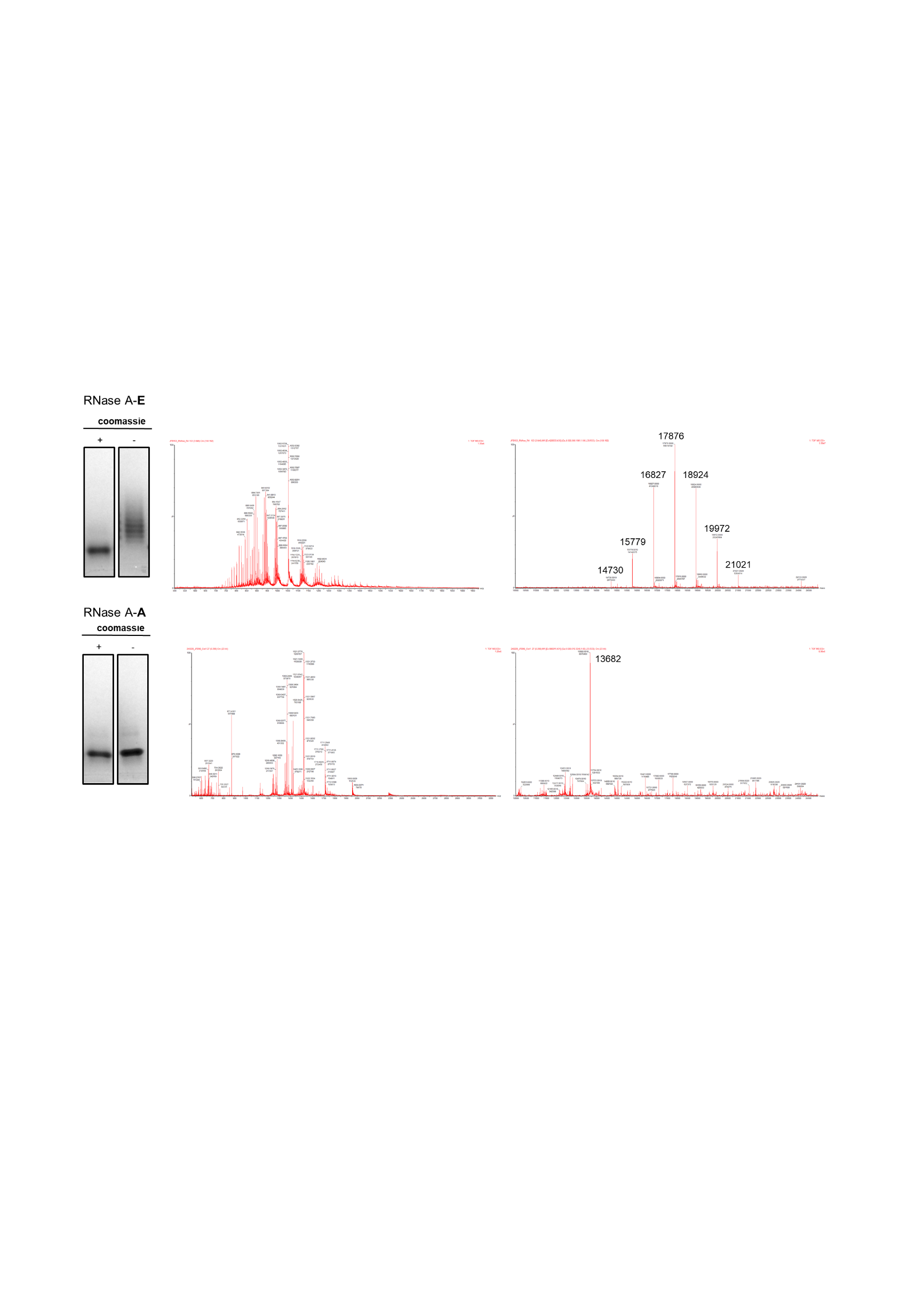
Figure S13**. Characterization of RNase A bioconjugates using SDS-PAGE gel analysis and QToF HR-MS analysis. Deconvoluted spectra (right), mass in Dalton: (RNase A-B) calcd.: 15813, 17943, 20074; found: 15813, 17944, 20075; (RNase A-C) calcd.: 15355, 17028, 18701; found: 15355, 17028, 18701; (RNase A-D) calcd.: 15043, 16403, 17764, 19125, 20485; found: 15043, 16404, 17765, 19125, 20486 ; found: 28395; (RNase A-E) calcd.: 14730, 15779, 16827, 17875, 18923, 19972, 21021; found: 14730, 15779, 16827, 17876, 18924, 19972, 21021; (RNase A-A) found: 13682.

**A**

**B**

**C**


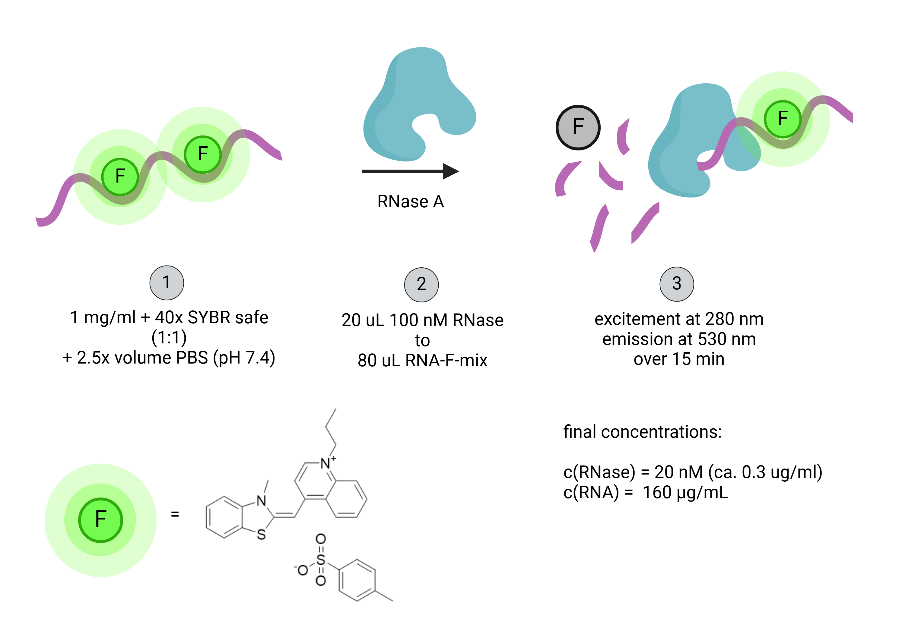

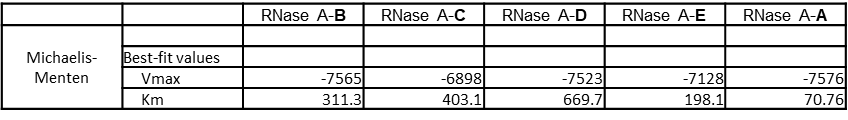


**Figure S14**. A) Schematic representation of RNase A activity assay using SYBR-safe fluorophore. B) Enzymatic RNase A activity measured by decrease in fluorescence intensity related to RNA degradation C) Michealis Menten parameters calculated using Graphpad PRISM.


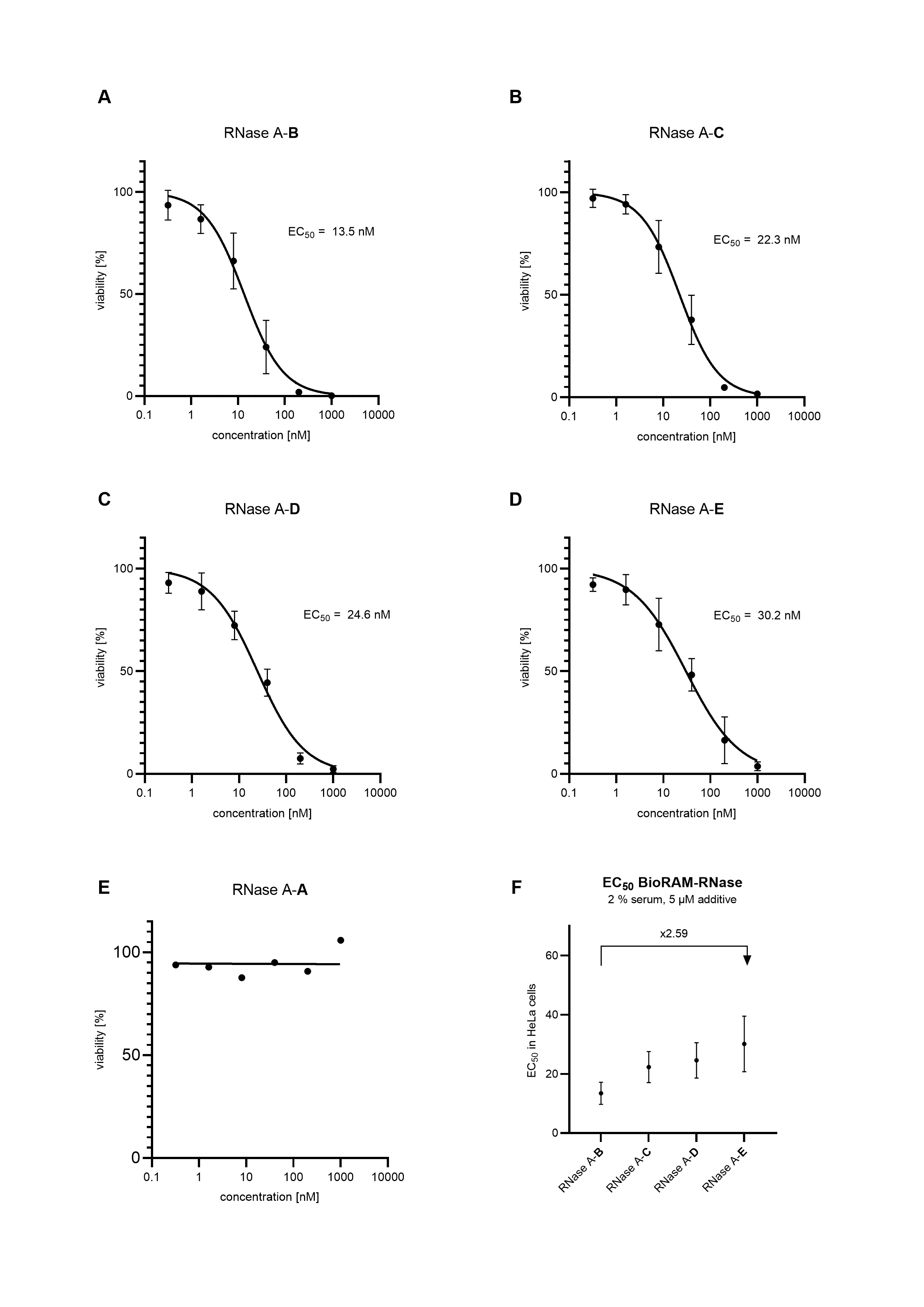
**Figure S15**. A-E) Cell viability of HeLa cells after 24 h of treatment with RNase A-BioRAM conjugates RNase A-B (A), RNase A-C (B), RNase A-D (C), RNase A-E (D), RNase A-A (E) (Data presented as mean ± standard deviation (SD) of three biological replicates (N=3), N=1 for RNase A-A). F) SD of EC_50_ was calculated from 95% confidence intervals and used for One-way Analysis of Variance (ANOVA). Fold-change of EC_50_ (RNase A-B) and EC_50_ (RNase A-E) is indicated.

**Figure S16**. Cell viability of HeLa cells after 24 h of treatment with RNase A-BioRAM conjugates without TNB-R_10_-ILFF additives (Data presented as mean ± SD of three technical replicates (N=3)).


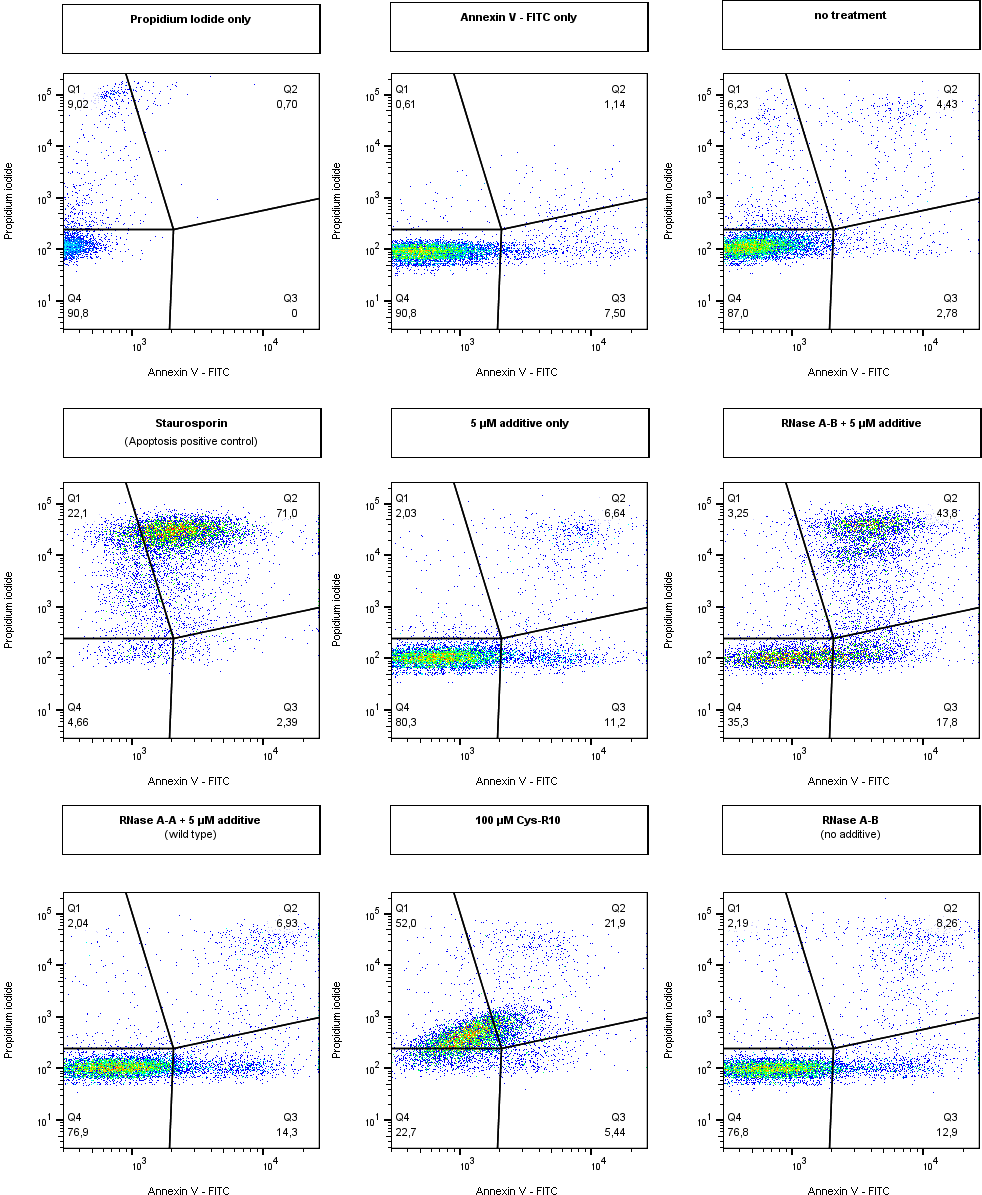


**Figure S17**. Populations of cells detected by flow cytometry analysis to differentiate into Q1 – necrotic, Q2 – late apoptotic, Q3 – early apoptotic, Q4 -viable cells. One of three replicates displayed. Gates were chosen based on staurosporin control and non-treated cells.

# General Information

## Chemicals and Solvents

Chemicals and solvents were purchased from Sigma-Aldrich (Merck Group, Germany), TCI (Tokyo chemical industry CO., LTD., Japan) and Acros Organics (Thermo Fisher scientific, USA), BLD Pharm (BLD Pharmatech, China) and used without further purification. Dry solvents were purchased from Acros Organics (Thermo Fisher scientific, USA). Aminoacids, coupling reagents and resins for SPPS were purchased from Novabiochem (Merck, USA) or Iris Biotech GmbH (Germany).

## Flash- and thin layer chromatography

Flash column chromatography was performed, using NORMASIL 60® silica gel 40-63 μm (VWR international, USA). Analytical thin layer chromatography (TLC) was performed on aluminum foil pre-coated with SiO_2_-60 F254 (Macherey-Nagel, DE). Spots were visualized by fluorescence depletion with a 254nm lamp or manganese staining (10 g K_2_CO_3_, 1.5 g KMnO_4_, 0.1 g NaOH in 100 mL H_2_O), followed by heating.

## Semi-preparative HPLC

Semi-preparative HPLC was performed on a Shimadzu prominence HPLC system (Shimadzu Corp., Japan) with a CBM20A communication bus module, a FRC-10A fraction collector, 2 pumps LC-20AP, and a SPD-20A UV/VIS detector, using a VP250/21 Macherey-Nagel Nucleodur C18 HTec Spum column (Macherey-Nagel GmbH & Co. Kg, Germany).

## NMR Spectroscopy

NMR spectra were recorded with a Bruker Avance III 600 MHz spectrometer (Bruker Corp., USA) at ambient temperature. Chemical shifts δ are reported in ppm relative to residual solvent peak (CDCl_3_: 7.26 [ppm]; DMSO-d_6_: 2.50 [ppm]; 4.79 D_2_O [ppm] for ^1^H-spectra and CDCl_3_: 77.16 [ppm]; DMSO-d_6_: 39.52 [ppm]; for ^13^C-spectra. Coupling constants *J* are stated in Hz. Signal multiplicities are abbreviated as follows: s: singlet; d: doublet; t: triplet; q: quartet; m: multiplet.

## UPLC-UV/MS UPLC-UV/MS

Samples were recorded on a Waters H-class instrument equipped with a quaternary solvent manager, a Waters autosampler, a Waters TUV detector and a Waters Acquity QDa detector with an Acquity UPLC BEH C18 1.7 μm, 2.1 x 50 mm RP column with a flow rate of 0.6 mL/min (Waters Corp., USA). The following gradient was used: 0.1 % TFA in H_2_O; B: 0.1% TFA in MeCN. 5 % B 0 - 0.5 min, 5-95 % B 0.5-3 min, 95 % B 3-3.9 min, 5 % B 3.9-5 min.

## HR-MS

High resolution ESI-MS spectra were recorded on a Waters H-class instrument equipped with a quaternary solvent manager, a Waters sample manager-FTN, a Waters PDA detector and a Waters column manager with an Acquity UPLC protein BEH C18 column (1.7 μm, 2.1 mm x 50 mm). Samples were eluted with a flow rate of 0.3 mL/min. The following gradient was used: “QTof”: 0.01% FA in H_2_O; B: 0.01% FA in MeCN. 5 % B: 0-1 min; 5 to 95 % B: 1-7min; 95 % B: 7 to 8.5 min. Mass analysis was conducted with a Waters XEVO G2-XS QTof analyzer. (A significant portion of small molecules could not be detected by QTof HR-MS due to their inherent instability upon ionization.)

## Intact protein-MS

Intact proteins were analyzed using a Waters H-class instrument equipped with a quaternary solvent manager, a Waters sample manager-FTN, a Waters PDA detector and a Waters column manager with an Acquity UPLC protein BEH C4 column (300 Å, 1.7 μm, 2.1 mm x 50 mm). Proteins were eluted with a flow rate of 0.3 mL/min with 80 °C column temperature. The following gradient was used: A: 0.01 % FA in H_2_O; B: 0.01 % FA in MeCN. 5-95 % B 0-6 min. Mass analysis was conducted with a Waters XEVO G2-XS QTof analyzer. Raw data was deconvoluted with MaxEnt 1.

Protein-CPP conjugates were initially analyzed using standard protein analysis by QTof HR-MS that involves separation by a C4 column at 80 °C; however, this resulted in partial fragmentation of the protein conjugate. Switching to native mass spectrometry by direct injection of the protein into the ESI-MS showed considerably less fragmentation and improved the resulting mass spectrum (Figure S6).^[6]^

## Native protein-MS

Native protein MS was conducted with proteins in volatile buffer (ammonium bicarbonate or ammonium acetate at 20 mM). 10 μL of adequately diluted protein were directly injected into a Waters H-class instrument (see intact protein-MS). Mass analysis was conducted with a Waters XEVO G2-XS QTof analyzer. Raw data was deconvoluted with MaxEnt 1.

## Protein concentration determination

Protein concentrations were determined by absorption spectroscopy measurements at 280 nm using the extinction coefficient and molecular weight of the protein on a NanoDrop one (Thermo Scientific, USA).

## Materials

Amino acids, rink amide resin and coupling reagents were purchased from Iris Biotech (Germany). HATU was purchased from Bachem (Switzerland). DIEA, TFA, Salts, LB medium, antibiotics and other buffer components were purchased from Carl Roth (Germany). Mammalian cell culture media and fetal bovine serum were purchased from BioWest and Gibco, respectively. Zeba™ Spin Desalting columns were purchased from Thermo Scientific (USA).

# Experimental procedures

## Protein expression and purification

### Superfolded GFP (sf-GFP)

Protein sequence (sequence after *Tobacco Etch Virus nuclear-inclusion-a endopeptidase* (TEV) cleavage side is underlined):

HHHHHHSSGLVPRGSHMASENLYFQGRKGEELFTGVVPILVELDGDVNGHKFSVRGEGEGDATNGKLTLKFICTTGKLPVPWPTLVTTLTYGVQCFARYPDHMKQHDFFKSAMPEGYVQERTISFKDDGTYKTRAEVKFEGDTLVNRIELKGIDFKEDGNILGHKLEYNFNSHNVYITADKQKNGIKANFKIRHNVEDGSVQLADHYQQNTPIGDGPVLLPDNHYLSTQSVLSKDPNEKRDHMVLLEFVTAAGITHGMDELYK

For the expression, BL21 DE3 cells were transformed with the pET28a plasmid. A single colony from an agar plate was picked and grown for 24 hours at 37 °C in 5 mL of LB medium with 40 µg/mL Kanamycin. The starting culture was then expanded to 1.2 L at 37 °C until OD_600_ reached 0.8, followed by induction using 0.5 mM IPTG and incubation at 18 °C over night under agitation.

Cells were collected by centrifugation at 4000× *g* for 15 min. The cells were washed once in PBS, then resuspended in PBS with 20 mM imidazole and 2 mM dithiothreitol (DTT) and lysed using sonication (3×, 2 min, 25 % Amplitude), followed by centrifugation at 25000× *g* for 30 min. For the purification, the clear lysate was loaded on PureCube 100 Ni-INDIGO agarose. The beads were washed with 20 column volumes (CV) of 10 mM Dulbecco’s phosphate buffered saline (DPBS) with 20 mM imidazole. The protein was then eluted using 10 mM DPBS containing 500 mM imidazole. The purification tag was removed by the addition of TEV (1:10 (*w*/*w*)) overnight at room temperature in dialysis against 10 mM PBS followed by reverse nickel purification to remove the protease TEV. The sfGFP containing follow-through was shock-frozen as aliquots and stored at −80 °C.

### NLS-mCherry-Cysteine

The protein was expressed as published previously.^[1, 7]^ Protein sequence (sequence after thrombin cleavage underlined, engineered cysteine in blue):

MGSSHHHHHHSSGLVPRGSHMPAAKRVKLDMVSKGEEDNMAIIKEFMRFKVHMEGSVNGHEFEIEG EGEGRPYEGTQTAKLKVTKGGPLPFAWDILSPQFMYGSKAYVKHPADIPDYLKLSFPEGFKWERVMN FEDGGVVTVTQDSSLQDGEFIYKVKLRGTNFPSDGPVMQKKTMGWEASSERMYPEDGALKGEIKQRL KLKDGGHYDAEVKTTYKAKKPVQLPGAYNVNIKLDITSHNEDYTIVEQYERAEGRHSTGGMDELYKACA*

## Protein-CPP conjugation.

### Maleimide conjugation

To conjugate the maleimide-functionalized R_10_ to NLS-mCherry, the protein (200 µmol) was diluted to 100 μM concentration in PBS and dithiothreitol (DTT) was added to a final concentration of 1 mM (final volume of 500 µL). The protein was desalted by gel filtration using ZebaSpin 7kDa MWCO spin column (Thermo Scientific, USA). 5 equiv. of maleimide-R_10_ were added, and the solution was incubated overnight at 4 °C. Excess peptides were removed by Zeba™ Spin filtration (7k MWCO).

### Cysteine disulfide conjugation

For disulfide conjugation (NLS-mCherry-SS-R_10_) 5 equiv. of NPYS-R_10_ was added to purified 100 µM NLS-mCherry and was allowed to react for 2 h. Excess peptide and leaving group (NPYS) was desalted by Zeba™ Spin filtration (7k MWCO).

### BioRAM conjugation to aliphatic amines

50 µM of NLS-mCherry (100 µM of RNAse) was incubated with respective peptide (3b-R_4_ to 3b-R_10_, Note 1) at 100-1000 µM (2-20 equiv., Note 2) in HEPES buffer (200 mM HEPES at pH = 8.25, 140 mM NaCl). Test reactions were performed in 20 µL, upscale reactions were done using a total volume of 500 µL for NLS-mCherry (120 µL for RNAse). The reaction mixture was incubated at 20 °C for 3 hours at 500 rpm shaking. The reaction mixture develops a yellow color due to the formation of nitrophenol, a byproduct generated during successful protein conjugation as well as from gradual hydrolysis over time. The resulting modified protein was separated from excess peptide by using an Amicon® spin filtration (Amicon® Ultra-0.5, 3-10k MWCO) until a 1:100 dilution, followed by desalting by Zeba™ Spin purification to remove remaining small molecules and potential aggregates. Proteins were rebuffered within the Zeba™ Spin purification step in storage buffer (5 mM HEPES at pH = 7.5, 140 mM NaCl, 2.5 mM KCl, 5 mM Glycine). Protein concentration was determined using a Nanodrop (Nanodrop One/OneC, Thermo Scientific, USA). The samples were frozen in liquid nitrogen and stored at -80 °C.

**Note 1:**

The peptide was used from a stock in water, stored at -20°C. No degradation was observed within a maximum of 5 freeze-thaw cycles.

**Note 2:**

Higher concentrated reactions usually yield higher conversions. More equivalents of peptide might have to be added when performing the bioconjugation at lower protein concentrations

**Note concerning NLS-mCherry protein (free cysteines):**

The engineered cysteine in the NLS-mCherry construct is not required for the conjugation of CPPs using the herein described linker. We reasoned that a free cysteine could form labile thioesters with the carbonates, potentially interact with disulfides of the linker by thiol exchange or interfere with the additive incubation protocol. Therefore, the thiol was alkylated prior to CPP to lysine conjugations using 10 equiv. Iodoacetamide for 1 hour. The protein was desalted using Zeba™ Spin filtration (7k MWCO).

## Immolation on protein

sfGFP-BioRAM conjugates were prepared and purified using the general protocol. Proteins were diluted to 1 µM using PBS (pH = 7.4) and incubated at 37 °C. Reduction and subsequent immolation was initiated by the addition of GSH to a final concentration of 1 mM. The reaction was monitored using intact protein QTof HR-MS (Figure S8).

## Cell Culture

All cell incubation and treatments were done in a humidified incubator at 37 °C with 5 % CO_2_. HeLa were cultivated in Dulbecco's Modified Eagle Medium (DMEM) high glucose (HG) medium with stable L-glutamine, 10% fetal calf serum (FCS). SKBR3 and were maintained in DMEM medium with F-12 modification, 2 mM stable L-glutamine, 10% FCS. SJSA1, A431 and MCF-7 cells were maintained in RPMI 1640 medium with 10% FCS.

## WST-1 assay

### Acute cytotoxicity (mCherry)

For cell viability measurements using Mitochondrial dehydrogenase (WST-1) assay 10’000 cells per well were seeded in a 96-well plate (Sarstedt, F-bottom, Item No.: 83.3924) and incubated for 24 h at 37°C, 5 % CO_2_ to settle. Cells were washed once with PBS for serum free incubation. Cells were treated with compounds at indicated concentrations together with 5 µM TNB-R_10_-ILFF in 100 µL of DMEM HG with 0 or 10% FCS at 37°C, 5 % CO_2_ for 1h. 10 µL of WST-1 reagent was added to each well and incubated for 90 min at 37 °C, 5% CO_2_. Absorbance at 460 nm was measured using a M200Pro plate reader (TECAN, Switzerland). Data was normalized to 5 µM TNB-R_10_-ILFF as 100 % viability and 0.1 % Triton-X100 as 0 % viability.

### 24h Cytotoxicity (RNase)

For cell viability measurements using Mitochondrial dehydrogenase (WST-1) assay 10’000 cells per well were seeded in a 96-well plate (Sarstedt, F-bottom, Item No.: 83.3924) and incubated for 24 h at 37 °C, 5 % CO_2_ to settle. Cells were treated with compounds at indicated concentrations together with 5 µM TNB-R_10_-ILFF in 50 µL of DMEM HG with 10 % FCS or OptiMEM 2 % FCS at 37°C, 5 % CO_2_ for 1h. 10 µL of WST-1 reagent was mixed in 40 µL DMEM HG with 10 % FCS and added to each well followed by incubation for 90 min at 37 °C, 5 % CO_2_. Absorbance at 460 nm was measured using a M200Pro plate reader (TECAN, Switzerland). Data was normalized to 5 µM TNB-R_10_-ILFF (or no treatment) as 100 % viability and 10 µM Staurosporin as 0 % viability.

## Caspase Assay

For the Caspase assay was performed according to the manufactures instructions (Caspase-Glo® 3/7 Assay System, Promega, Catalog Number G8090). 5.000 HeLa cells per well were seeded in a 96 well plate (Sarstedt, F-bottom, Item No.: 83.3924) for 48 hours at 37 °C, 5 % CO_2_. Upon treatment, the medium was replaced by 50 µL medium containing blank (medium only), TNB-R_10_-ILFF additive control (5 µM additive), positive control (1 µM Staurosporin) and RNAse (1 µM RNAse + 5 µM additive) in DMEM HG medium supplemented with 10% FCS. The cells were incubated for 24 hours at 37 °C, 5 % CO_2_. 50 µL CaspaseGlo reagent was pipetted to each well, shaken for 30 s at 300 rpm and incubated for 90 min at 37 °C, 5 % CO_2_. Luminescence was measured using a M200Pro plate reader (TECAN, Switzerland).

## Annexin V binding assay

Cells were seeded according to the specific density requirements of the cell line (e.g., HeLa-1: 5 × 10⁴ cells/mL) in a 24-well plate, ideally 48 hours prior to treatment to ensure consistency across replicates. On Day 3, 250 µL of treatment in serum-free medium was added and incubated at 37 °C with 5% CO₂ for 24 hours; controls included Staurosporin (1 µM) and untreated samples.

On Day 4, the medium containing floating cells was collected, centrifuged, and the supernatant was discarded. Adherent cells were detached by adding 200 µL Accutase, incubating for 10 minutes, and transferring the detached cells to the collection tube. The wells were washed with PBS, and the wash was collected into the same tube. The samples were centrifuged, and the cell pellet was retained on ice. The cells were washed twice with 1× Annexin V binding buffer (10 mM HEPES pH 7.4, 140 mM NaCl, 2.5 mM CaCl₂) and resuspended in 100 µL Annexin V binding buffer. Annexin V-ATTO488 (5 µL) was added, and the mixture was incubated at room temperature with gentle agitation for 15 minutes, shielded from light. After incubation, the cells were washed with 1 mL of Annexin V binding buffer, centrifuged, and the supernatant was discarded. The cells were then resuspended in 500 µL Annexin V binding buffer and kept on ice.

Immediately before flow cytometry analysis, 1 µL of propidium iodide (1 mg/mL) was added, and the mixture was gently agitated. Three control tubes were prepared: no fluorophore, Annexin V-positive/PI-negative, and Annexin V-negative/PI-positive. Flow cytometry analysis was performed, with all centrifugation steps conducted at 1100× *g* for 5 minutes at 4 °C.

Data analysis was conducted on three biologically dependent replicates from the same cell passage. The percentages of the gated populations were calculated and reported as the mean.

## Cellular uptake

HeLa cells were seeded in a black-walled 96-well glass-bottom plate with flat transparent bottom (Screenstar, microplate, F-bottom, Item No.: 655866) at 10’000 cells/well in growth medium and allowed to attach for 48 h. Cells were washed with PBS once in case of serum free incubation. 5 µM TNB-R_10_-ILFF were mixed with the indicated amount of NLS-mCherry in 100 µL growth medium with 0 or 10 % FCS for 1 hour at 37 °C, 5 % CO_2_. Treated cells were washed with prewarmed 200 µL PBS containing 0.5 mg/mL heparin. The nucleus is counterstained with Hoechst 33342 at (1:5000) in FluroBrite DMEM + 10 % FCS for 5 min. The media was replaced with fresh FluoroBrite DMEM + 10 % FCS followed by confocal microscopy.

## Microscopy

Confocal microscopy was done using a Nikon-CSU spinning disk microscope with a CSU-X1 (Yokogawa with modifications by Andor) confocal scanner unit and a live cell imaging chamber with temperature and CO_2_ control (OKOlab). Images were captured using either a PlanApo 60x NA 1.4 oil objective (Nikon) or a PlanApo 40x NA 1.4 air objective (Nikon) and an EMCCD (AU888, Andor). The automated quantification was performed using a 40x air NA 0.95. Brightfield images were acquired along with fluorescence images. Standard laser, a quad Dicroic (400-410,486-491, 560-570, 633-647, AHF) and Emission filters were used in the acquisition of confocal fluorescence images [BFP (Hoechst 33342), ex.: 405 nm em.:450/50; GFP (Atto488, mVenus), ex.: 488 em.:525/50; RFP (mCherry), ex.: 561 em.:600/50 nm.]

## SDS-PAGE gel analysis

SDS-PAGE gels were self-casted using 12 or 15 % acrylamide and run in 1x Tris-Glycine-SDS buffer (Carl Roth, Germany) at 180 V. Samples were diluted in water or PBS and supplemented by 1x Laemmli buffer (Bio-Rad, USA) with (reducing, ‘+’) or without (non-reducing, ‘-‘) the addition of β-mercaptoethanol (ACROS Organics) and heated for 10 min at 95 °C. In case of the native SDS-PAGE, protein was not heat denatured at 95 °C. The proteins were stained with Blue stain (FastGene) for at least 2 h and destained with water. The images were acquired using a ChemiDoc^TM^ Imager (Bio-Rad, USA).

## Endosome rupture assay

HeLa cells were seeded into a 60 mm cell culture dish (2 × 10^6^ cells/dish) in DMEM High Glucose Medium supplemented with 10 % FCS and allowed to attach overnight. The next day, cells were transfected using 10 µg pEGFP-hGal3 (a gift from Tamotsu Yoshimori (Addgene plasmid # 73080; http://n2t.net/addgene:73080; RRID: Addgene_73080)) using Lipofectamin 3000 and P3000 Reagent following manufactures protocol for transient transfection. After 6 hours, transfected cells were harvested and seeded into 8-well microscopy (300 µL, 1 × 10^5^ cells/mL) slides. After 2 days, cells were treated using BioRAM-protein (2.5 µM in 10 % serum and 1 µM in 0% serum) in presence of 5 µM CPP-additive) or LLOMe (1 mM) as positive control for 30 min. Cells were washed twice using heparin in PBS and imaged in FluoroBrite DMEM supplemented with 10 % FCS.

## RNase activity assay

For assessment of RNase activity 1 mg/mL yeast RNA was incubated with a stock of 40x SYBR-safe (1:1) in 2.5x volume PBS (pH 7.4) and incubated for 10 min at room temperature. 20 µL RNase was added to 80 µL of the RNA+SYBR-safe mixture. Measurement was immediately started using a M200Pro plate reader (TECAN, Switzerland) with excitation at 280 nm and emission at 530 nm over a total measurement time of 15 min. The data was corrected by only RNA+SYBR-safe in PBS, accounting for background degradation by RNase traces. Michaelis-Menten parameters were calculated using Graphpad Prism 10 (GraphPad Software, USA).

## Intranuclear fluorescence intensity measurement.

### Software

Microscopy images were processed with ImageJ including the FIJI package.^[8]^ Graphing and statistics were done using Graphpad Prism Version 10.3.1 (509) (GraphPad Software, USA).

### Quantification script

The aim is to measure and compare the amount of fluorescent protein within the nucleus. The following script enables automated processing of dual channel images (fluorescent protein, nuclear co-stain), limiting the measurement to the nuclear area and excluding bright endosomes and aggregates, thereby reducing the measured fluorescence intensity to the disperse nuclear signal. (**Note:** This is an approximation optimized for batch processing and only displaying relative values. This has to be taken into consideration for later data analysis and deduction.)

The automation is run using the software NIS 5.42.04 and the JOBs module (Nikon) and uses the software autofocus to capture randomly generated positions within a well (borders excluded), in case it detects at least 4 nuclei. Acquisition for the well is stopped after nuclei count of 200.

The ImageJ script for image analysis is performs the following technical steps for every image taken:

1. Dissecting cell nuclei using nuclear stain (Hoechst, BFP channel) and transforming every nucleus into a mask. Nuclei touching the border of the image are disregarded.
2. Identifying the brightest spots (aggregates and endosomes) based on a manually set threshold, inverting the selection (everything that is not an endosome/aggregate) and transforming them into another mask
3. Combination of the nuclear mask and the inverted endosome mask
4. Measuring selected parameters for the combined mask

The output of this script is a table for every single nucleus, displaying the prior set parameters (e.g. area, integrated density, mean gray value, median, …). To analyze the disperse signal we decided to use only the median fluorescence, considering that some endosomes are not captured by the threshold. The median is normalized to the area measured and takes the distribution of values into account and thereby represents the disperse nuclear signal best. The script also applies for covalently conjugated protein (NLS-mCherry **L**), which is accumulating in the nucleoli inside the nucleus, as long as the signal threshold for endosome detection is not reached.

For further data analysis, the median values for every nucleus were combined for every single well, yielding between 130-200 values, representing one well. Subsequently the mean of every well was calculated and combined with the mean of the other two replicates, representing one full dataset, displayed in the graphs. Experimental results are dependent on the execution (incubation time, washing, imaging set up) and are therefore normalized to controls within one experiment, yielding the relative fluorescence intensity for the respective treatments.

**Code:**

//setBatchMode(true);

//Remove the comment sign from the line above if you don't want to see the open images

//choose the directory for the raw image files

dir=getDirectory("Choose Source Directory ");

list = getFileList(dir);

for(i=0; i<list.length; i++){

file=list[i];

run("Bio-Formats Importer", "open=["+dir+""+file+"] autoscale color_mode=Default concatenate_series open_all_series rois_import=[ROI manager] view=Hyperstack stack_order=XYCZT");

getDimensions(width, height, channels, slices, frames);

// starting with the opening of images that are saved as stacked files for every well, consiting out of 2 channels

for(f=1; f<=frames; f++){

Stack.setFrame(f);

name=getTitle;

nameseries=name+"_"+ f;

//analysis of the Hoechst channel (1), identifying the nuclei, excluding nuclei that are on the edges of the image

run("Duplicate...", "duplicate channels=1 frames=f");

run("Median...", "radius=5");

setAutoThreshold("Huang dark no-reset");

run("Convert to Mask");

run("Fill Holes");

run("Analyze Particles...", "size=20-Infinity circularity=0.20-1.00 show=Outlines exclude add");

//opening the RFP channel (2)

selectWindow(name);

run("Duplicate...", "duplicate channels=2 frames=f");

//Endosome-Signal selection is based on a threshold, that is set manually and should be carefully checked for different conditions (oriented at qualitatively highest uptake)

setThreshold(8000, 65535, "raw");

run("Create Selection");

run("Make Inverse");

roiManager("Add");

//Get the total number of elements in ROI manager

roiCount = roiManager("count");

//Define the index for last ROI

nonEndosomeROI = roiCount-1;

//Rename the endsomeROI

roiManager("Select", nonEndosomeROI);

roiManager("Rename", "nonEndosomeROI");

//Create a loop for taking the intersection of each nucleus ROI with the nonEndosome ROI and reorganize the ROI manager

for (j=0; j<nonEndosomeROI; j++) {

//Select the first ROI and the nonEndosomeROI

roiManager("Select", newArray(0,nonEndosomeROI-j));

//Take their intersection, make a new ROI, add it to the end of ROI manager

roiManager("AND");

//excluding "no selections" by testing for selection type, that is -1 in case of "no selections" (quality control step, to keep the script running for the case that no selection could be made)

type=selectionType();

if (type==-1) {

print("null");

} else {

roiManager("add");

}

//Delete the very first ROI which has already been modified

roiManager("Deselect");

roiManager("Select", 0);

roiManager("Delete");

}

//When all the nucleus ROIs are modified, we can delete the nonEndosomeROI (which is now at the top of the list)

roiManager("Select", 0);

roiManager("Delete");

//Measure all the intensity values of all new ROIs

run("Select All");

roiManager("Show All");

roiManager("Measure");

dir2=dir+"/insert directory here/";

File.makeDirectory(dir2);

//roiManager("Deselect"); in case the ROIs should be saved

//roiManager("save", dir2 + name +".zip");

//Refresh the ROI manager, close all open images, go to the next image in the input directory

roiManager("Delete");

selectWindow(name);

close("\\Others");

}

saveAs("Results", dir2 + name + "_Results.csv");

run("Clear Results");

}

# Peptide Synthesis

## Peptide Synthesis and modifications

Peptides were synthesized by Fluorenylmethoxycarbonyl (Fmoc)-solid-phase peptide synthesis (SPPS) using a peptide synthesizer (Automated microwave peptide synthesizer, CEM) on TentaGel S Ram resin (RappPolymere, Tübingen, Germany, 0.05-0.1 mmol scale, 0.22 mmol/g). Couplings were achieved by reacting 0.2 M Fmoc-AA-OH with 0.25 M DIC and 0.25 M Oxyma in DMF. A solution of 20% Piperidine in DMF was used to remove the Fmoc protection group. The acetylation is carried out with 3.5 ml DMF, 1 ml acetic anhydride and 0.5 ml diisopropylamine (DIPEA) overnight at room temperature. Arginine was incorporated with Pbf protection using double couplings, cysteine was incorporated on the *N*-termini with Trityl, NPYS or S^t^Bu protection, in case activation of the disulfide was performed on resin.

## Peptide final deprotection and cleavage, purification, and characterization

Peptides were deprotected and cleaved from the resin using a mixture of 10 mL TFA, 0.75 g phenol, 0.5 mL water, 0.5 mL methylphenylsulfide, and 0.25 mL 1,2-ethandithiol. After 3 h at room temperature, the cleavage solution was collected, and the crude peptides were precipitated from ice-cold ^t^Butyl-methyl ether. Crude peptides were washed five times with dry diethyl ether. Final deprotection was done by adding 95:2.5:2.5 of TFA/TIS/H_2_O overnight at 25 ºC. Peptides were purified by Preparative reverse phase-high performance liquid chromatography (RP-HPLC) on a Shimadzu system using a VP 250/21Nucleodur C18 Htec column (Macherey-Nagel, 100 A, 5 µm, 250 mm x 21 mm, 10 mL/min). The following gradient was used in all purifications: A = H_2_O + 0.1 % TFA, B = acetonitrile (MeCN) + 0.1% TFA 5 % B 0-5 min, 5-95 % B 5-60 min, 95 % 63-70 min. UPLC-UV traces were obtained on a Waters H-class instrument equipped with a Quaternary Solvent Manager, a Waters autosampler and a Waters TUV detector with an Acquity UPLC-BEH C18 1.7 μm, 2.1x 50 mm RP column. The Empower 3 software (Waters) was used. The following gradient was used: A = H_2_O + 0.1 % TFA, B = MeCN + 0.1 % TFA 5-95 % B 0-5 min, flow rate 0.6 mL/min. UPLC-UV chromatograms were recorded at 220 nm. Masses were measured using a Xevo G2-XS QTof (Waters) high-resolution mass spectrometer coupled to an Acquity UPLC system running on water and acetonitrile, both with 0.01% formic acid using the MassLynx software (V4.1, Waters).

|  | **Peptide** | **Sequence** | **remark** | **reference** |
| --- | --- | --- | --- | --- |
| 1 | TNB-R_10_-ILFF (“additive”) | NH_2_-C(TNB)-Peg-Peg-RRRRRRRRRR-ILFF-CONH_2_ | a | Arafiles et al. ^[2]^ |
| 2 | Cys-R_10_ | NH_2_-C-Peg-Peg-RRRRRRRRR-CONH_2_ | a | Schneider et al. ^[1]^ |
| 3 | Maleimid-R_10_ | Maleimidoacetic acid-Peg-Peg-RRRRRRRRRR-CONH_2_ | b | Schneider et al. ^[1]^ |
| 4 | 3b-R_4_ | ac-C(2-Mp-4-Np-carbonate)-Peg-RRRR-CONH_2_ | c | - |
| 5 | 3b-R_6_ | ac-C(2-Mp-4-Np-carbonate)-Peg-RRRRRR-CONH_2_ | c | - |
| 6 | 3b-R_8_ | ac-C(2-Mp-4-Np-carbonate)-Peg-RRRRRRRR-CONH_2_ | c | - |
| 7 | 3b-R_10_ | ac-C(2-Mp-4-Np-carbonate)-Peg-Peg-RRRRRRRRRR-CONH_2_ | c | - |
| 8 | 3a-R_10_ | ac-C(2-Me-4-Np-carbonate)-Peg-Peg-RRRRRRRRRR-CONH_2_ | c | - |
| 9 | 3a-R_10_ (F) | TAMRA-G-RRRRRRRRRR-C(2-Me-4-Np-carbonate)-CONH_2_ | b | - |
| 10 | 3b-R_10_ (F) | TAMRA-G-RRRRRRRRRR-C(2-Mp-4-Np-carbonate)-CONH_2_ | b | - |
| 11 | NPYS-R_10_ | C(NPYS)-Peg-Peg-RRRRRRRRR-CONH_2_ |  | - |

Peg = 8-amino-3,6-dioxaoctanoic acid.

(2-Me-4-Np-carbonate) = 2-mercaptoethyl (4-nitrophenyl) carbonate (*via disulfide)*

(2-Mp-4-Np-carbonate) = 2-mercaptopropyl (4-nitrophenyl) carbonate (*via disulfide)*

TAMRA= 5-Carboxytetramethylrohodamine

ac = acetylated *N*-terminus

a)

The activation of Cys-R_10_-ILFF to TNB-R_10_-ILFF was performed on resin before cleavage instead of in solution. Therefore Fmoc-Cys(S^t^Bu)-COOH building block was used during microwave synthesis. After Fmoc-deprotection, the S^t^Bu protecting group was removed using 20 % β-mercaptoethanol in DMF at room temperature overnight. After thorough washing, cysteine activation was performed using Ellman´s reagent (5,5-dithio-bis-(2-nitrobenzoic acid)) in 25 % ethanol in DMF. Cleavage was performed using only TFA/TIS/H_2_O (95:2.5:2.5).

b)

TAMRA and Maleimidoacetic acid were conjugated on resin to the free *N*-terminus using 3 equiv. of HATU, 10 equiv. of DIPEA with 3 equiv. of the respective acid in DMF. The resin was incubated with the reaction mixture for 2 hours at room temperature. After washing with 3xDMF and 3xDCM, cleavage was conducted following the general protocol.

c)

Peptides 4-10 were synthesized with free cysteine using standard SPPS conditions, purified and further derivatized.

## General Synthesis of Peptide-Linker

The cysteine containing peptide (1 equiv., 6 µmol) and the respective linker **2** (1.5 equiv., 9 µmol) was dissolved in a 2 mL MeCN/buffer (4:1) mixture (0.1 M potassium phosphate pH = 4.5). and stirred for 2-4 hours at 20 °C. Successful conversion is indicated by yellow color change of the colorless solution. The mixture was lyophilized overnight. Solubilization in water and filtration yields the unreacted linker as precipitate and the product in solution. Reverse phase HPLC purification yielded the pure product. Yield refers to the purified peptide as starting material.

**8b-R_4_**

Isolated yield: 10.6 mg (4×TFA salt) (5.05 µmol 67 %) starting from 7.5 µmol

**
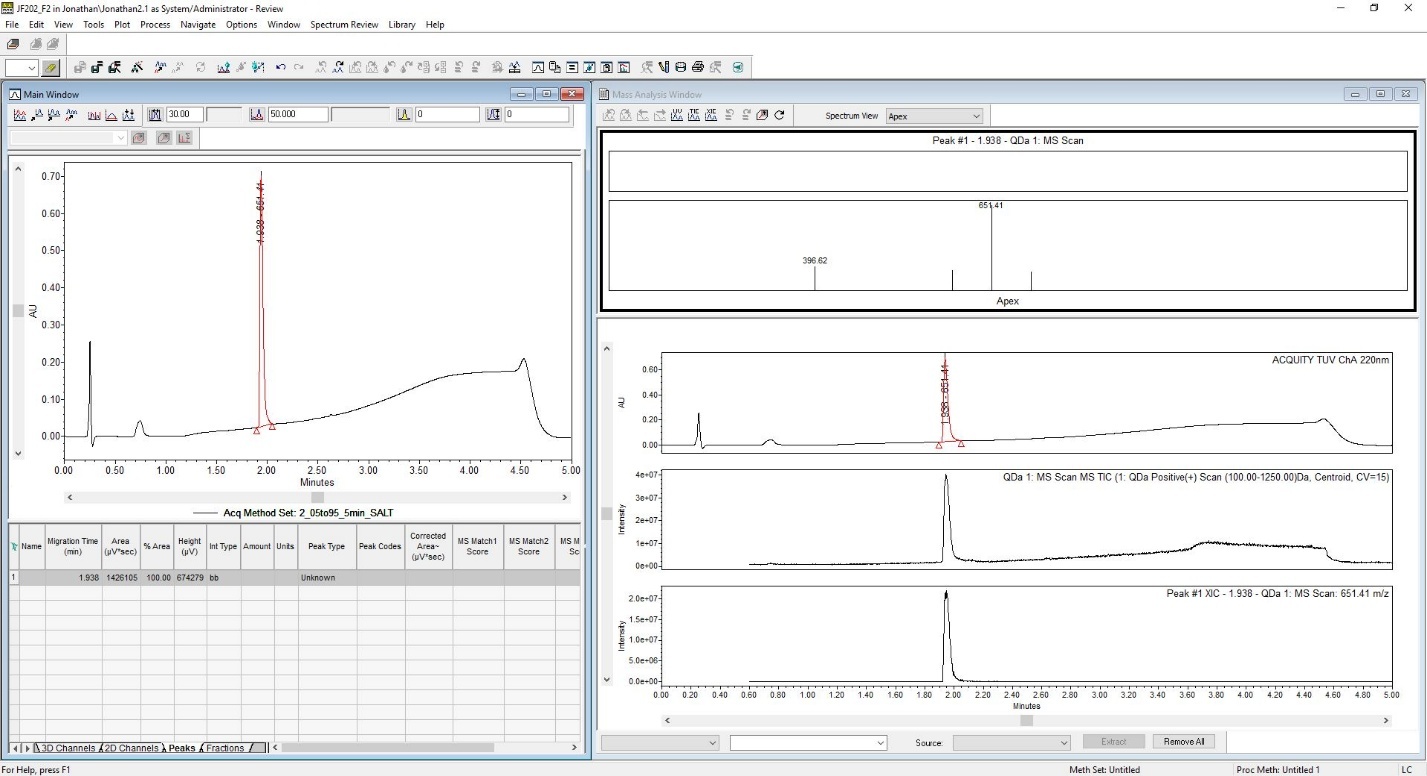

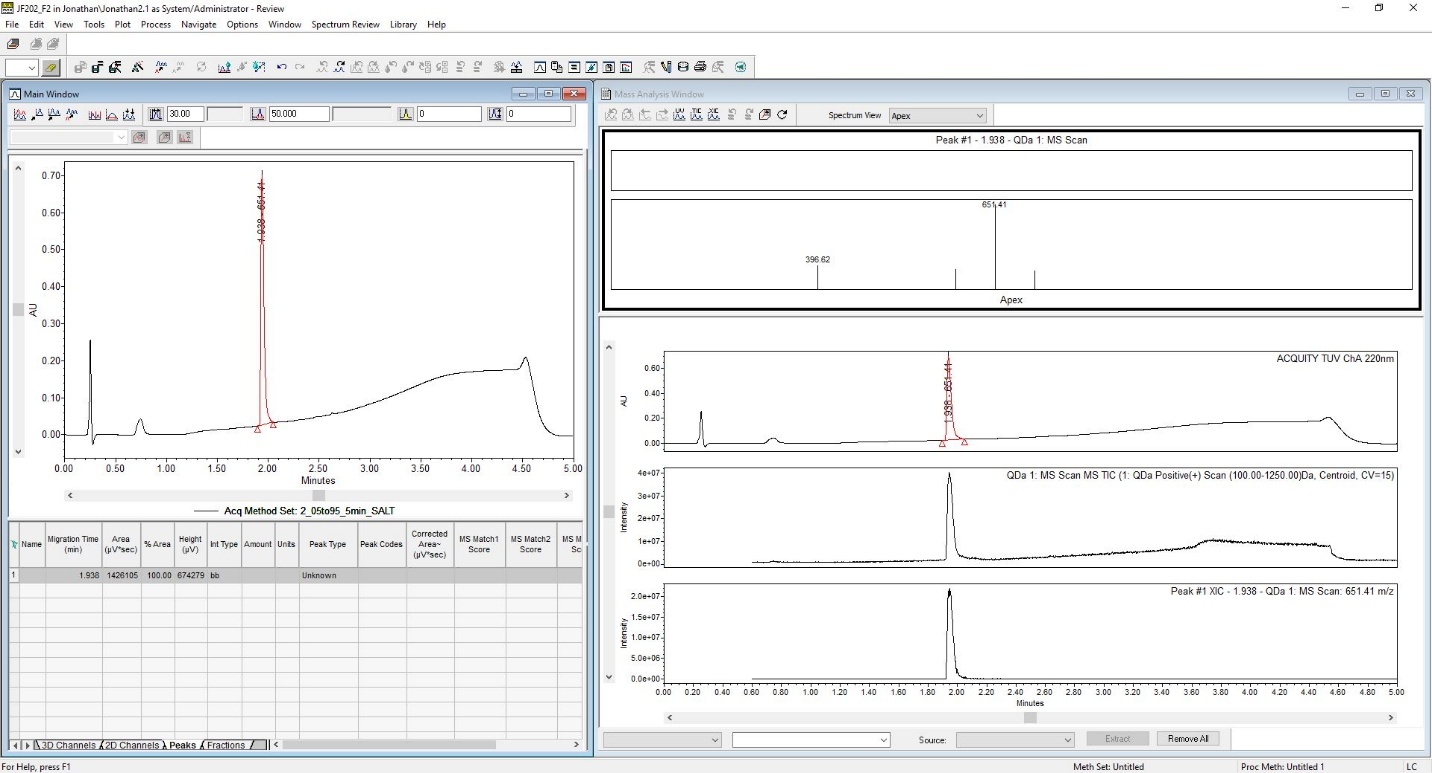
HR-MS (ESI)** *m*/*z* calcd. for C_45_H_80_N_20_O_14_S_2_^2+^: 594.2797 [M+2H^+^]; found: 594.2843.

**8b-R_6_**

Isolated yield: 12.3 mg (6×TFA salt) (4.29 µmol 46 %) starting from 9.4 µmol


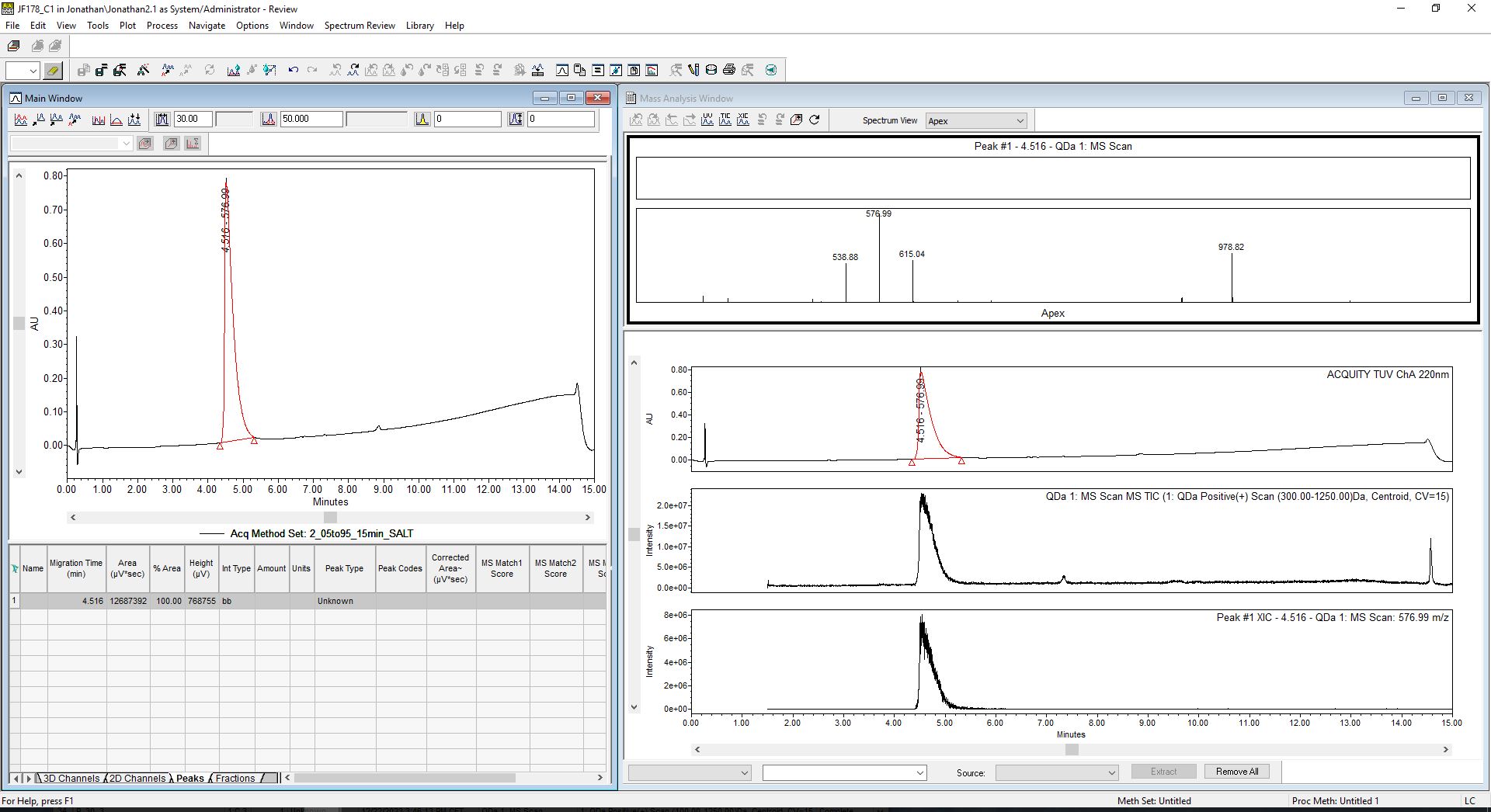

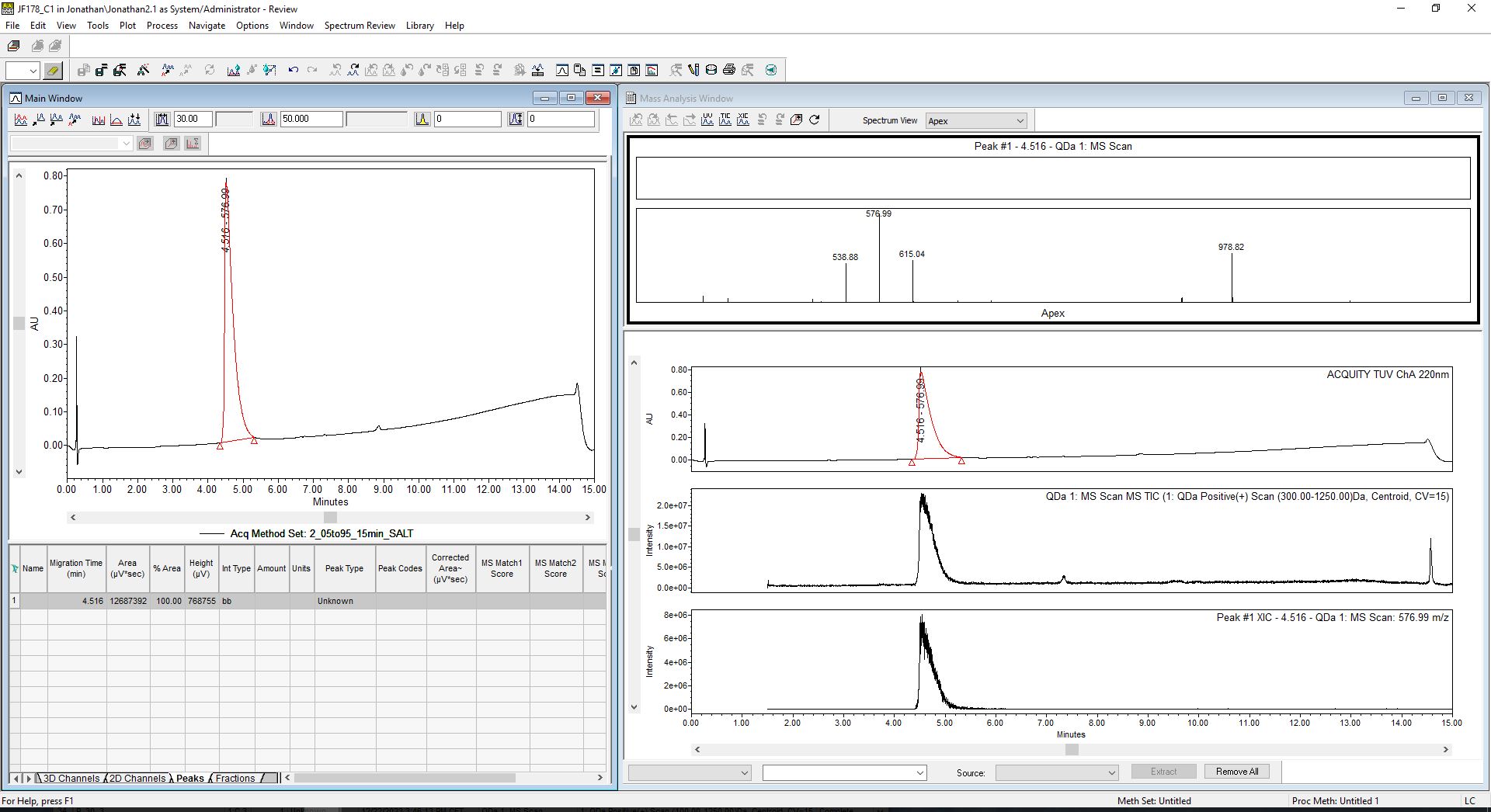
**HR-MS (ESI)** *m*/*z* calcd. for C_57_H_104_N_28_O_16_S_2_^2+^: 750.3808 [M+2H^+^]; found: 750.3861.

**8b-R_8_**

Isolated yield: 17.9 mg (8×TFA salt) (4.92 µmol 51 %) starting from 9.7 µmol

**HR-MS (ESI)** *m*/*z* calcd. for C_69_H_129_N_36_O_18_S_2_^3+^: 604.6570 [M+3H^+^]; found: 604.6517.


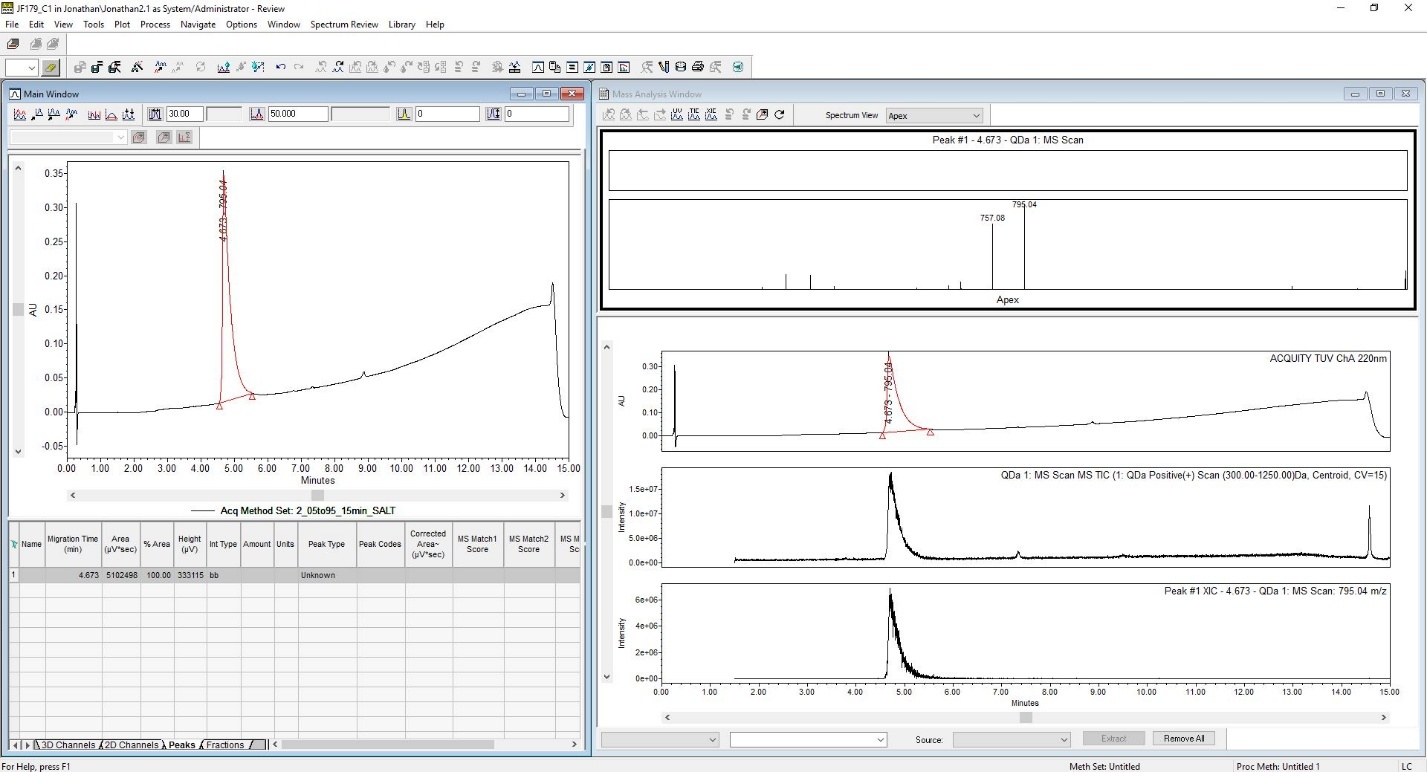

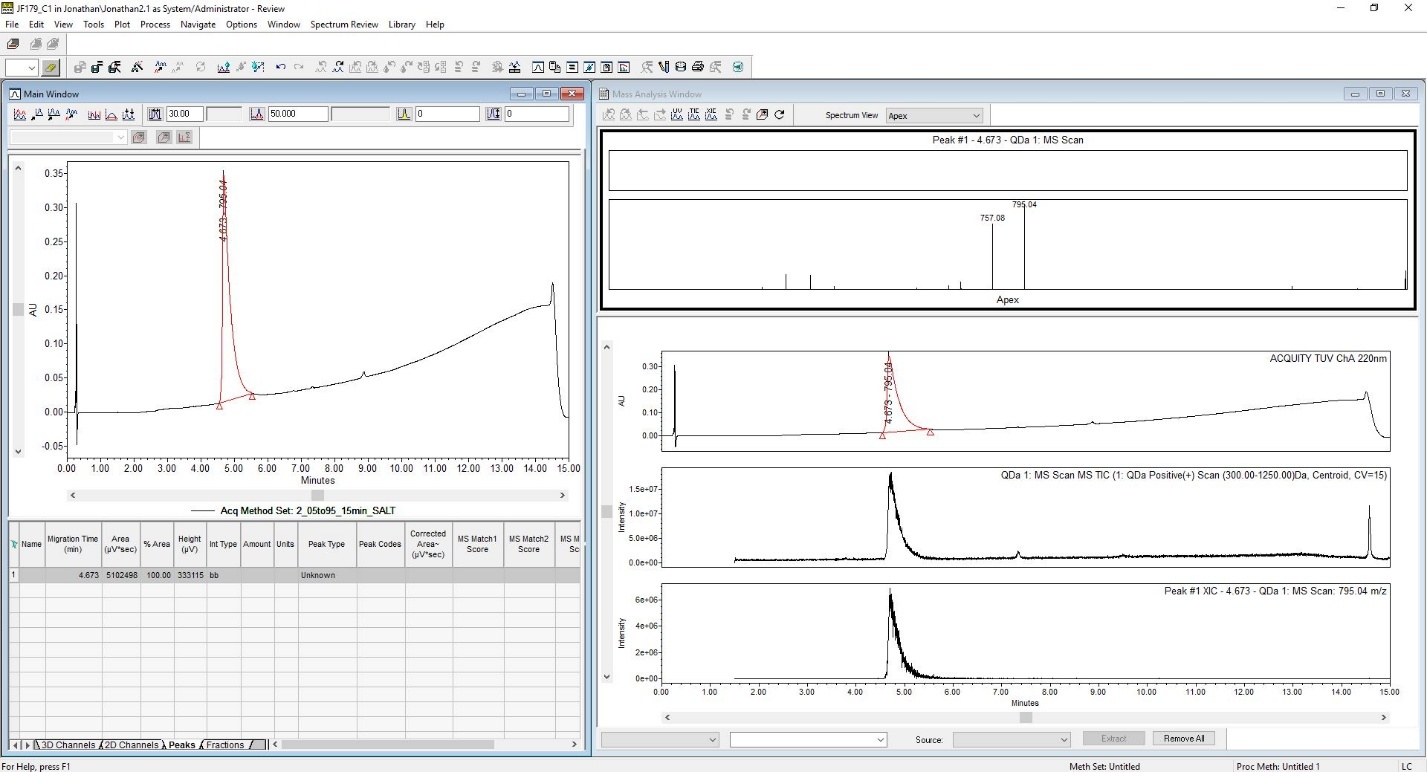


**8b-R_10_**

Isolated yield: 12.1 mg (10×TFA salt) (3.55 µmol 59 %) starting from 6 µmol

**
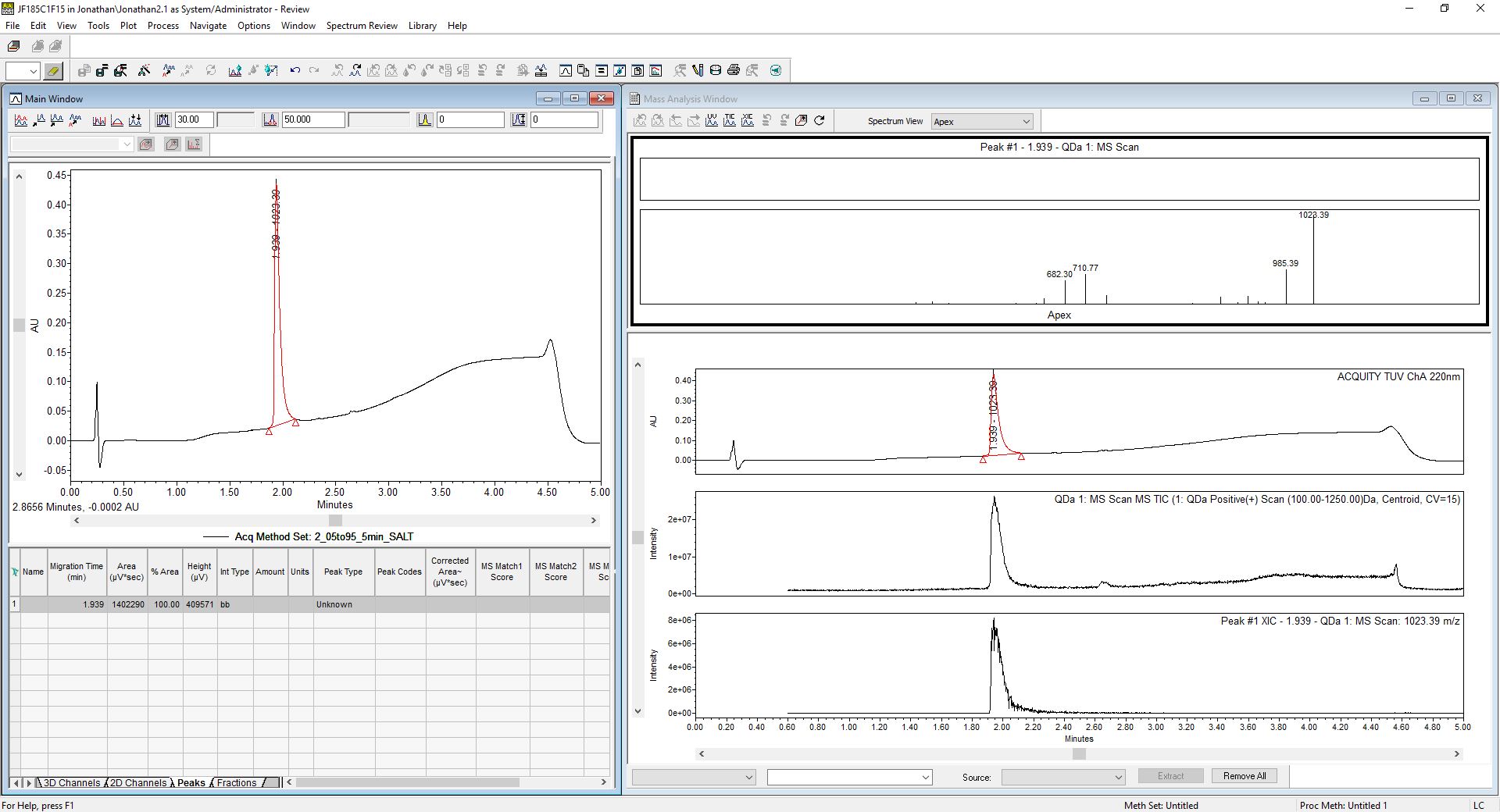

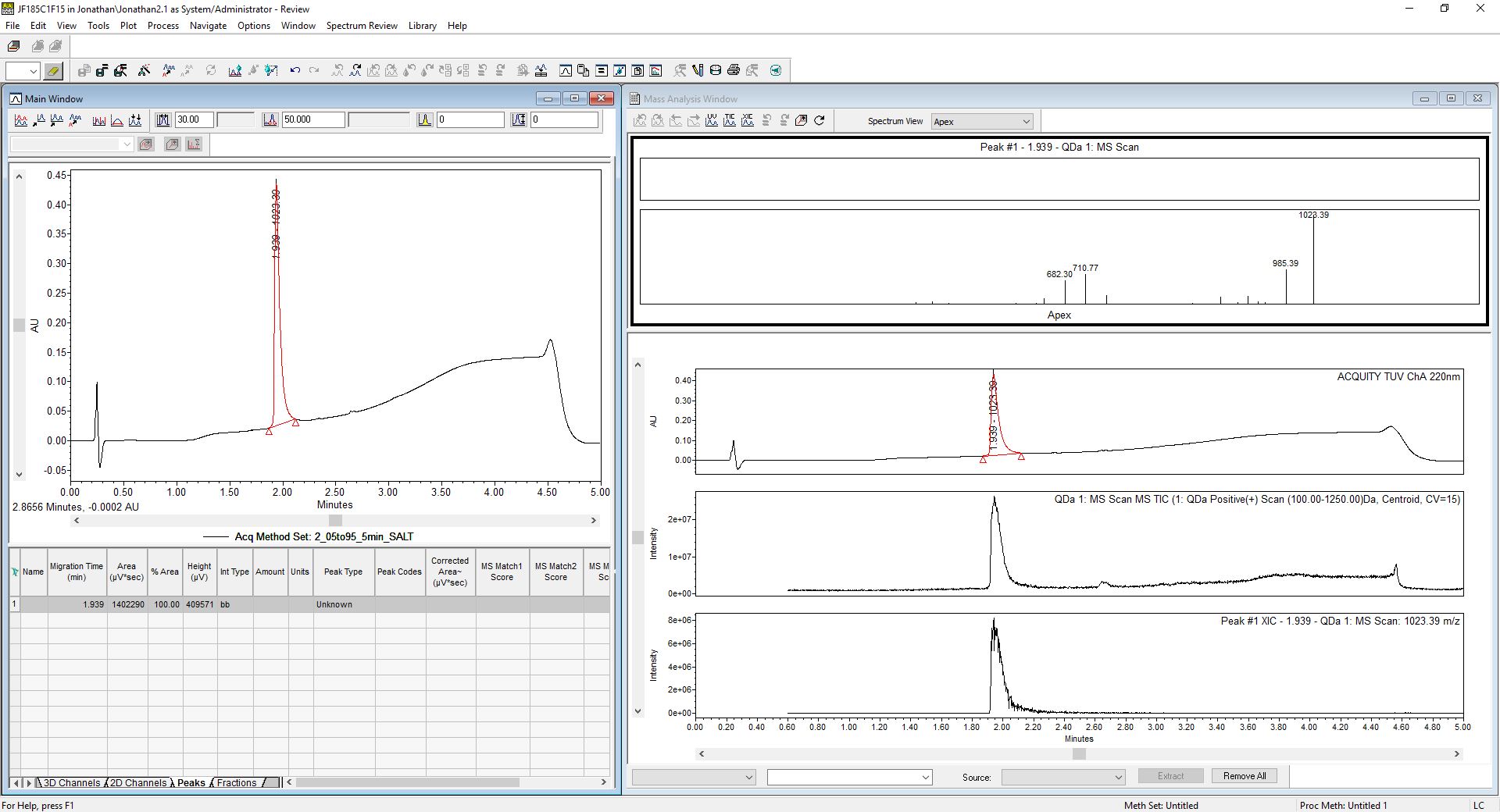
HR-MS (ESI)***m*/*z* calcd. for C_87_H_165_N_45_O_23_S_2_^4+^: 568.0651 [M+4H^+^]; found: 568.0636.

**8a-R_10_**

Isolated yield: 7.56 mg (10×TFA salt) (2.23 µmol 81 %) starting from 2.77 µmol

**HR-MS (ESI)***m*/*z* calcd. for C_86_H_163_N_45_O_23_S_2_^4+^: 564.5597 [M+4H^+^]; found: 564.5621.


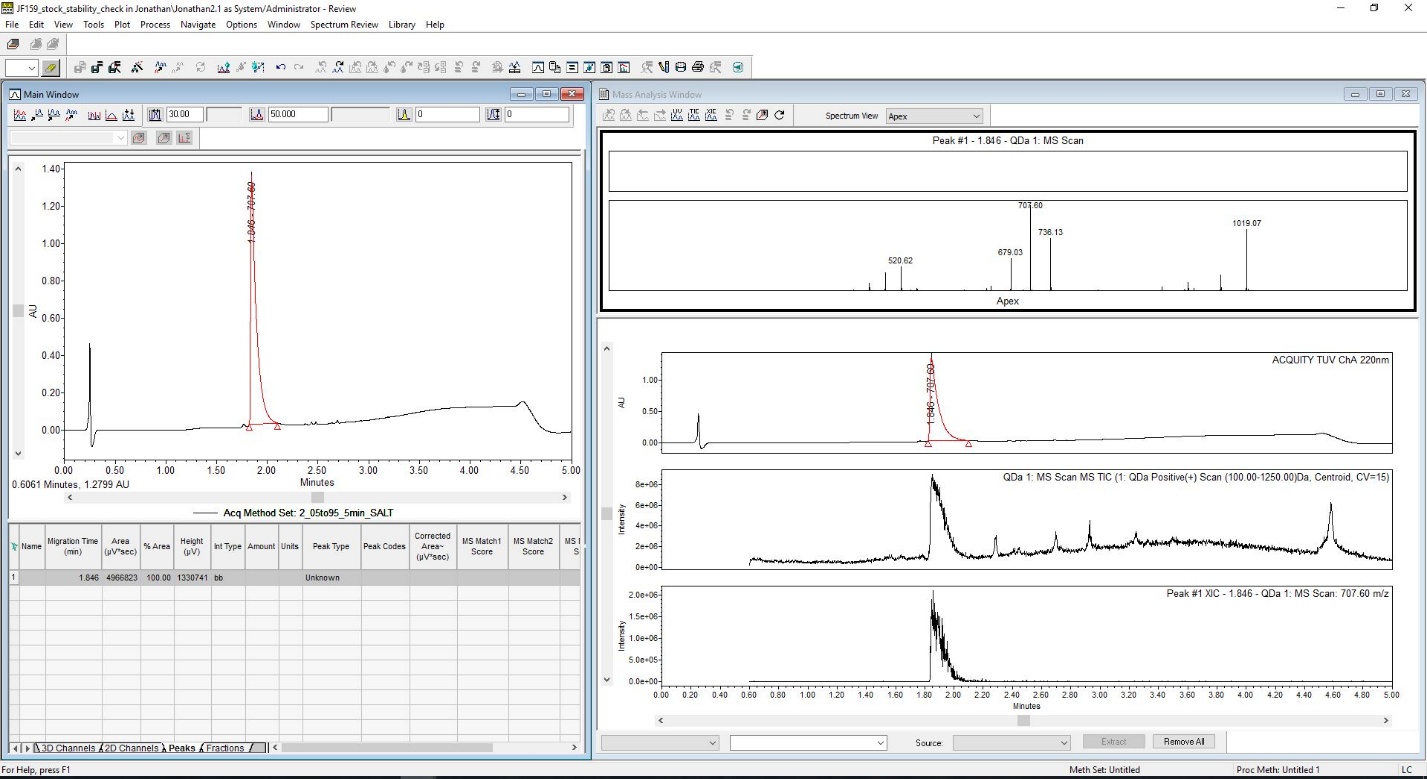


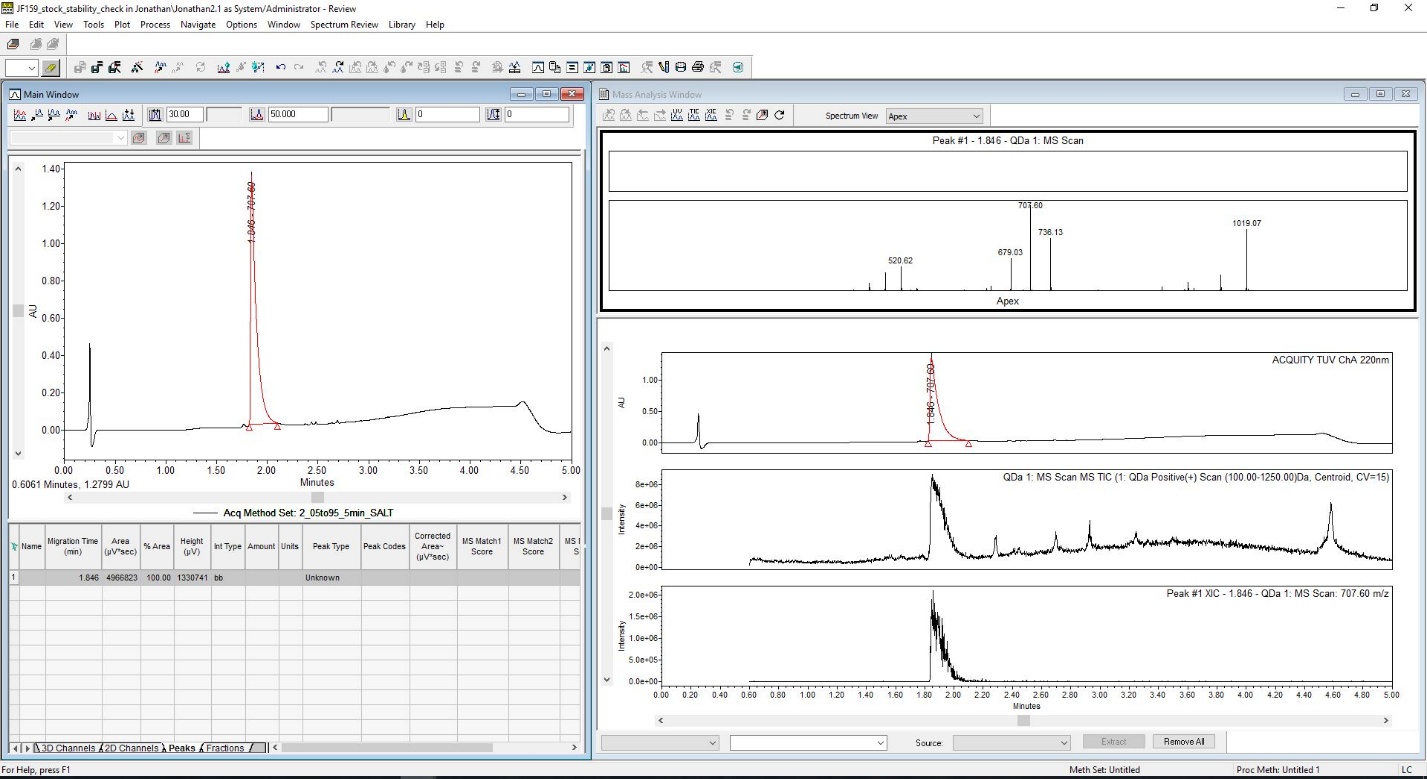


**3a-R_10_(F)**

Isolated yield: 3.84 mg (10×TFA salt) (1.09 µmol 34 %) starting from 3.19 µmol.

**HR-MS (ESI)** *m*/*z* calcd. for C_100_H_165_N_45_O_21_S_2_^6+^: 399.7104 [M+6H^+^]; found: 399.7088.


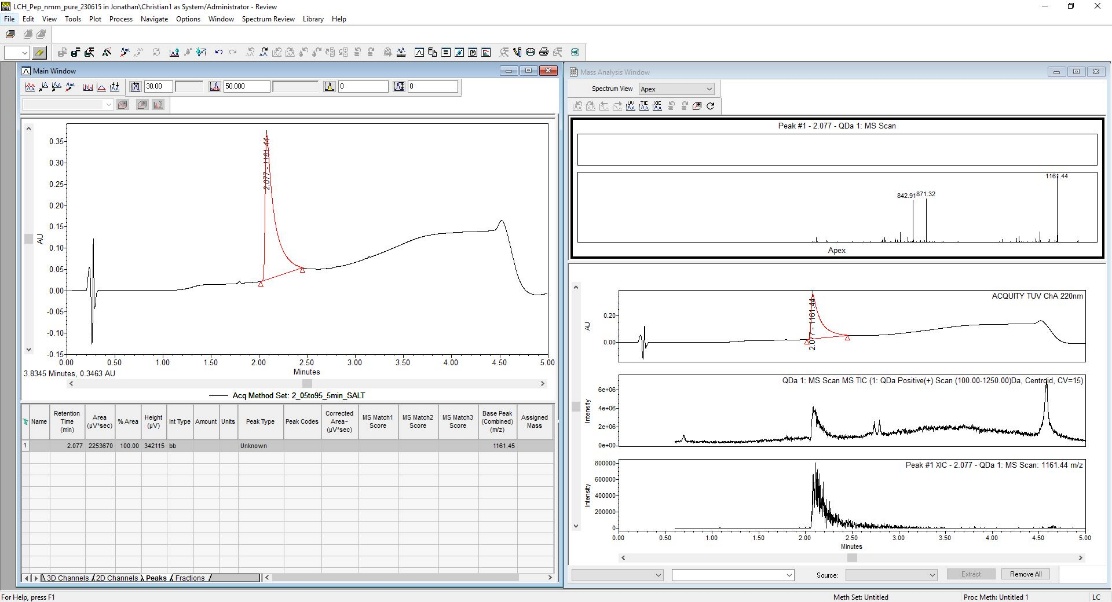


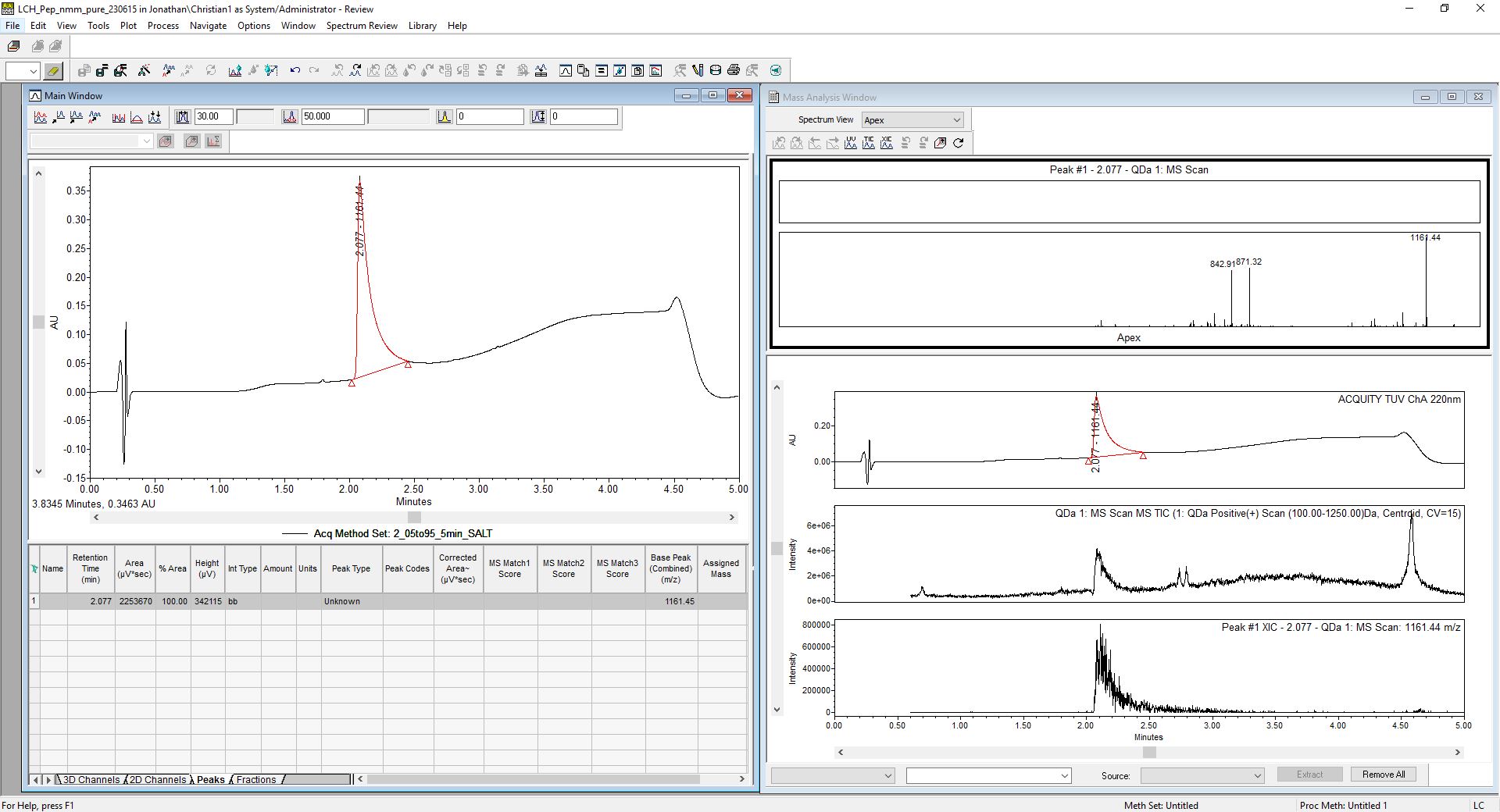


**3b-R_10_ (F)**

Isolated yield: 2.51 mg (10×TFA salt) (0.71 µmol 23 %) starting from 3.07 µmol.

**HR-MS (ESI)** *m*/*z* calcd. for C_101_H_167_N_45_O_21_S_2_^6+^: 402.0463 [M+6H^+^]; found: 402.0436.


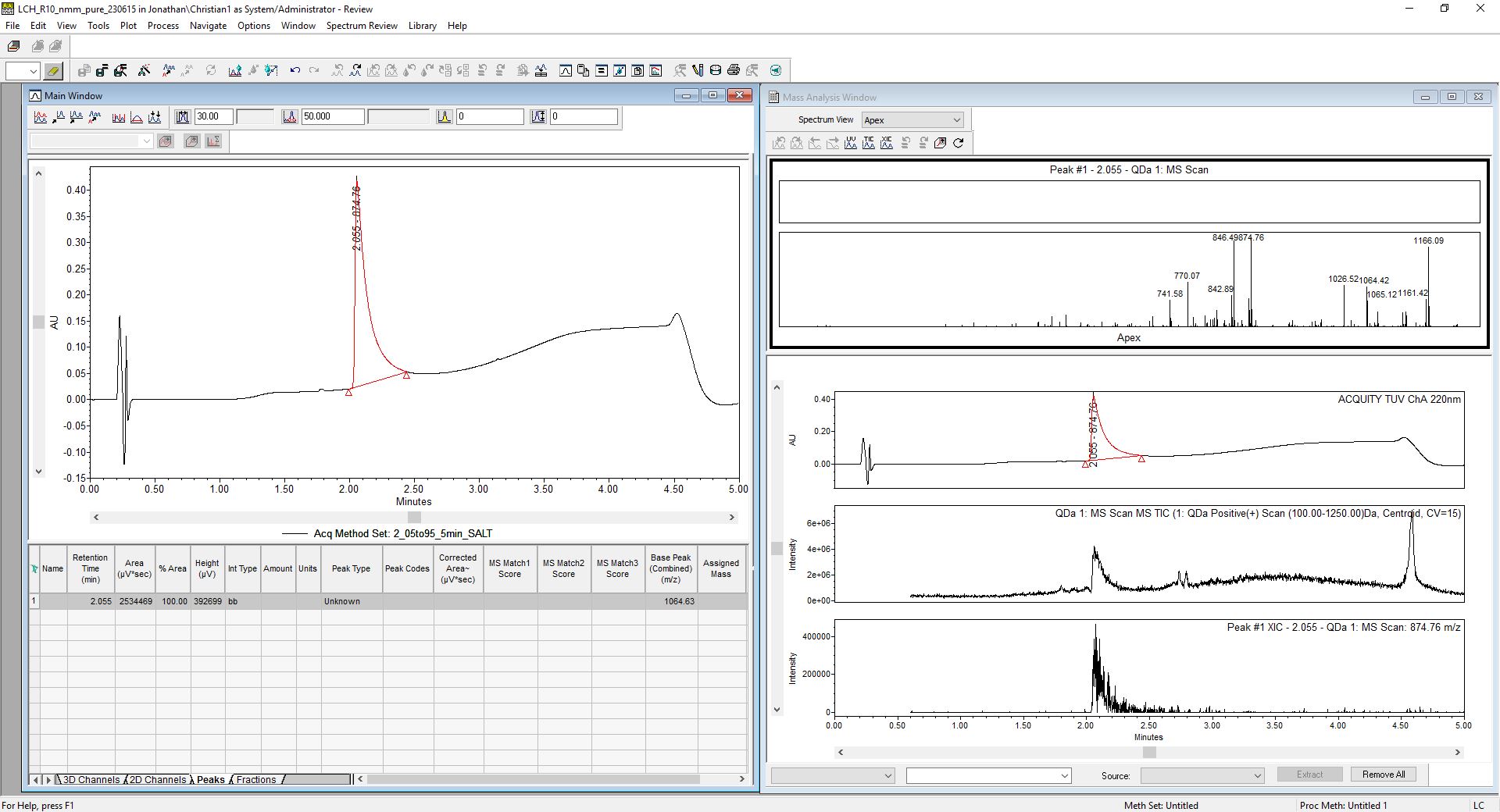


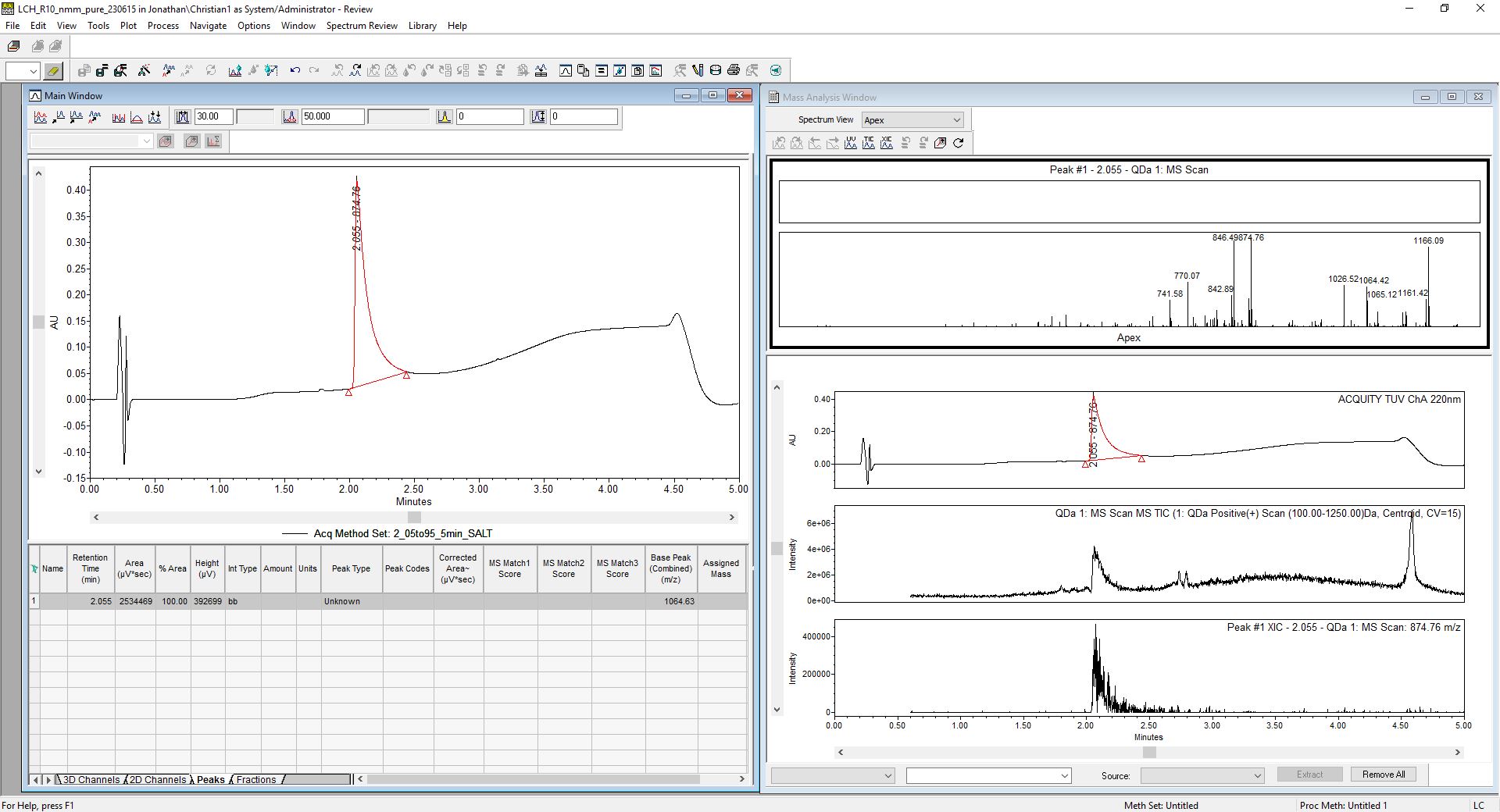


**NPYS-R_10_**

Isolated yield: 38 mg (10×TFA salt) (12 µmol 12 %) starting from 100 µmol on resin.

**HR-MS (ESI)** *m*/*z* calcd. for C_80_H_158_N_46_O_19_S_2_^6+^: 355.2037 [M+6H^+^]; found: 355.1995.


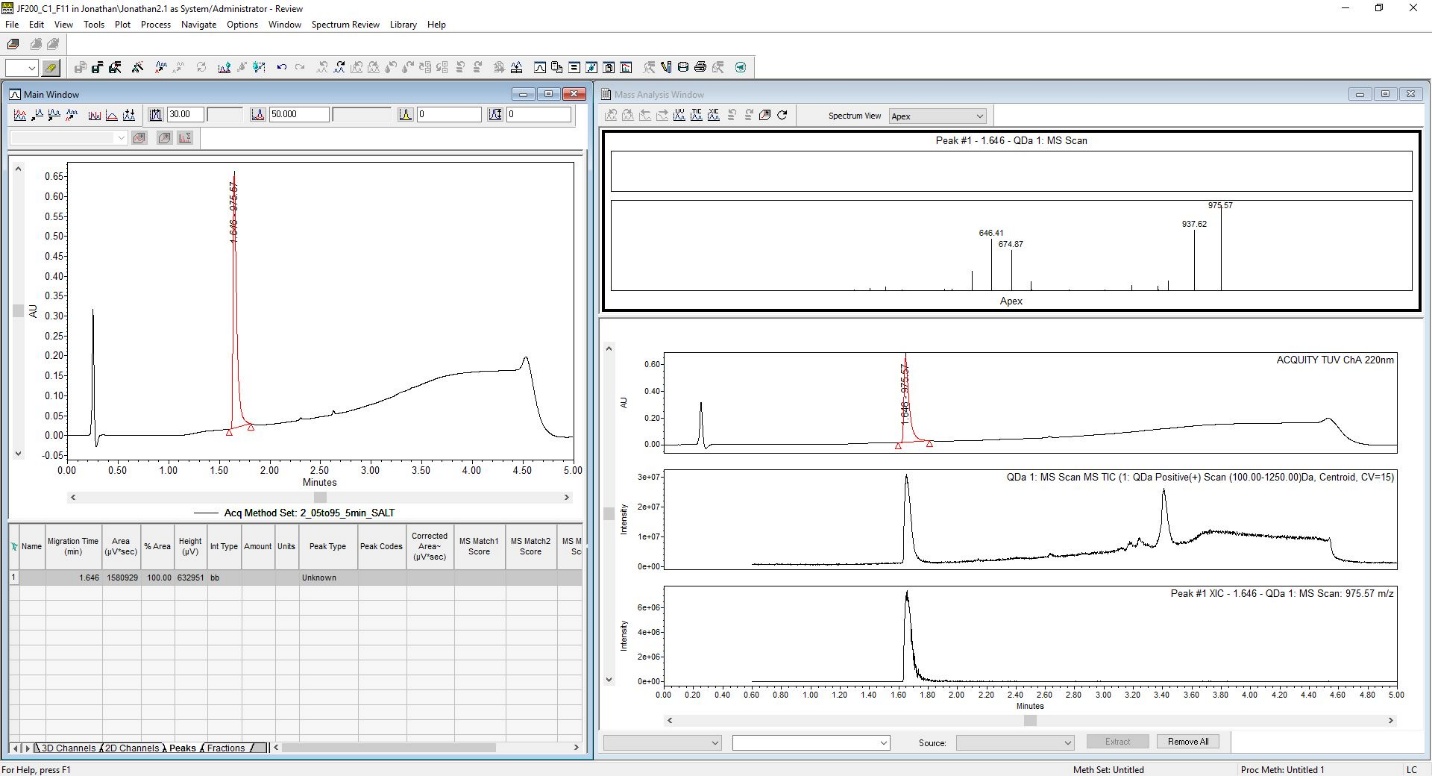


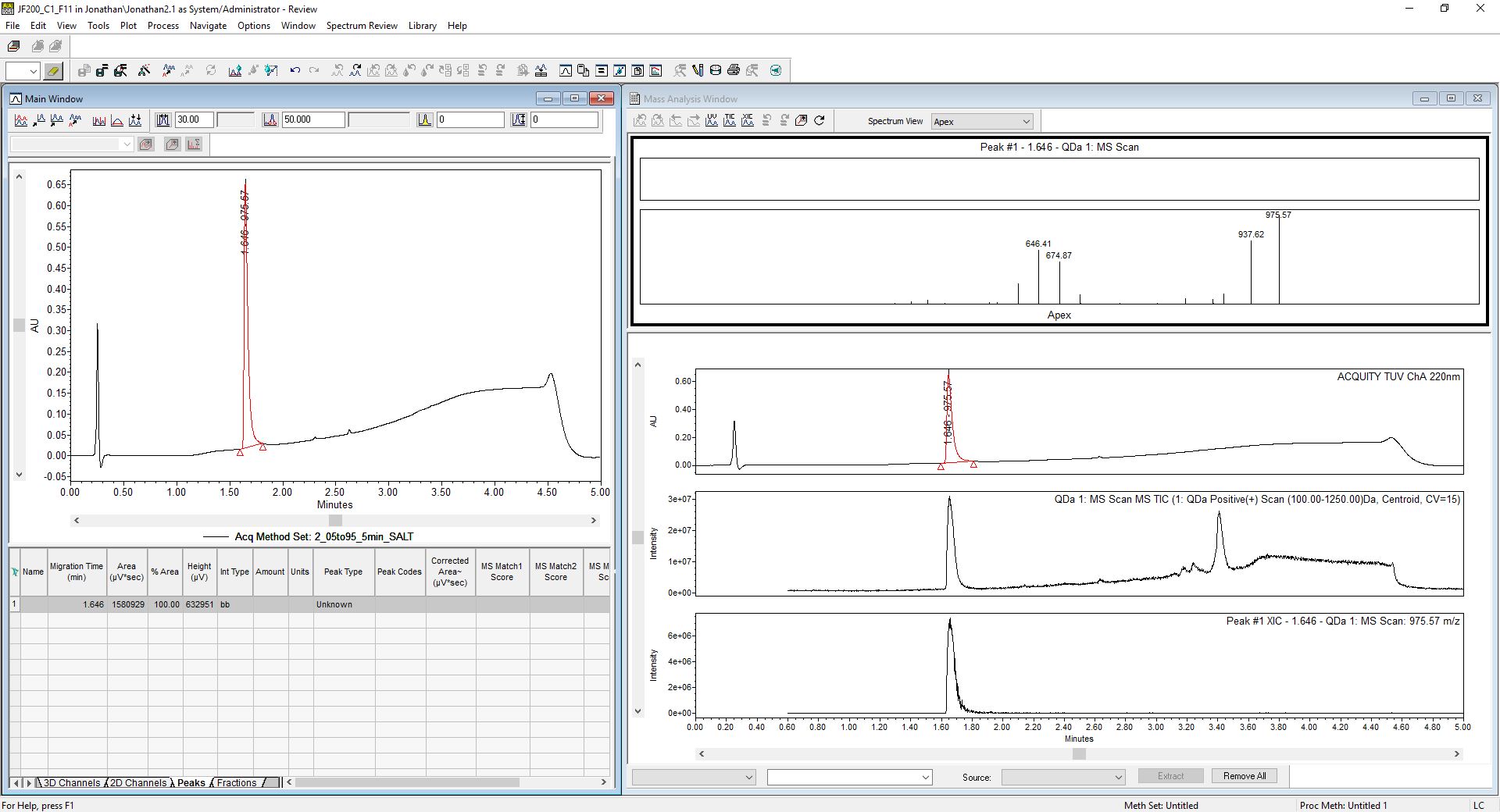


# Organic Synthesis

### 1c – 2-mercaptopropan-1-ol

A solution of ethyl 2-bromopropionate **SI-1b** (10.0 g, 1.0 equiv., 55.0 mmol) and potassium thioacetate (8.2 g, 1.3 equiv., 72 mmol) in DMF (100mL) was stirred at 20 °C for 2 h. The reaction mixture was added to water (100 mL) and extracted using methyl tert-butyl ether (MTBE) (3x100 mL). The combined organic layers were dried over Na_2_SO_4_, filtered, and were concentrated. Purification of the concentrated residue was performed by column chromatography on silica gel (starting with 1 % → 10 % ethyl acetate in hexane) yielding ethyl 2-(acetylthio) propanoate **SI-2b** (8.7 g, 89 %) as a yellow oil.

**SI-2b: UPLC-MS (ESI)** *m/z* calcd. for C_7_H_12_O_3_S^+:^ 199.04 [*M*+Na]^+^; found: 199.06 [*M*+Na]^+^.

ethyl 2-(acetylthio)propanoate **SI-2b** (8.68 g, 1.0 equiv., 49.3 mmol) was dissolved in dry THF (100 mL) and added dropwise to a suspension of LiAlH_4_ (3.74g, 2 equiv., 98.5 mmol) in dry THF at 0 °C. The resulting mixture was allowed to warm up to room temperature and further heated to 75 °C for 2 hours, after which TLC analysis (50 % ethyl acetate in hexane, R*_f, product_* = 0.75) confirmed formation of product. The mixture was then cooled to 0 °C followed by the addition of 40 mL ethyl acetate and aqueous HCl (2M, 20 mL). The mixture was extracted with ethyl acetate (3x 50 mL). Combined organic layers were washed with brine (50 mL), dried over Na_2_SO_4_ and filtered. Concentration under reduced pressure yielded an enantiomeric mixture of crude **1b**, which was used without further purification (3.2 g, 70 %). The NMR spectrum reveals residual ethyl acetate, as the product was not fully evaporated under reduced pressure to avoid potential evaporation.

*(Product mass too low for UPLC-ESI analysis.)*

**^1^H-NMR** (600 MHz, CDCl_3_): δ [ppm] = 3.67 (m, 1H; CH_2_), 3.44 (m, 1H; CH_2_), 3.40 (m, 1H; CH), 1.34 (d, *J* = 6.9 Hz 3H; (CH_3_)).

**^13^C-NMR** (151 MHz, CDCl_3_): δ [ppm] = 69.34 (1C; CH_2_-OH), 38.24 (1C; [C](CH_3_)-SH), 20.8 (2C; (CH_3_)_2_).

### 1c – 2-mercapto-2-methylpropan-1-ol

A solution of ethyl 2-bromo-2-methylpropanoate **SI-1c** (2.00 g, 1.0 equiv., 10.3 mmol) and potassium thioacetate (1.52 g, 1.3 equiv., 13.3 mmol) in DMF (20 mL) were stirred at room temperature for 80 minutes. The mixture took on a yellow-orange color. The reaction was monitored for completeness by TLC (10 % ethyl acetate in hexane (*v/v*), R*_f, product_* = 0.44; R*_f, educt_* = 0.68). After the reaction was complete, water (20 mL) was added. The solution clarified and turned yellow. The reaction mixture was extracted with MTBE (3 x 20 mL); combined organic phases were dried over Na_2_SO_4_, filtrated, and the solvent was removed under reduced pressure. The remaining orange oil was purified by column chromatography (0 → 10 % ethyl acetate in hexane) to obtain a yellow oil of unpleasant odor 1.37 g (70 %, 7.2 mmol).

**SI-2c: UPLC-MS (ESI)** *m/z* calcd. for C_8_H_15_O_3_S^+:^ 191.07 [*M*+H]^+^; found: 191.14 [*M*+H]^+^.

A solution of ethyl 2-(acetylthio)-2-methylpropanoate **SI-2c** (1.37 g, 1.0 equiv., 7.20 mmol) in dry THF (10 mL) was added dropwise to the gray suspension of lithium aluminum hydride (546 mg, 2.0 equiv., 14.4 mmol) in dry THF (10 mL). The reaction mixture was stirred at 75 °C for 2 hours. The reaction was monitored for completeness by thin-layer chromatography (50 % ethyl acetate in hexane (*v/v*), stained with KMnO_4_, R*_f,product_* = 0.62). Ethyl acetate (10 mL) and hydrochloric acid (2 M, 5 mL) were added sequentially to the reaction mixture. Then, saturated sodium chloride solution was added until two phases formed. The organic phase was separated and subsequently extracted twice more with ethyl acetate (2 x 50 mL) from the aqueous phase. The combined organic phases were washed with saturated sodium chloride solution (30 mL), dried over sodium sulfate, filtered, and the solvent was removed under reduced pressure, with care taken to ensure that the volatile product **1c** was not removed. This yielded 407 mg (53 %, 3.84 mmol) of a pale-yellow oil with an unpleasant odor.

*(Product mass too low for UPLC-ESI analysis.)*

**^1^H-NMR** (600 MHz, CDCl_3_): δ [ppm] = 3.43 (s, 2H; CH_2_), 1.35 (s, 6H; (CH_3_)_2_).

**^13^C-NMR** (151 MHz, CDCl_3_): δ [ppm] = 73.7 (1C; CH_2_-OH), 47.1 (1C; [C](CH_3_)_2_-SH), 28.6 (2C; (CH_3_)_2_).

## General synthesis for the activation of thiols

**Mercaptoethanol derivative 1a-c** (1.0 equiv.) were dissolved in **DCM/MeOH** (80 mL, 95/5 (v/v)). With stirring, **1,2-bis(5-nitropyridin-2-yl) disulfide** (1.5 equiv.) was added, causing the solution to turn yellow. The reaction mixture was stirred at room temperature for 24 hours. The reaction was monitored for completeness by **thin-layer chromatography** (20 % ethyl acetate in DCM (*v/v*)). The solvent was removed under reduced pressure, and the resulting orange residue was applied to silica gel and purified by **column chromatography** (0 → 30% EtOAc in DCM (*v/v*)). The solvent was then removed under reduced pressure, and the product was lyophilized for 2 hours for the subsequent reaction.

### SI-3a – 2-((5-nitropyridin-2-yl)disulfaneyl)ethan-1-ol

Synthesis was performed following the general synthesis for the activation of thiols using **1a** (551 mg, 1.0 equiv., 7.05 mmol) in 133 mL DCM/MeOH, adding **1,2-bis(5-nitropyridin-2-yl) disulfide (3.283 g, 1.5 equiv., 10.58 mmol). We were not able to separate the product SI-3a from 5-Nitropyridin-2-thiol using silica chromatography. Dissolving the product mixture in DCM/ethyl acetate (8:2) and filtering the resulting suspension yielded an orange powder, with over >90 % pure after lyophilization (695 mg, 42 %).**

**UPLC-MS (ESI)** *m/z* calcd. for C_7_H_9_N_2_O_3_S_2_^+^: 233.00 [*M*+H]^+^; found: 233.09 [*M*+H]^+^.

**^1^H-NMR** (600 MHz, CDCl_3_): δ [ppm] = 9.23 (d, *J* = 2.6 Hz, 1H; Ar-H), 8.57 (dd, *J* = 8.9, 2.7 Hz, 1H; Ar-H), 8.09 (d, *J* = 8.9 Hz, 1H; Ar-H), 3.63 (t, *J* = 6.2 Hz, 3H; C[H_2_]-OH), 2.98 (t, *J* = 6.2 Hz, 3H; C[H_2_]-S-SR).

**^13^C-NMR** (151 MHz, CDCl_3_): δ [ppm] = 167.8 (1C; Ar-H), 144.8 (1C; Ar-H), 142.2 (1C; Ar-H), 132.6 (1C; Ar-H), 119.5 (1C; Ar-H), 59.0 (1C; CH_2_-OH), 41.3 (1C; CH_2_-SS).

### SI-3b – 2-((5-nitropyridin-2-yl)disulfaneyl)propan-1-ol

Synthesis was performed following the general synthesis for the activation of thiols using **1b** (400 mg, 1.0 equiv., 4.34 mmol) in 50 mL DCM/MeOH, adding **1,2-bis(5-nitropyridin-2-yl) disulfide (2.02 g, 1.5 equiv., 6.51 mmol). After silica column purification and lyophilization, the product was obtained as yellow powder (612 mg, 4.34 mmol, 57 %).**

**UPLC-MS (ESI)** *m/z* calcd. for C_8_H_11_N_2_O_3_S_2_^+^: 247.02 [*M*+H]^+^; found: 247.04 [*M*+H]^+^.

**^1^H-NMR** (600 MHz, CDCl_3_): δ [ppm] = 9.31 (d, *J* = 2.5 Hz, 1H; Ar-H), 8.35 (dd, *J* = 8.8, 2.6 Hz, 1H; Ar-H), 7.65 (d, *J* = 8.8 Hz, 1H; Ar-H), 3.67* (dd, *J* = 12.3, 4.0 Hz, 2H; C[H_2_]-OH), 3.39* (dd, *J* = 12.3, 7.5 Hz, 2H; C[H_2_]-OH), 3.16 (pd, *J* = 7.0, 4.0 Hz, 1H; C[H]-S-SR), 1.34* (s, 3H; CH_3_), 1.33* (s, 3H; CH_3_).

*signals corresponding the different enantiomers, respectively

**^13^C-NMR** (151 MHz, CDCl_3_): δ [ppm] = 167.8 (1C; Ar-H), 145.5 (1C; Ar-H), 131.5 (1C; Ar-H), 121.2 (1C; Ar-H), 64.1 (1C; CH_2_-OH), 50.0 (1C; CH_2_-SS), 16.9 (1C; CH_3_).

### SI-3c – 2-methyl-2-((5-nitropyridin-2-yl)disulfaneyl)propan-1-ol

Synthesis was performed following the general synthesis for the activation of thiols using **1c** (445 mg, 1.0 equiv., 4.19 mmol) in 80 mL DCM/MeOH, adding **1,2-bis(5-nitropyridin-2-yl) disulfide (21.955 g, 1.5 equiv., 6.3 mmol). After silica column purification, the product SI-3c crystallized at room temperature as colorless crystals from a yellow oil (528 mg, 2.01 mmol, 48%).**

**UPLC-MS (ESI)** *m/z* calcd. for C_9_H_13_N_2_O_3_S_2_^+^: 261.04 [*M*+H]^+^; found: 261.11 [*M*+H]^+^.

**^1^H-NMR** (600 MHz, CDCl_3_): δ [ppm] = 9.31 (d, *J* = 2.5 Hz, 1H; Ar-H), 8.37 (dd, *J* = 8.8, 2.6 Hz, 1H; Ar-H), 7.63 (d, *J* = 8.8 Hz, 1H; Ar-H), 4.14 (s, 1H; CH_2_-O[H]), 3.33 (s, 2H; CH_2_), 1.38 (s, 6H; (CH_3_)_2_).

**^13^C-NMR** (151 MHz, CDCl_3_): δ [ppm] = 167.8 (1C; Ar-H), 145.4 (1C; Ar-H), 142.8 (1C; Ar-H), 131.5 (1C; Ar-H), 121.3 (1C; Ar-H), 68.0 (1C; CH_2_), 55.1 (1C; [C](CH_3_)_2_), 24.3 (2C; (CH_3_)_2_).

## General synthesis of the activated carbonates

The activated disulfide (1.0 equiv.) was prepared in a round-bottom flask and dissolved in dry **MeCN** (5 mL) under a nitrogen atmosphere. **Triethylamine** (1.2 equiv.) was added. The reaction mixture was cooled in a sodium chloride ice bath. **4-nitrophenyl chloroformate** (1.2 equiv.) was dissolved in dry MeCN (5 mL) and added to the reaction mixture, while stirring and cooling. The solution cleared from orange and turned pale yellow.

The mixture was stirred for 30 minutes in the sodium chloride ice bath, then stirred for an additional 24 hours at room temperature. The reaction completion was monitored by **thin-layer chromatography** (30 % ethyl acetate in hexane). Upon completion, the mixture was diluted with **ethyl acetate** (20 mL) and washed sequentially with **dilute hydrochloric acid** (2 M, 2x30 mL) and saturated **sodium chloride solution** (30 mL). The organic phase was dried over sodium sulfate, filtered, and the solvent was removed under reduced pressure. The resulting residue was dissolved in 70 % MeCN, 28 % water, 1.25 % DMSO purified by **preparative HPLC** and lyophilized overnight.

### 2a – 4-nitrophenyl (2-((5-nitropyridin-2-yl)disulfaneyl)ethyl) carbonate

Compound **2a** was synthesized following the general procedure for synthesis of activated carbonates using **SI-3a** (195 mg, 1.0 equiv., 0.84 mmol), triethylamine (140 µL, 1.2 equiv., 1.01 mmol) and **4-nitrophenyl chloroformate (205 mg, 1.2 equiv., 1.02 mmol). Preparative HPLC was used to purify the reaction mixture and yield 118 mg of 2a (297 µmol, 35 %) as colorless powder.**

**UPLC-MS (ESI)** *m/z* calcd. for C_14_H_12_N_3_O_7_S_2_^+^: 398.01 [*M*+H]^+^; found: 398.18 [*M*+H]^+^.

**^1^H-NMR** (600 MHz, CDCl_3_): δ [ppm] = 9.30 (d, *J* = 2.6 Hz, 1H; Ar-H), 8.42 (dd, *J* = 8.8, 2.6 Hz, 1H; Ar-H), 8.29 (m, 2H; Ar-H), 7.86 (dd, *J* = 8.9, 0.7 Hz, 1H; Ar-H), 7.38 (m, 2H; Ar-H), 4.56 (t, J = 6.3 Hz, 2H; CH_2_), 3.21 (t, *J* = 6.3 Hz, 2H; CH_2_).

**^13^C-NMR** (151 MHz, CDCl_3_): δ [ppm] = 167.5 (1C; Ar-H), 155.4 (1C; Ar-H), 152.4 (1C; CO­_3_), 145.7 (1C; Ar-H), 145.5 (1C; Ar-H), 142.5 (1C; Ar-H), 131.8 (1C; Ar-H), 125.5 (2C; Ar-H), 121.8 (2C; Ar-H), 119.8 (1C; Ar-H), 66.5 (1C; CH_2_), 37.1 (1C; CH_2_).

### 2b – 4-nitrophenyl (2-((5-nitropyridin-2-yl)disulfaneyl)propyl) carbonate

Compound **2b** was synthesized following the general procedure for synthesis of activated carbonates using **SI-3b** (201 mg, 1.0 equiv., 0.84 mmol), triethylamine (136 µL, 1.2 equiv., 0.98 mmol) and **4-nitrophenyl chloroformate (198 mg, 1.2 equiv., 0.98 mmol). Preparative HPLC was used to purify the reaction mixture and yield 133 mg of 2b (297 µmol, 40 %) as colorless powder.**

**UPLC-MS (ESI)** *m/z* calcd. for C_15_H_14_N_3_O_7_S_2_^+^: 412.03 [*M*+H]^+^; found: 412.17 [*M*+H]^+^.

**^1^H-NMR** (600 MHz, CDCl_3_): δ [ppm] = 9.27 (d, *J* = 2.6 Hz, 1H; Ar-H), 8.40 (dd, *J* = 8.8, 2.6 Hz, 1H; Ar-H), 8.28 (m, 2H; Ar-H), 7.90 (dd, *J* = 8.9, 0.7 Hz, 1H; Ar-H), 7.37 (m, 2H; Ar-H), 4.43 (dd, *J* = 11.3, 6.1 Hz, 1H; CH_2_), 4.35 (dd, *J* = 11.3, 6.4 Hz, 1H; CH_2_), 3.40 (s, *J* = 6.9 Hz, 1H; CH), 1.45 (d, *J* = 7.0 Hz, 4H; CH_3_).

**^13^C-NMR** (151 MHz, CDCl_3_): δ [ppm] = 168.2 (1C; Ar-H), 155.4 (1C; Ar-H), 152.3 (1C; CO_3_), 145.7 (1C; Ar-H), 145.3 (1C; Ar-H), 142.4 (1C; Ar-H), 131.8 (1C; Ar-H), 125.5 (1C; Ar-H), 121.8 (2C; Ar-H), 119.8 (2C; Ar-H), 71.0 (1C; CH_2_), 44.9 (1C; CH(CH_3_), 17.1 (1C; CH_3_).

### 2c – 2-methyl-2-((5-nitropyridin-2-yl)disulfaneyl)propyl (4-nitrophenyl) carbonate

Compound **2c** was synthesized following the general procedure for synthesis of activated carbonates using **SI-3c** (201 mg, 1.0 equiv., 0.77 mmol), triethylamine (129 µL, 1.2 equiv., 0.93 mmol) and **4-nitrophenyl chloroformate (190 mg, 1.2 equiv., 0.94 mmol). Preparative HPLC was used to purify the reaction mixture and yield 80 mg of 2c (190 µmol, 24 %) as partially solid and high viscos oil.**

**UPLC-MS (ESI)** *m/z* calcd. for C_16_H_16_N_3_O_7_S_2_^+^: 426.04 [*M*+H]^+^; found: 426.26 [*M*+H]^+^.

**^1^H-NMR** (600 MHz, CDCl_3_): δ [ppm] = 9.26 (d, *J* = 2.7 Hz, 1H; Ar-H), 8.38 (dd, *J* = 8.8, 2.6 Hz, 1H; Ar-H), 8.28 (m, 2H; Ar-H), 7.94 (d, J = 9.0 Hz, 1H; Ar-H), 7.37 (m, 2H; Ar-H), 4.24 (s, 2H, CH_2_), 1.45 (s, 6H, (CH_3_)_2_).

**^13^C-NMR** (151 MHz, CDCl_3_): δ [ppm] = 168.6 (1C; Ar-H), 155.4 (1C; Ar-H), 152.3 (1C; CO3), 145.7 (1C; Ar-H), 145.1 (1C; Ar-H), 142.4 (1C; Ar-H), 131.7 (1C; Ar-H), 125.5 (2C; Ar-H), 121.8 (2C; Ar-H), 119.9 (1C; Ar-H), 74.3 (1C, CH_2_), 51.0 (1C, [C](CH_3_)_2_), 24.5 (2C, (CH_3_)_2_).

### 5 – 2-methyl-1-(((4-nitrophenoxy)carbonyl)oxy)propan-2-yl carbamo(dithioperoxo)imidate

**2c** (30 mg, 71 **µmol**) was incubated with thiourea (54 mg, 10 equiv., 710 **µmol) and formamidine disulfide (107 mg, 10 equiv., 710 µmol) in 2 mL DMF at room temperature for 1 h. The resulting reaction mixture was purified using preparative HPLC yielding a white powder (10.4 mg, 30 µmol, 43 %).**

**UPLC-MS (ESI)** *m/z* calcd. for C_12_H_16_N_3_O_5_S_2_^+^: 346.1 [*M*+H]^+^; found: 346.1 [*M*+H]^+^.

**HR-MS (ESI)** *m/z* calcd. for C_12_H_16_N_3_O_5_S_2_^+^: 345.0526 [*M*+H]^+^; found: 346.0522 [*M*+H]^+^.

**^1^H-NMR** (600 MHz, DMSO-*d*_6_) δ [ppm] = 9.88 (s, 2H; NH_2_), 9.32 (s, 2H, NH_2_), 8.37 – 8.31 (m, 2H; Ar-H), 7.61 – 7.56 (m, 2H, Ar-H), 4.29 (s, 2H, CH_2_), 1.41 (s, 6H; CH_3_).

**^13^C-NMR** (151 MHz, CDCl_3_): δ [ppm] = 169.0 (1C; C(NH_2_)(NH­_2_)), 155.0 (1C; Ar-H), 151.8 (1C; CO_3_), 145.3 (1C; Ar-H), 125.4 (1C; Ar-H), 122.6 (1C; Ar-H), 73.5 (1C; CH_2_), 51.8 (1C; [C](CH_3_)_2_), 23.16 (1C; (CH_3_)_2_).

# Supplementary References

[1] A. F. L. Schneider, M. Kithil, M. C. Cardoso, M. Lehmann, C. P. R. Hackenberger, *Nat Chem* **2021**, *13*, 530-539.

[2] J. V. V. Arafiles, J. Franke, L. Franz, J. Gomez-Gonzalez, K. Kemnitz-Hassanin, C. P. R. Hackenberger, *J Am Chem Soc* **2023**, *145*, 24535-24548.

[3] a) J. D. Sadowsky, T. H. Pillow, J. Chen, F. Fan, C. He, Y. Wang, G. Yan, H. Yao, Z. Xu, S. Martin, D. Zhang, P. Chu, J. Dela Cruz-Chuh, A. O'Donohue, G. Li, G. Del Rosario, J. He, L. Liu, C. Ng, D. Su, G. D. Lewis Phillips, K. R. Kozak, S. F. Yu, K. Xu, D. Leipold, J. Wai, *Bioconjug Chem* **2017**, *28*, 2086-2098; b) T. H. Pillow, J. D. Sadowsky, D. Zhang, S. F. Yu, G. Del Rosario, K. Xu, J. He, S. Bhakta, R. Ohri, K. R. Kozak, E. Ha, J. R. Junutula, J. A. Flygare, *Chem Sci* **2017**, *8*, 366-370.

[4] a) D. L. Zhang, T. H. Pillow, Y. Ma, J. dela Cruz-Chuh, K. R. Kozak, J. D. Sadowsky, G. D. L. Philips, J. Guo, M. Darwish, P. Fan, J. T. Chen, C. R. He, T. Wang, H. Yao, Z. J. Xu, J. H. Chen, J. Wai, Z. H. Pei, C. E. C. A. Hop, S. C. Khojasteh, P. S. Dragovich, *Acs Med Chem Lett* **2016**, *7*, 988-993; b) M. Sengee, J. J. Eksteen, S. L. Nergard, T. Vasskog, L. K. Sydnes, *Bioconjug Chem* **2019**, *30*, 1489-1499; c) A. K. Jain, M. G. Gund, D. C. Desai, N. Borhade, S. P. Senthilkumar, M. Dhiman, N. K. Mangu, S. V. Mali, N. P. Dubash, S. Halder, A. Satyam, *Bioorg Chem* **2013**, *49*, 40-48; d) S. A. Kularatne, C. Venkatesh, H.-K. R. Santhapuram, K. Wang, B. Vaitilingam, W. A. Henne, P. S. Low, *Journal of Medicinal Chemistry* **2010**, *53*, 7767-7777.

[5] a) R. M. Beesley, C. K. Ingold, J. F. Thorpe, *J. Chem. Soc., Trans.* **1915**, *107*, 1080-1106; b) M. E. Jung, G. Piizzi, *Chem Rev* **2005**, *105*, 1735-1766.

[6] S. Tamara, M. A. den Boer, A. J. R. Heck, *Chemical Reviews* **2022**, *122*, 7269-7326.

[7] S. Sarabipour, C. King, K. Hristova, *Anal Biochem* **2014**, *449*, 155-157.

[8] J. Schindelin, I. Arganda-Carreras, E. Frise, V. Kaynig, M. Longair, T. Pietzsch, S. Preibisch, C. Rueden, S. Saalfeld, B. Schmid, J.-Y. Tinevez, D. J. White, V. Hartenstein, K. Eliceiri, P. Tomancak, A. Cardona, *Nature Methods* **2012**, *9*, 676-682.

# NMR Spectra

#
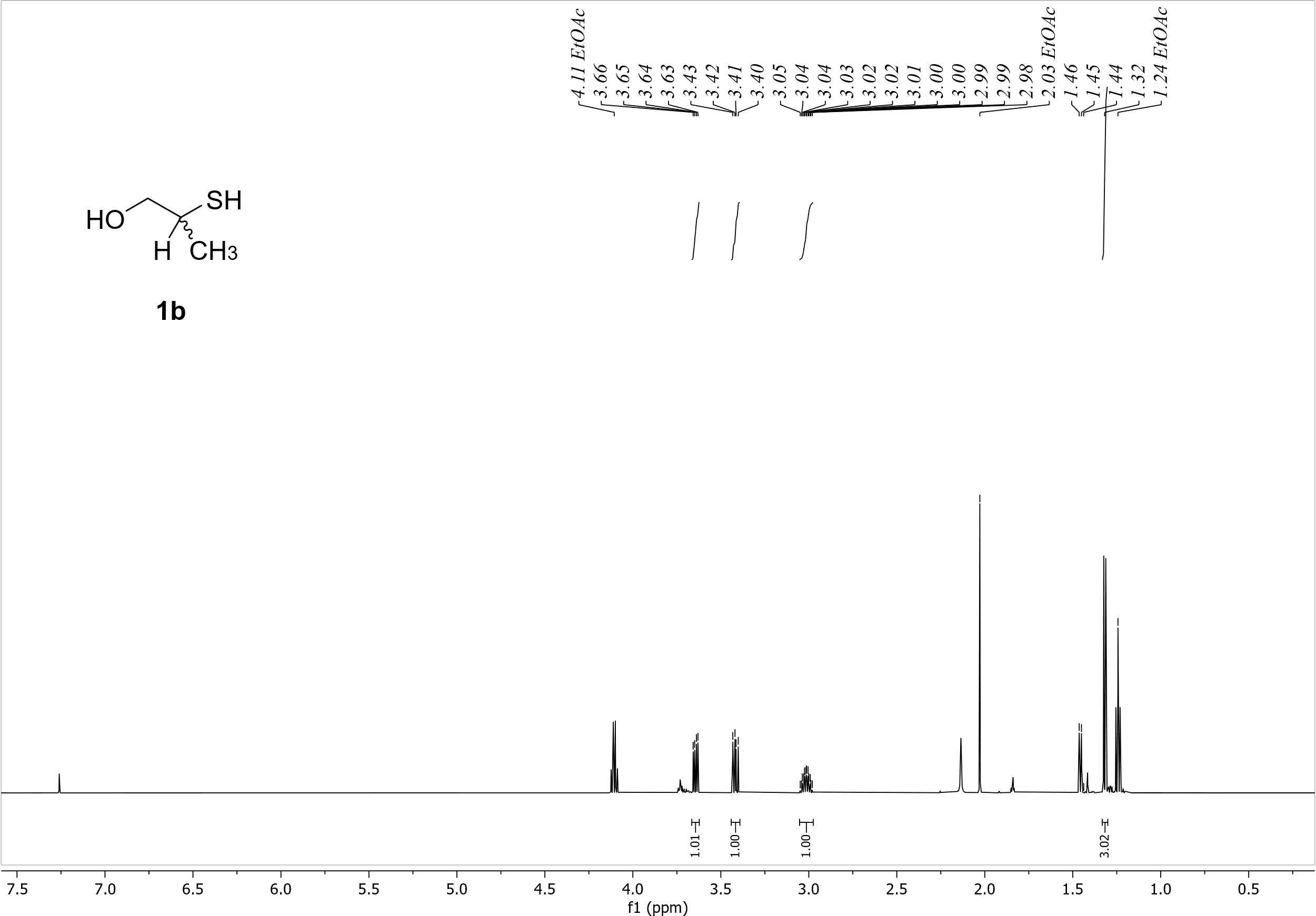

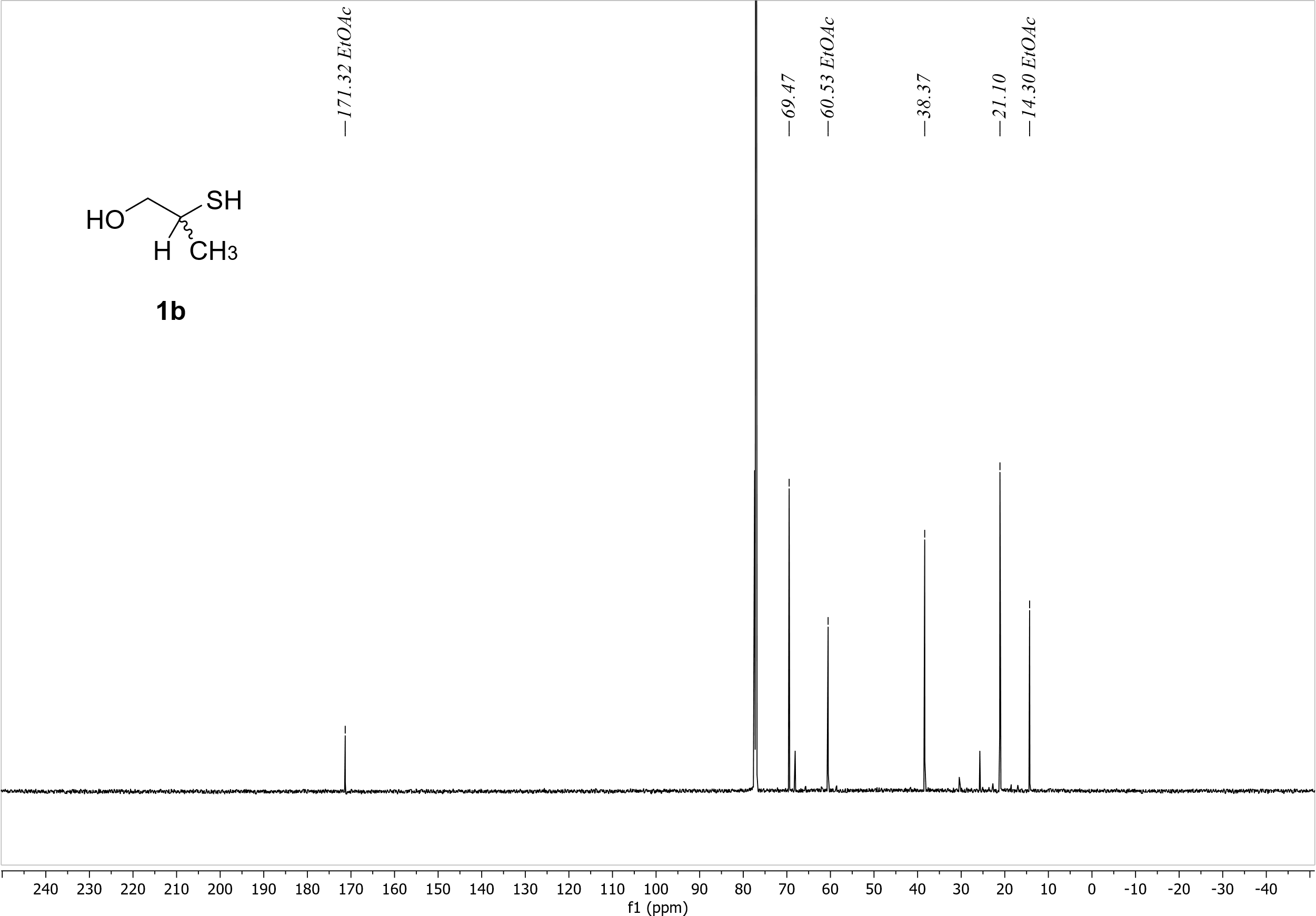

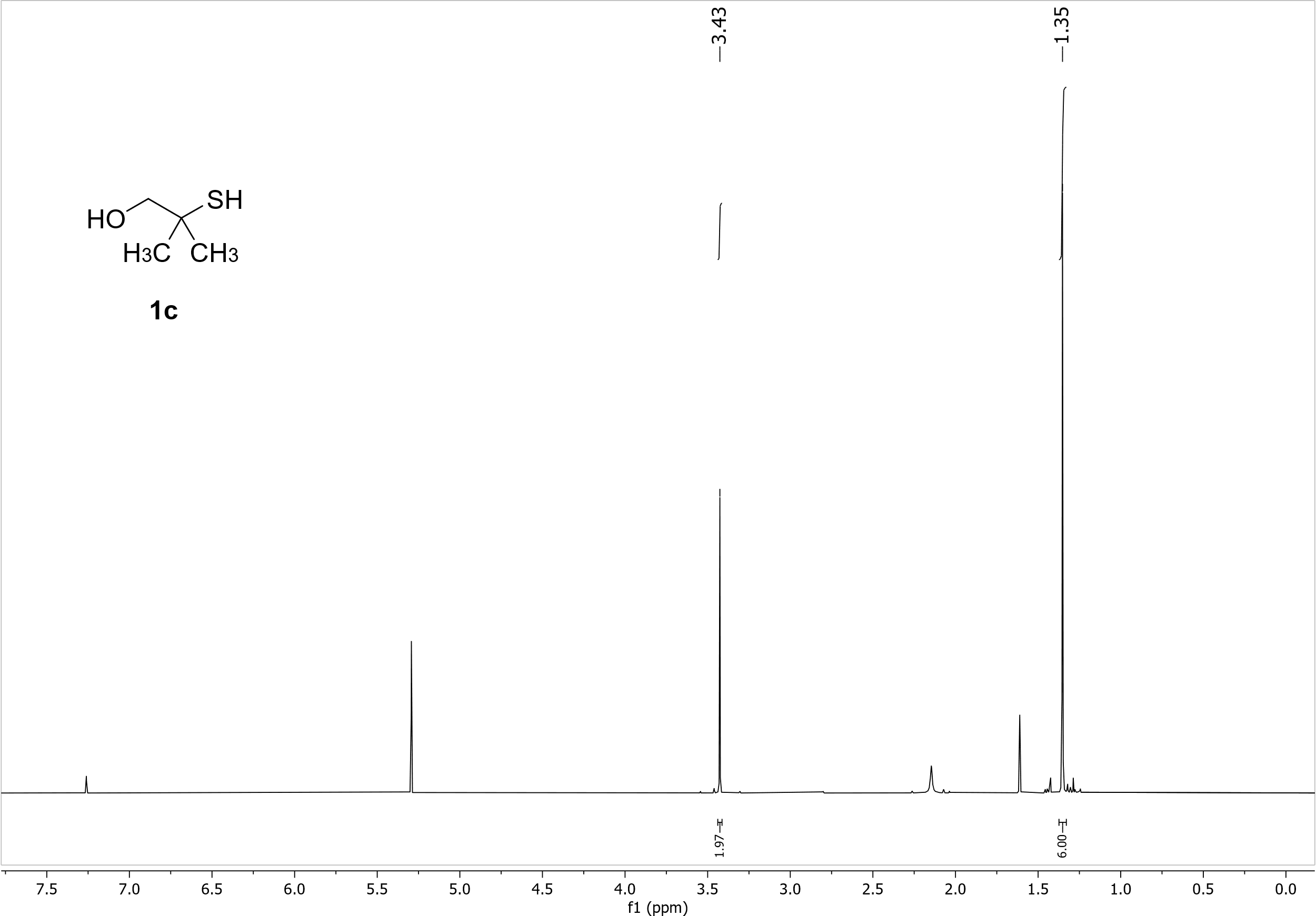

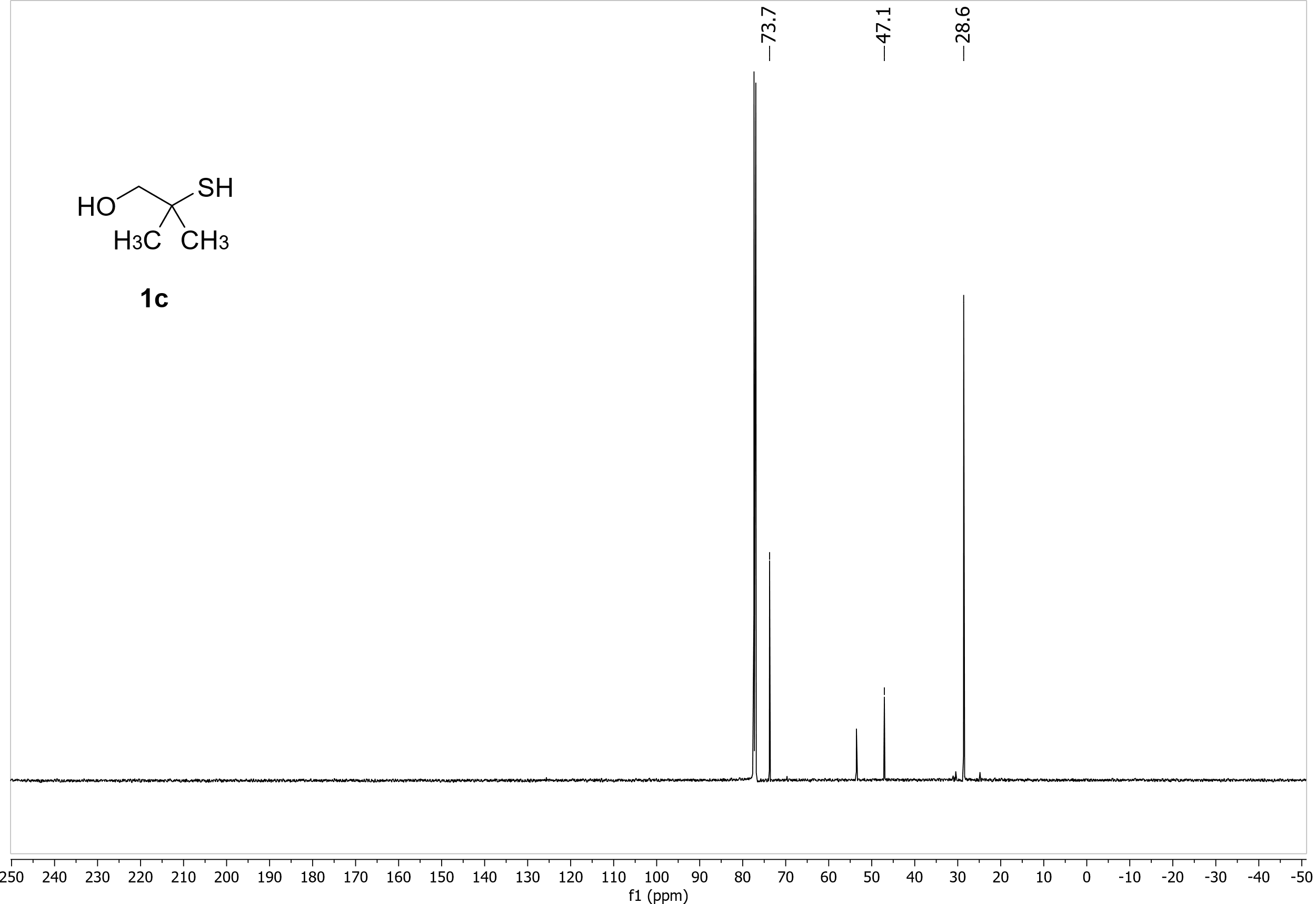

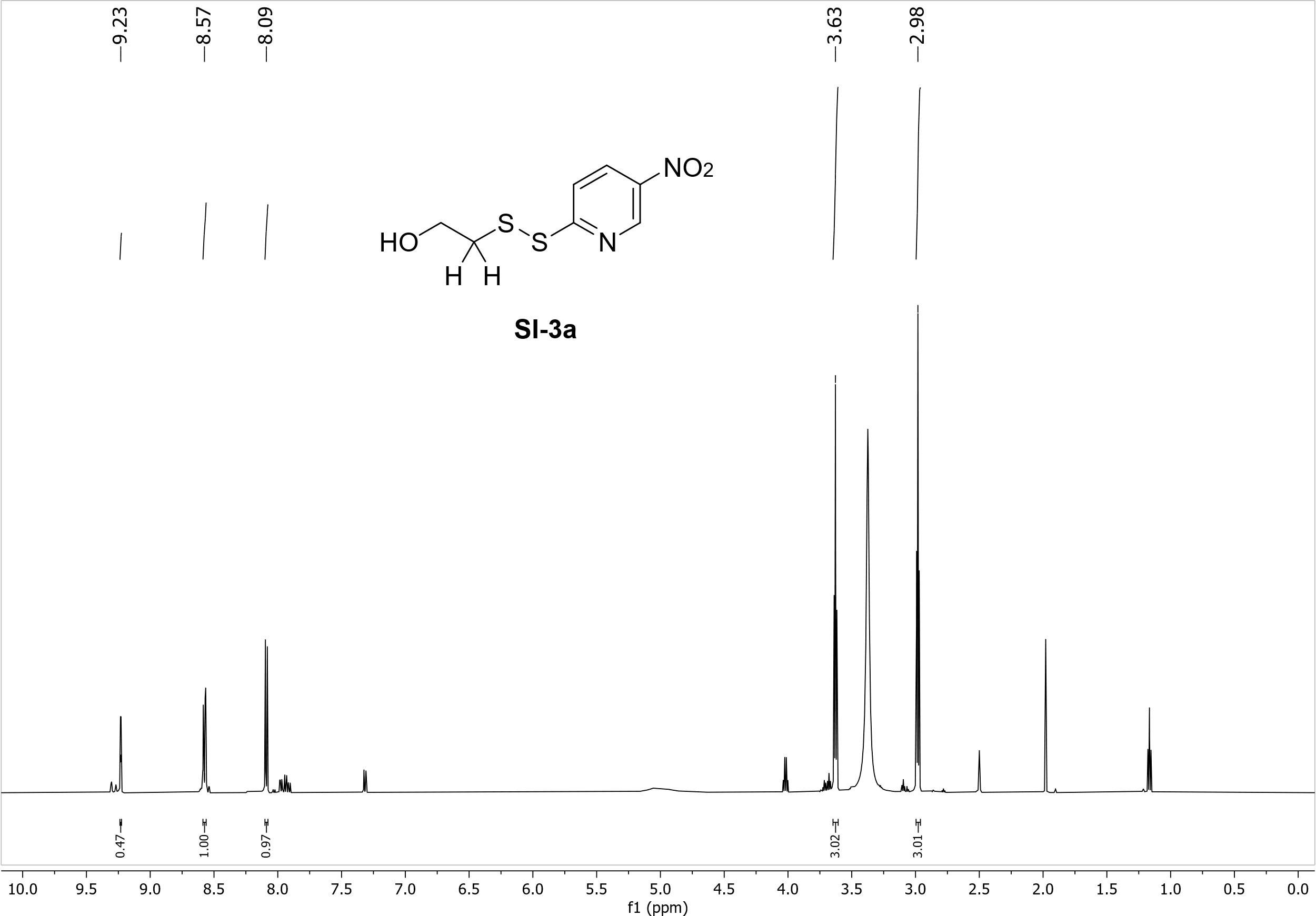

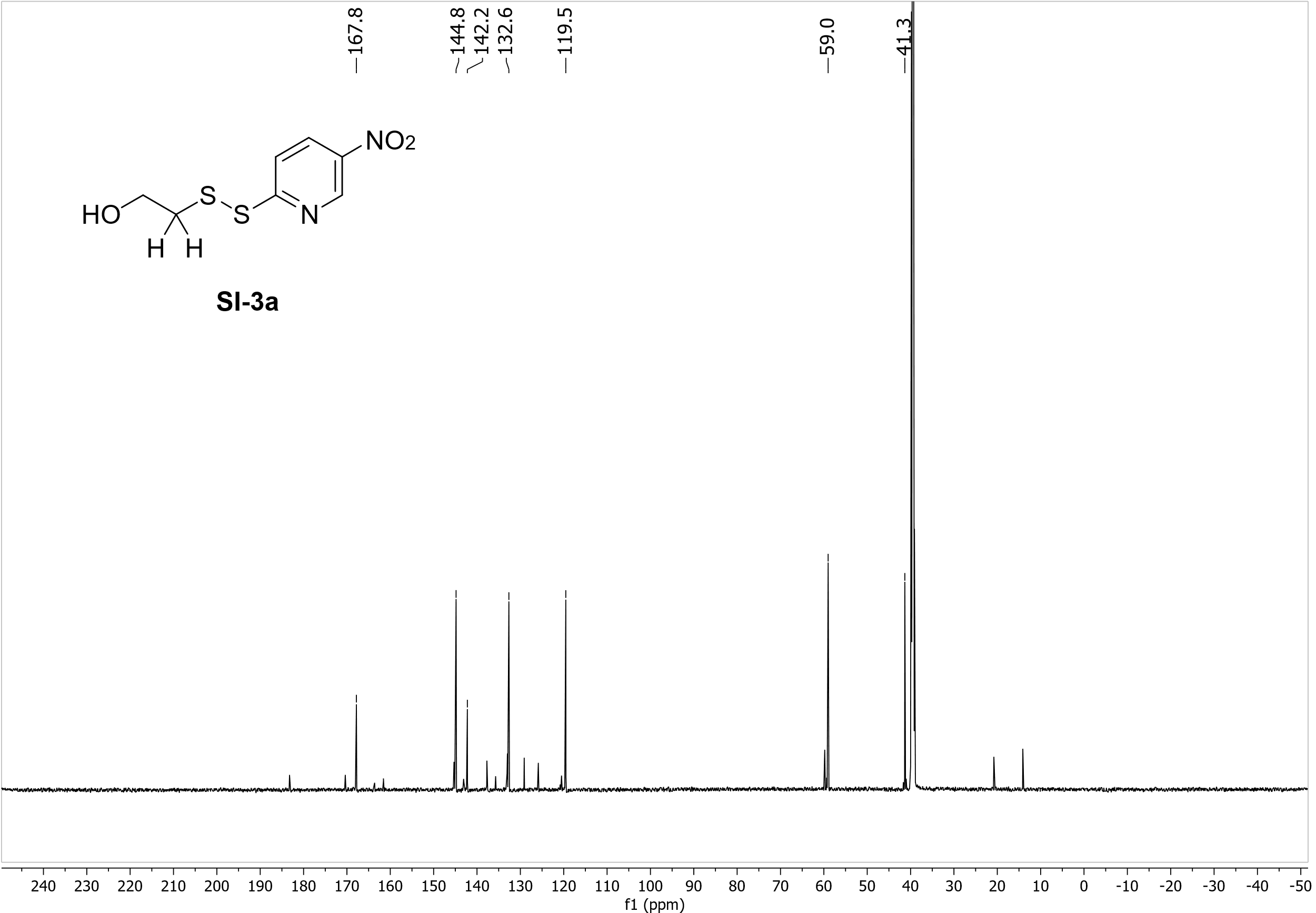

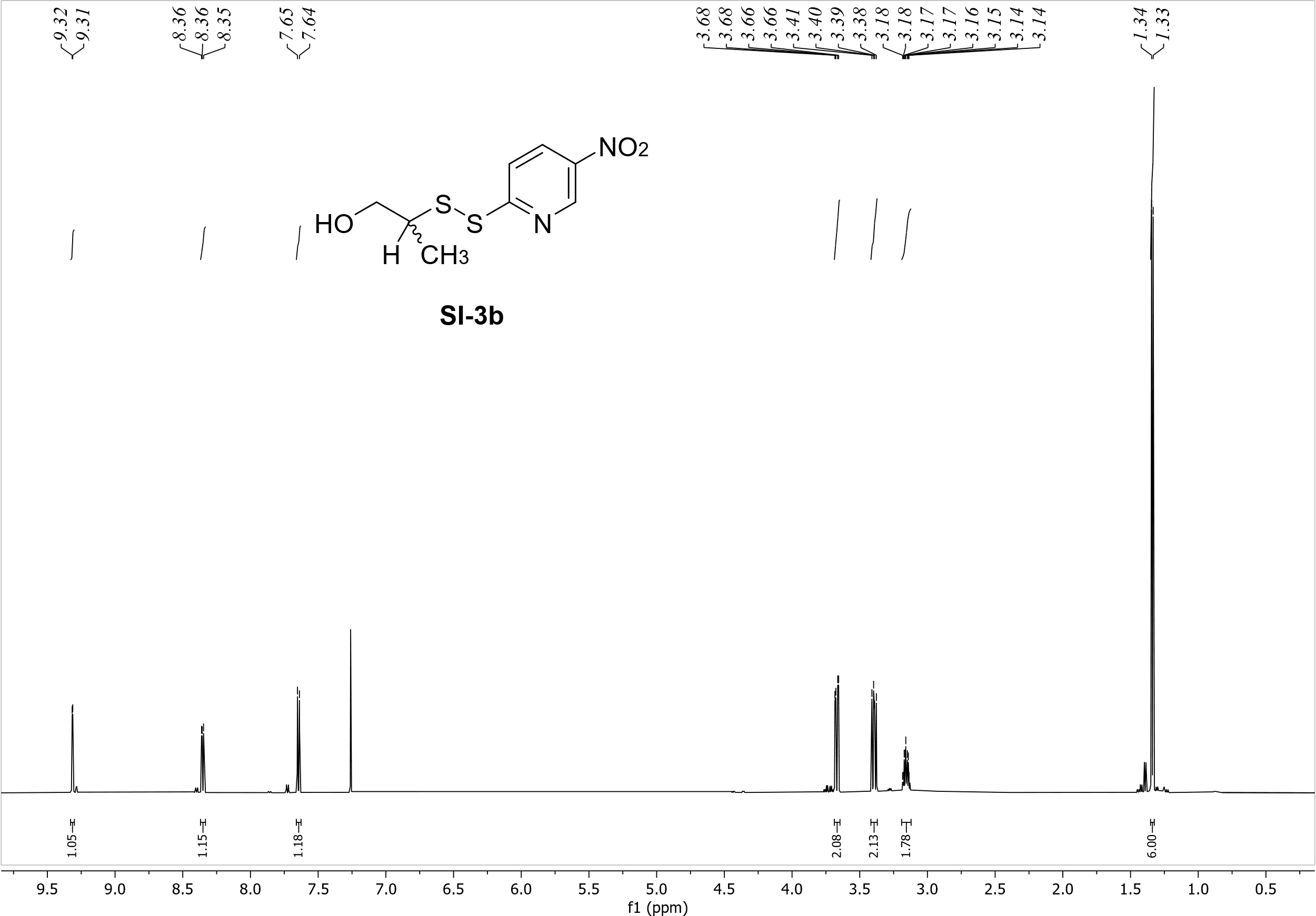

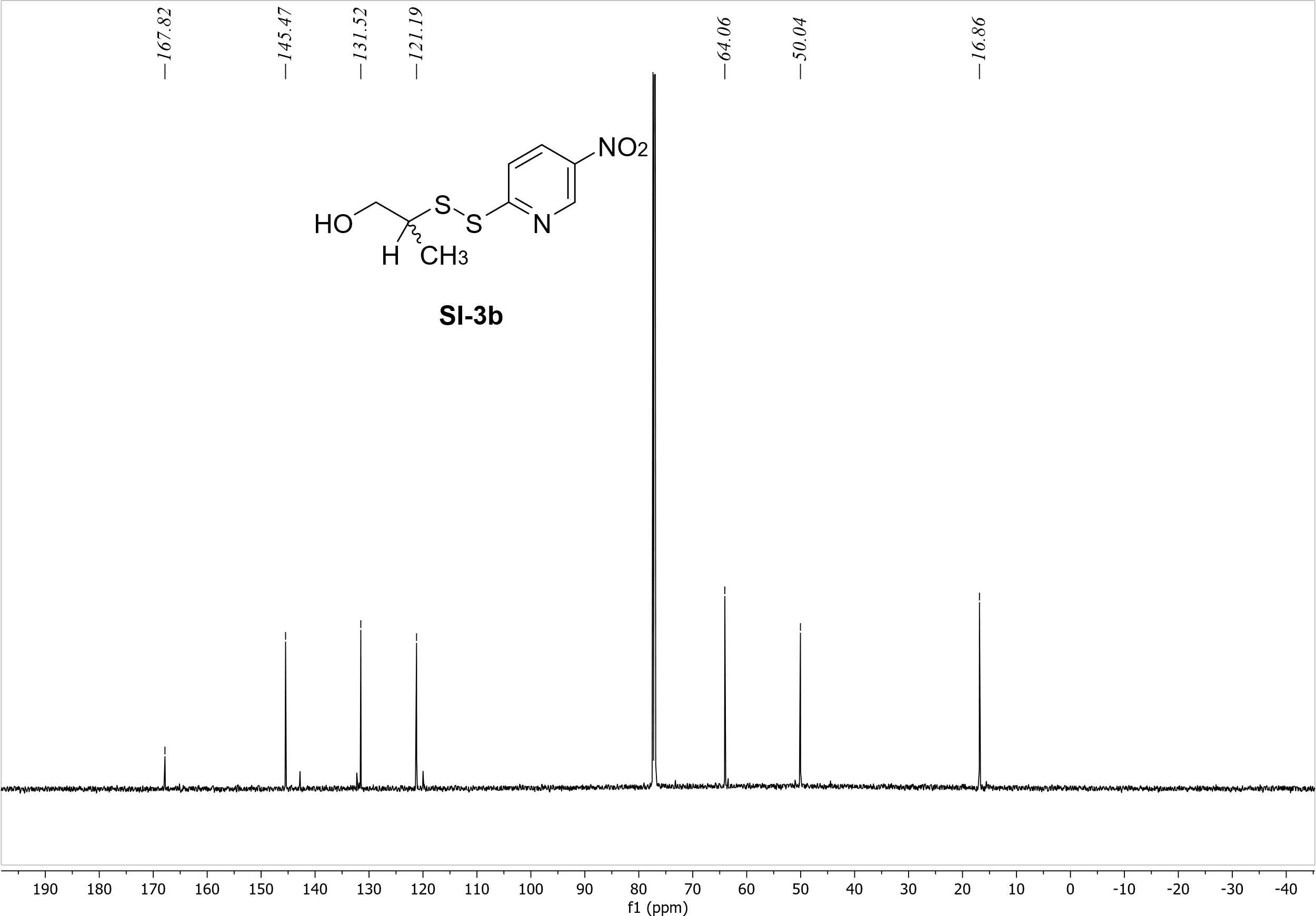

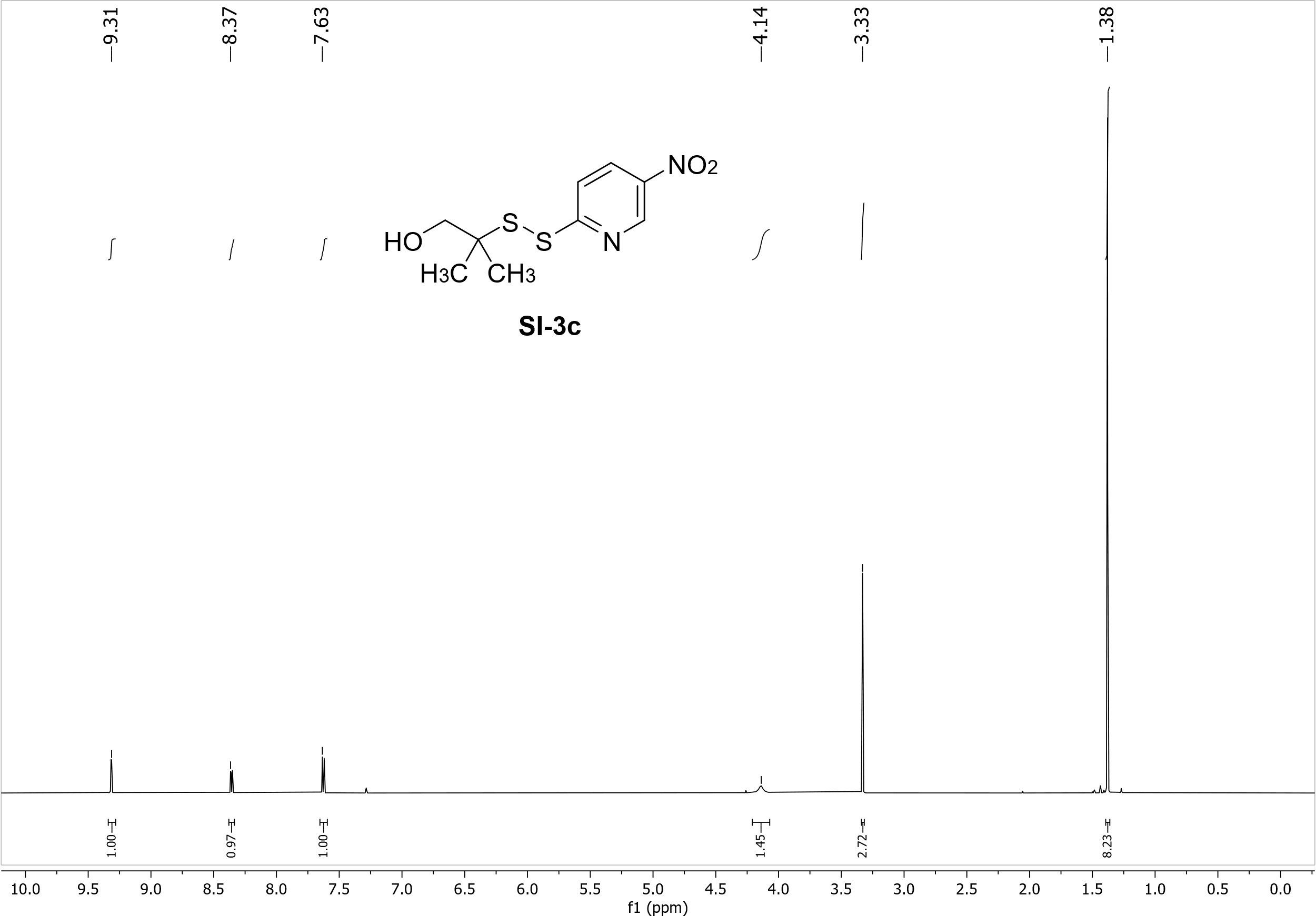

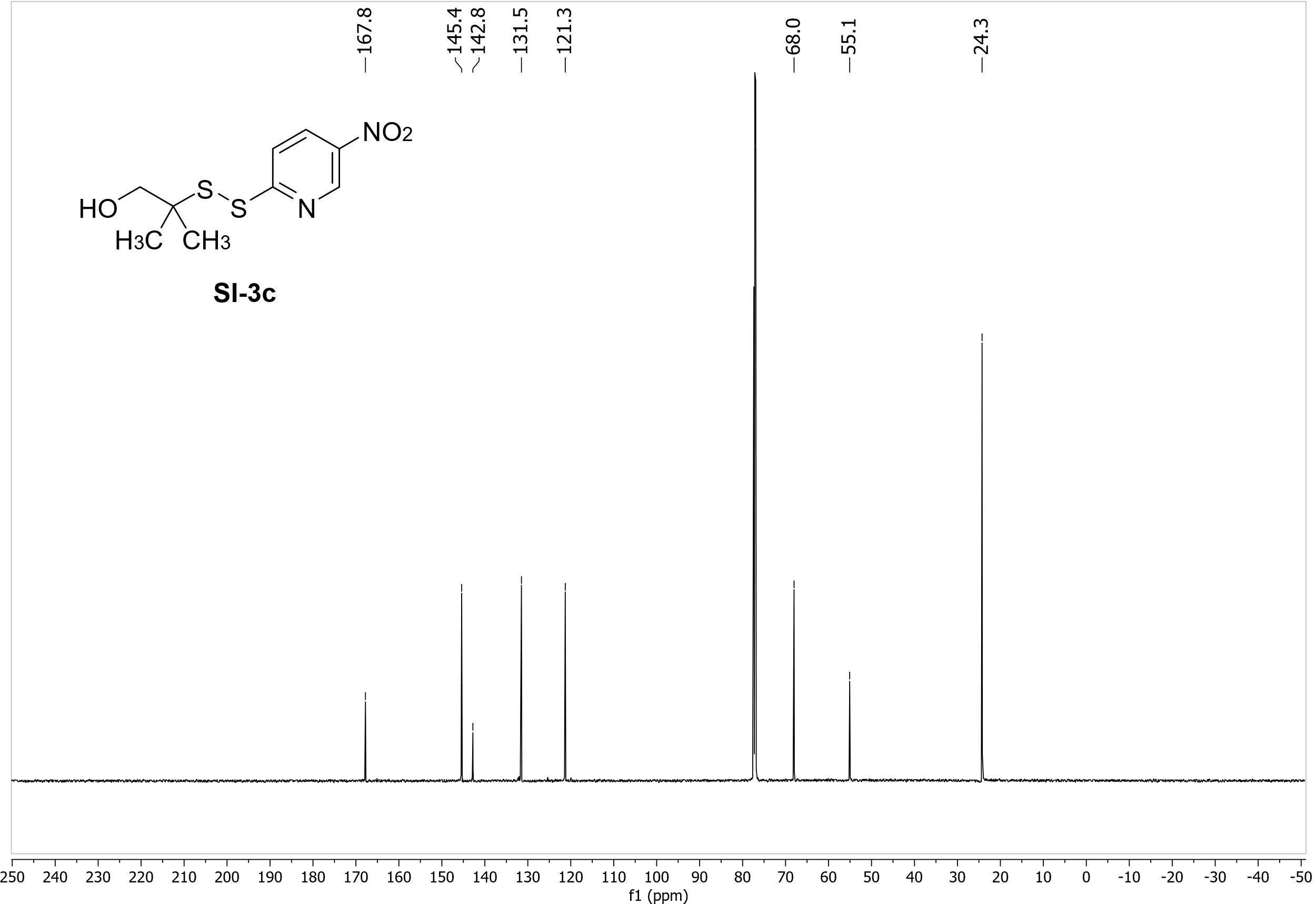

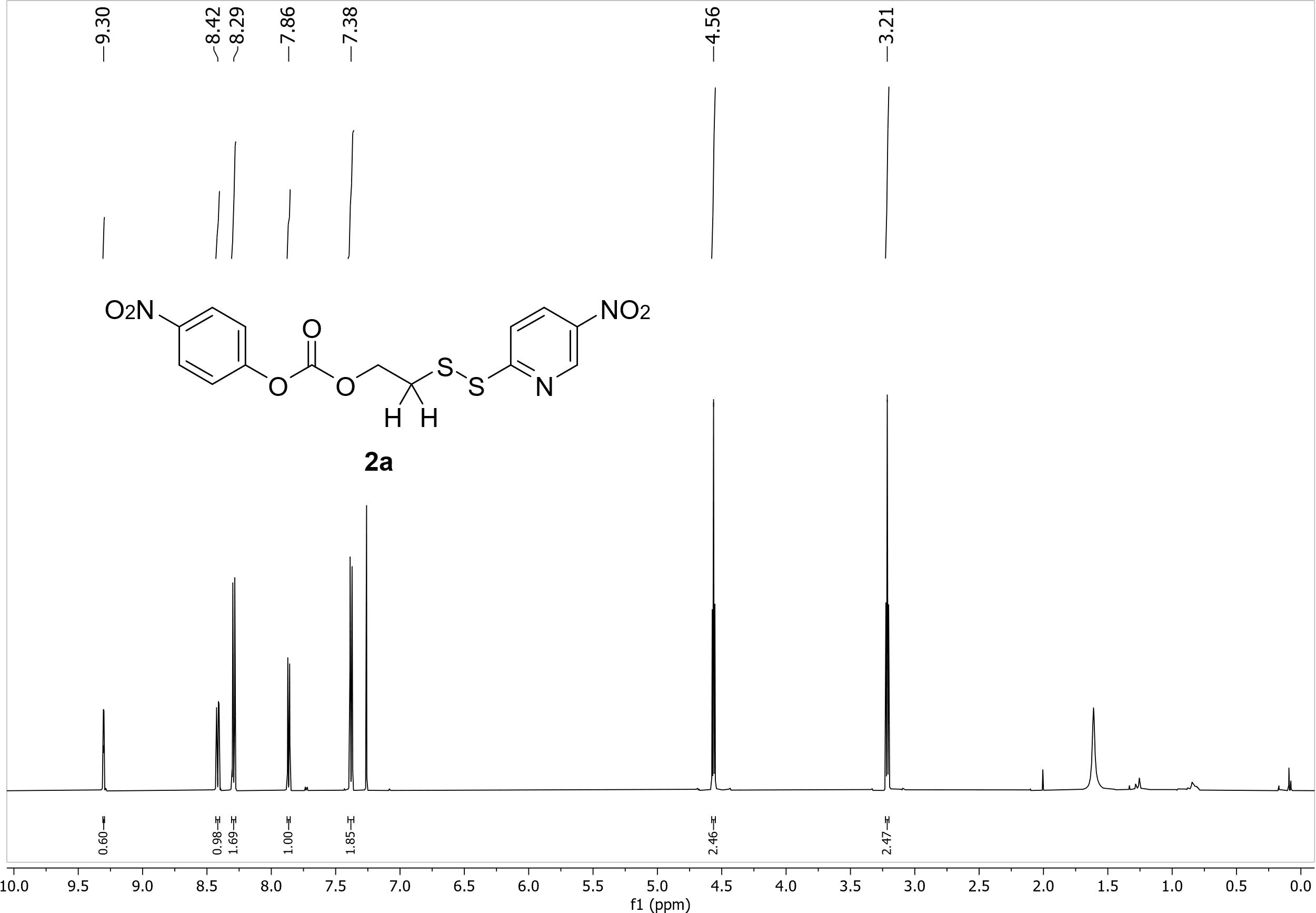

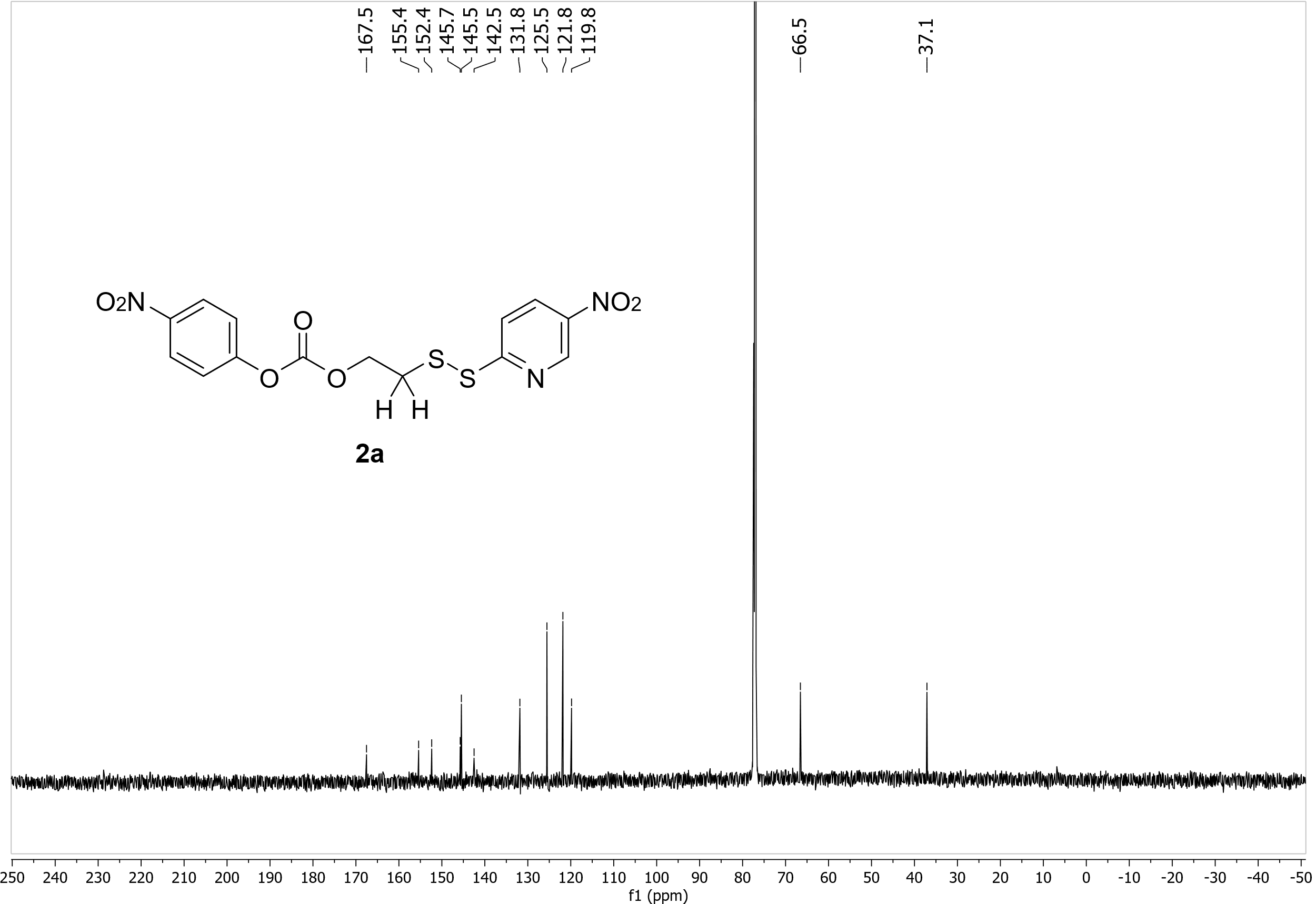

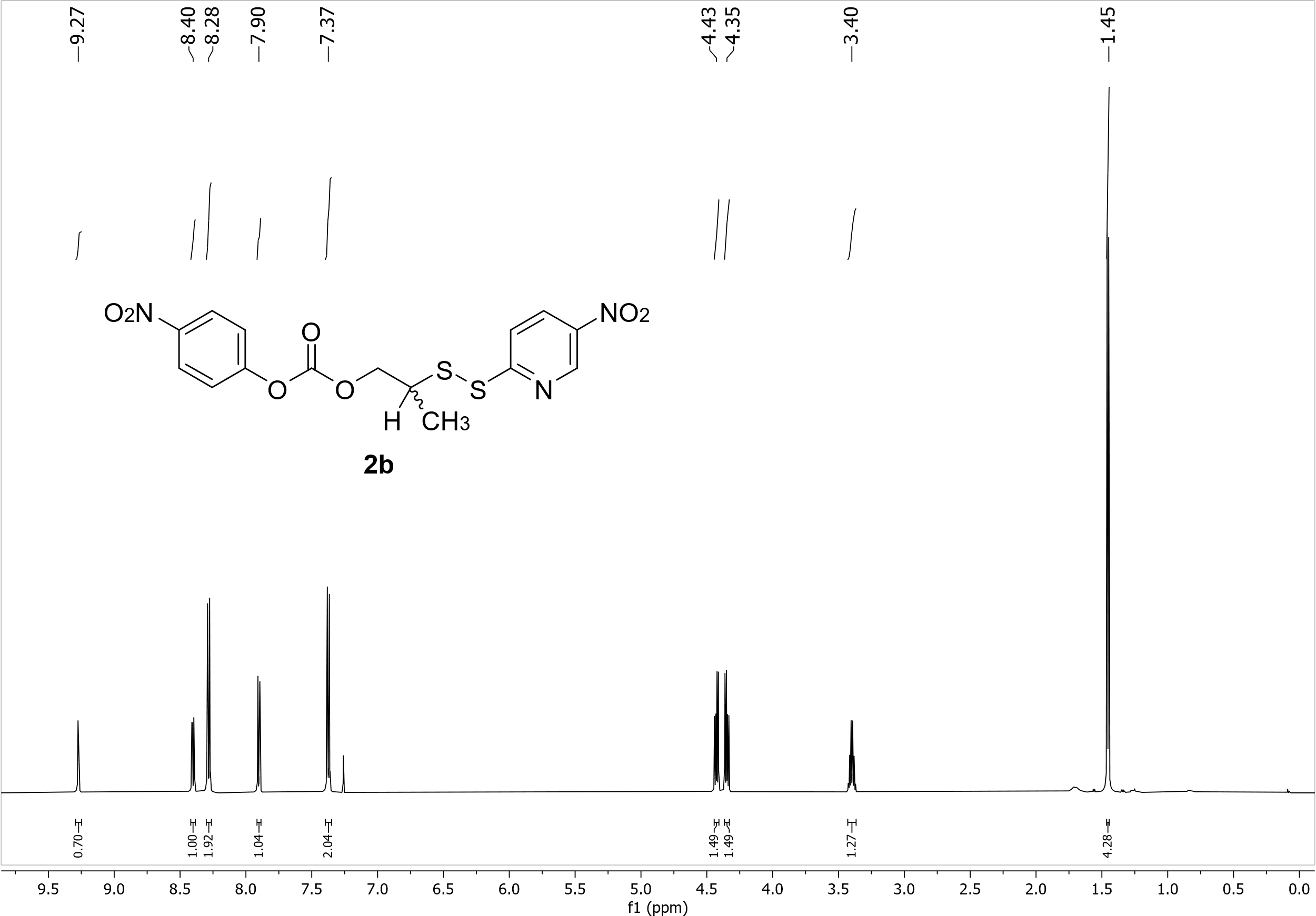

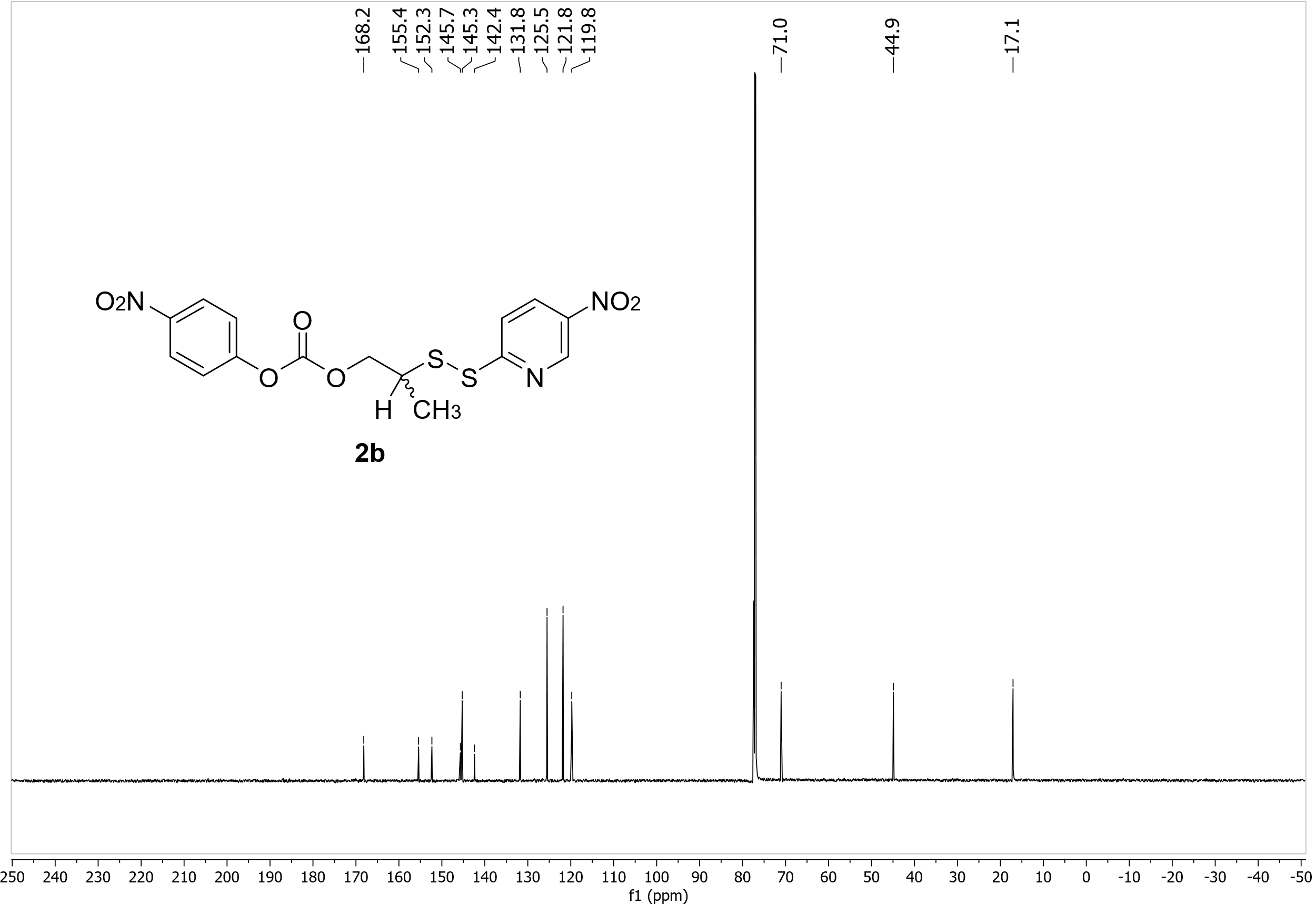

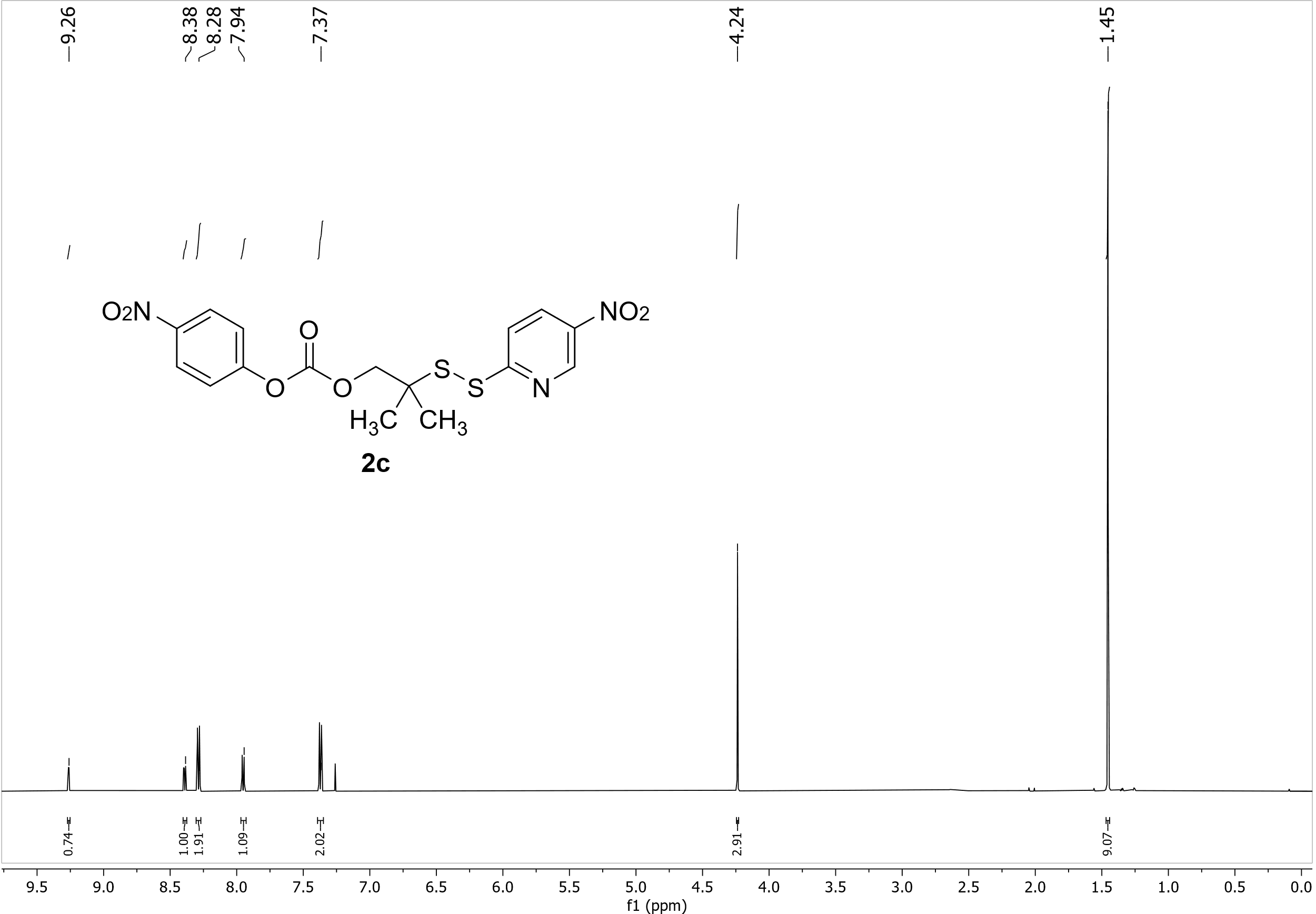

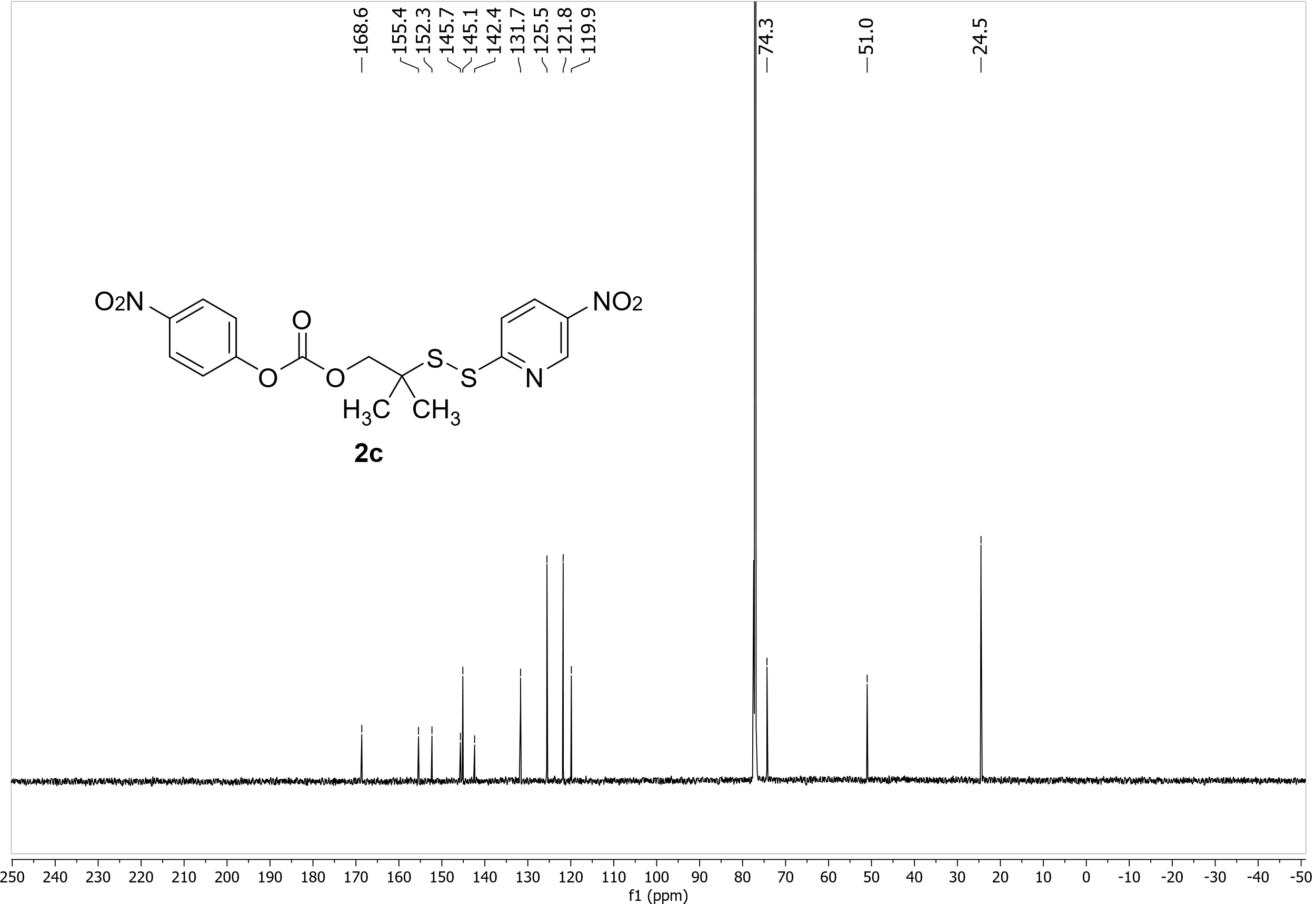

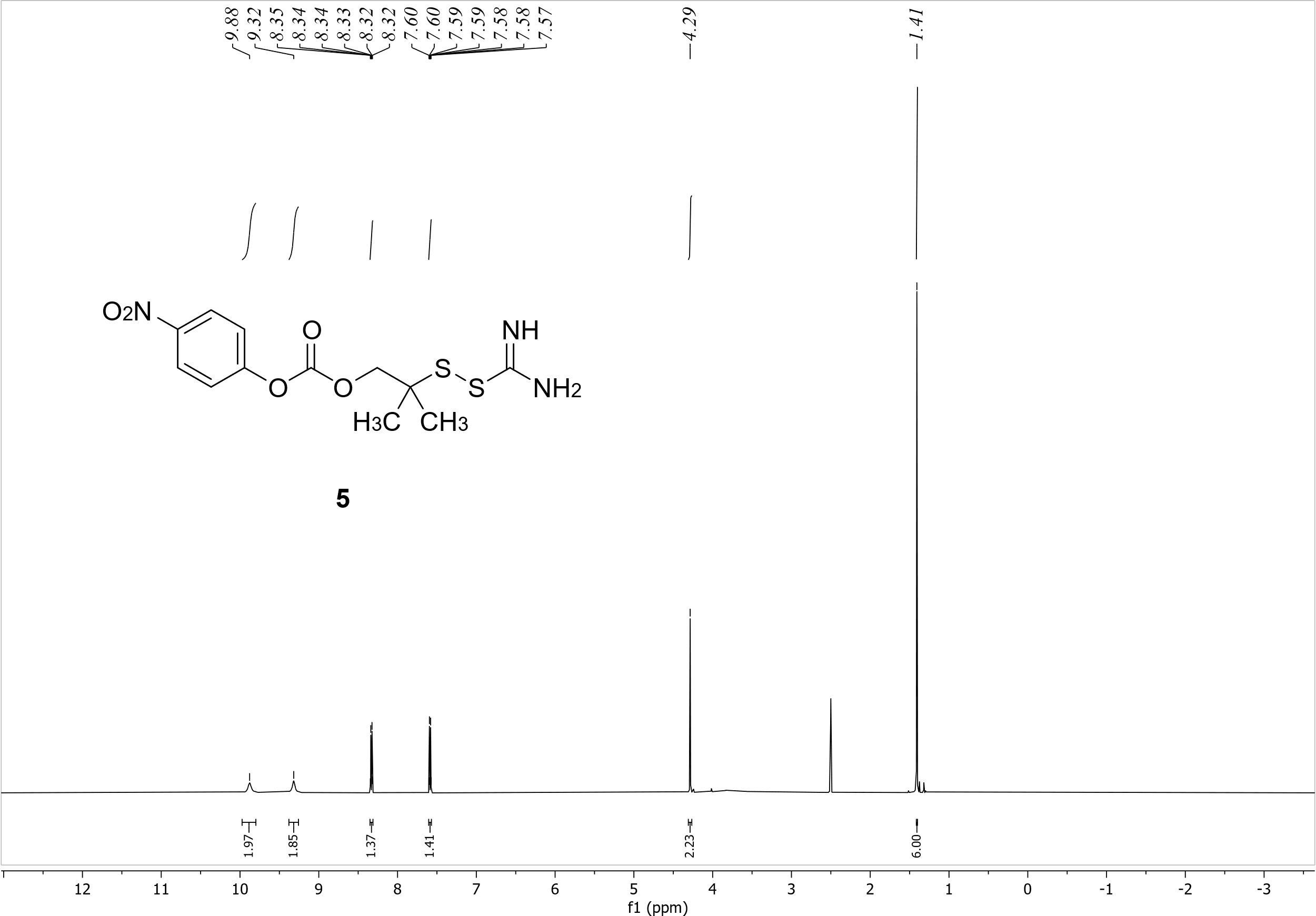

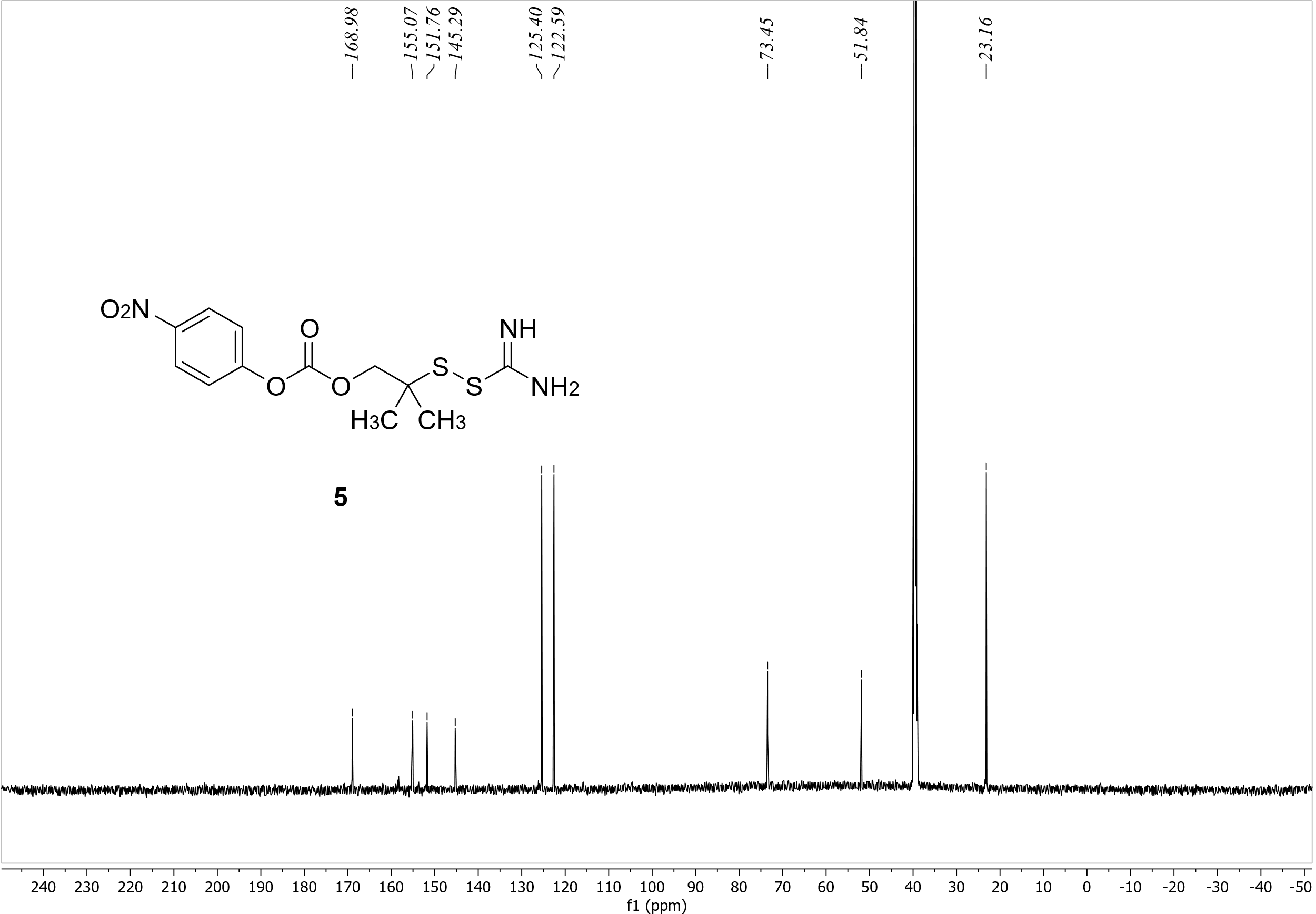

Supplement: Supplementary file 1 — Supporting Information [file ANIE-64-e202506802-s001.docx]
